# Integrated impact quantification of proposed hydropower plants in Africa

**DOI:** 10.1038/s41598-026-59624-2

**Published:** 2026-06-26

**Authors:** Rebecca Peters, Jürgen Berlekamp, Klement Tockner, Christiane Zarfl,

**Affiliations:** 1 Department of Geosciences, Eberhard Karls University of Tübingen, 72076 Tübingen, Germany; 2 Institute of Environmental Systems Research, University of Osnabrück, 49076 Osnabrück, Germany; 3 Senckenberg – Leibniz Institution for Biodiversity and Earth System Research, 60325 Frankfurt am Main, Germany; 4 Faculty of Biological Sciences, Goethe-University, 60323 Frankfurt am Main, Germany; 5 Cluster of Excellence (EXC 3121): TERRA – Terrestrial Geo-Biosphere Interactions in a Changing World, Eberhard Karls University of Tübingen, Tübingen, Germany

**Keywords:** Environmental impact, Energy supply and demand

## Abstract

Among renewable energy sources, hydropower has been the most economical and well-established technology for decades. However, the construction of hydropower plants (HPPs) may have (unknown) cumulative ecological and socioeconomic ramifications in the short and long term. In Africa, 673 large HPPs are proposed. If implemented, they will alter all major river networks through dam construction and reservoir inundation, although the actual extent remains unknown. This study conducts an integrated assessment of the impacts of all proposed HPPs at both basin and continental scales. Projected reservoir areas were overlaid with spatially explicit datasets on megafauna abundance, protected areas, cropland, and human resettlement. We further calculated indices of river regulation and fragmentation, as well as potential sediment entrapment and evaporation associated with the projected reservoirs. By integrating these indicators, we identified 102 HPPs that fall within the top quarter of projects with the greatest potential overall impact. HPP capacity size alone proved to be an inadequate impact indicator, as underlined by the highest- and lowest-ranked HPPs, both of which exhibited comparably low capacities. A sensitivity analysis revealed that the ranking depends on both the number of HPPs considered and the selection of indicators included in the analysis. This study provides evidence-based information to support decision-making when balancing renewable electricity needs against the environmental and socioeconomic impacts of HPP development at basin and continental scales.

**Supplementary Information:**

The online version contains supplementary material available at 10.1038/s41598-026-59624-2.

## Introduction

During the past decades, the dominance of fossil fuel in meeting electricity demands has generated additional stress on Earth’s natural systems, which are already under pressure from human activities such as overexploitation, land use change, and pollution. Hence, mitigating global warming has become a central objective of the United Nations Agenda 2030, reflected in Sustainable Development Goals 7 (“Affordable and Clean Energy”) and 13 (“Climate Action”)^1^.

According to the International Energy Agency (IEA), renewable energy sources including hydro-, solar, and wind power must substantially expand to meet long-term climate goals, while satisfying increasing energy and electricity demands^2^. For example, in sub-Saharan Africa, 80% of newly installed electricity capacity is projected to come from renewable resources by 2030. A significant driver of this transition is China’s announcement to cease support for coal-fired power plants upon the completion of all plants currently under construction^3^. At the same time, African countries are not only challenged to phase out fossil fuels but also to provide electricity access to a continuously growing human population^4^. Today, hydropower has the highest share among renewables on the continent, accounting for 17% of Africa´s total electricity generation. This share is likely to increase to more than 23% by 2040^5^. However, its contribution to national electricity mixes varies considerably across Africa and exceeds 80% in countries such as the Democratic Republic of Congo, Ethiopia, Malawi, Mozambique, Uganda, and Zambia^5^.

**Fig. 1:**
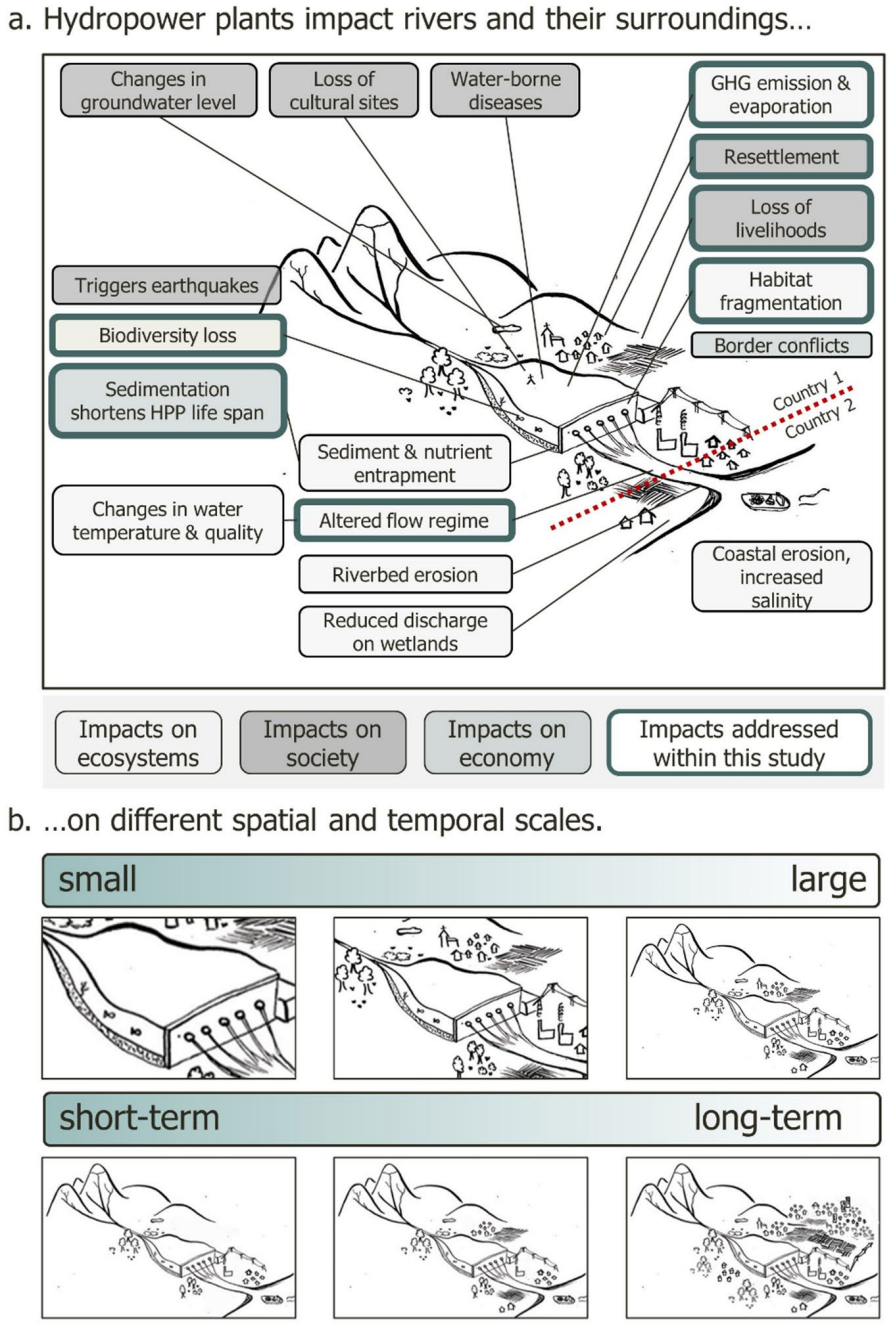
Ecological and socioeconomic impacts induced by hydropower construction. (**a**) Overview of which hydropower plant component induces which impact. Classification is related to the direct potential impact, e.g., biodiversity loss is primarily an impact on ecosystems, but, of course, indirectly also affects society and economy. GHG: greenhouse gas. Adapted from Peters et al. (2021). (**b**) Different spatial and temporal scales on which these impacts can be observed and measured.

Hydropower has a long history as a source of electricity generation. In Africa, for example, the first hydropower plants were built at the beginning of the twentieth century^6^. Although hydropower is a renewable energy source, it is not climate-neutral^7^. Moreover, a large body of research has documented its diverse ecological and socioeconomic consequences (Fig. 1).

The growing demand for renewable electricity has triggered a global boom in dam construction and reservoir inundation^8^. Concurrently, debate over the multifaceted impacts of hydropower development has intensified^9,10^ because hydropower impacts the integrity of coupled terrestrial, freshwater, and coastal ecosystems as well as human well-being. Flow and sediment connectivity are reduced, geomorphology altered^11^, biogeochemical processes truncated^7^, and biodiversity threatened due to habitat loss, fragmentation, barriers to animal migration, species invasion, and over-exploitation^10,12,13^. In addition, dam-induced human resettlement can provoke social conflicts, particularly when affected communities are inadequately informed or insufficiently compensated for losses of livelihoods and land^14,15^. Furthermore, reservoir inundation may increase the transmission of water-related diseases. For example, increased malaria prevalence has been reported in the vicinity of the Kariba Hydropower Plant on the Zambezi River following reservoir inundation^16^.

Insufficient planning and inappropriate site selection have frequently led to project cost overruns and construction delays, thereby amplifying ecological and socioeconomic impacts^17^. Moreover, inadequate site selection can even trigger earthquakes^18^. Hydropower infrastructure can transform entire landscapes; for example, a change in landscape aesthetics may cause public resistance and influence national and international politics^19^. Finally, as about 60% of large rivers cross country borders, hydropower development may lead to international conflicts^20–22^. The ecological and socioeconomic ramifications of HPPs are manifold and vary in time and space (Fig. 1)^23,24^. The interruption of the water course and reservoir creation cause cumulative effects because each additional barrier further alters the flow, thermal, and sediment regimes of the river network^25^. Reservoirs may also enhance greenhouse gas emissions through the decomposition of inundated organic matter^26^. Furthermore, hydropower development is also associated with the downgrading, downsizing, and degazettement of protected areas, including nature reserves and national parks that play a critical role in biodiversity conservation^27,28^. Globally, only 37% of rivers longer than 1,000 km remain free-flowing^27^. At the same time, climate change is increasingly affecting both existing and planned hydroelectricity production^28–30^. For example, the growing frequency of severe droughts has made hydroelectricity unprofitable in affected regions^31^. Moreover, in Africa, 60% of the installed hydropower infrastructure has aged and requires maintenance work and plant updates to optimise electricity production^32^. While the construction of HPPs continues to be the subject of multifaceted scientific debates^9^, the International Hydropower Association has developed guidelines to help project developers incorporate environmental and socioeconomic considerations into the planning process. As such, the Hydropower Sustainability Standard (HSS) is considered an advanced version of the established Hydropower Sustainability Assessment Protocol (HSAP), enabling independent sustainability rating of hydropower projects^33,34^. Unlike the 2010 HSAP, the 2021 HSS goes one step further by not only rating but also certifying a project’s sustainability performance^33,34^. The HSS successfully integrates multiple and complementary ecological and socioeconomic indicators. However, its application remains limited to individual projects and does not account for the cumulative impacts of multiple HPPs on the integrity of entire river networks. There is an urgent need to move beyond individual project assessments towards integrated renewable energy planning at the river network scale in order to adequately integrate environmental and socioeconomic benefits and risks^35,36^.

River basin organisations (RBOs) have been established in many transboundary systems to coordinate the governance of various water-resource interests. Nevertheless, power dynamics and divergent stakeholder priorities often lead to decisions that favour HPP construction while (unintentionally) neglecting the cumulative impacts across entire river networks^37^.

In Africa, 673 new HPPs, each with a planned capacity of ≥ 1 MW, have been proposed. Of these, 243 are confirmed to operate with reservoir and dam, 149 are classified as run-of-river or pumped-storage facilities, and for 281 the operational type remains unknown^6^. This figure reflects the strict inclusion criteria of the RePP Africa database, which requires the planning status to be confirmed by at least two independent sources. To quantify the potential cumulative impacts of these proposed sites for HPPs at the river network scale, the present study applies an integrated impact quantification approach on HPP construction across Africa. The specific aims of this study are to: (1) assess and integrate ecological and socioeconomic impacts associated with proposed dam locations across Africa; (2) rank all proposed HPPs at both basin and continental scales; and (3) quantify the effect of the selection of indicators on the total ranking. The results will highlight the importance of using complementary impact indicators and demonstrate how scale effects influence prioritization outcomes. By doing so, the study provides a discussion basis on site selection for future HPP construction in Africa and beyond.

## Materials and methods

### Underlying hydropower plant data

Data on existing and proposed HPPs in Africa were obtained from the Renewable Power Plant Database for Africa (RePP Africa)^6^. This database includes HPPs with a capacity larger than one MW and differentiates between the reservoir, run-of-river, and pumped storage operating types. For complementing the HPP characteristics, projected reservoir areas for the proposed HPPs (types reservoir and unknown) were estimated with the Python Toolbox DamTools for ArcGIS Pro^38^. For the reservoir estimation, the flooding of the upstream digital elevation model (DEM) at a dam position is simulated up to the reservoir water level that is limited by the dam height. The simulation uses data on river segments from the RiverATLAS^39^, major river basins from the HydroBASINS database^40^, simulated annual mean natural discharge data with 15″ resolution from the Water GAP 2.2 model^41^, and the SRTMGL3 3″ DEM^42^. The reservoir delineation of the HPPs as included in the Renewable Power Plant Database (RePP) Africa resulted in two datasets for further analysis: Dataset 1 includes 507 HPPs of type reservoir and type unknown for which reservoir delineation was successful. Seventeen plants out of the initial 524 HPPs were excluded due to failed reservoir delineation. Four of these 17 plants are projects with capacity updates or other HPPs that operate at existing reservoirs and will not cause an inundation of new areas. We tested sensitivity by running all calculations excluding HPPs of type unknown, further referred to as dataset 2 with a total of 236 data entries. Seven HPPs were excluded out of the initial 243 entries due to failed reservoir delineation (Tab. S1).

Since impact quantifications are to be conducted on a river system scale, some of the selected impact indicators as outlined below require details on dam location and reservoir area also for already existing HPPs and existing other dam infrastructure (e.g., for irrigation or drinking water purposes). RePP Africa includes information on reservoir areas for HPPs only; therefore, we additionally used data on existing dams and reservoirs with main purposes other than electricity generation from the Global Reservoir and Dam Database (GRanD))^43^.

### Indicator selection

Indicators for potential HPP impacts were selected based on an approach introduced by Peters et al.^6^: First, the set of selected indicators represented measurable ecological and socioeconomic variables to underpin the idea of quantifying potential consequences of dam locations in the frame of sustainability. We do not add the dimension of changing environmental conditions over time to treat all indicators equally, i.e. they reflect the current knowledge and available data, and to avoid a bias in including additional uncertainty resulting from assumptions about e.g. hydrological dynamics with high temporal resolution. Second, comprehensive and high-resolution spatial data were available via open-access platforms to ensure transparency and reproducibility.

Therefore, the following eight indicators were selected exemplarily for this study:

1. River Regulation—change in average annual river flow due to dam building; given by an index in % (ecological indicator)
2. River Fragmentation—degree of fragmentation of the river network into smaller river stretches due to dam building; given by an index in % (ecological indicator)
3. Sediment Entrapment—retention of river sediments in the reservoir due to the dam itself and the reduced water flow velocity in the reservoir (compared to a river); the indicator value is given in the resulting lifespan of the reservoir in years, i.e., the number of years until the reservoir is filled up with sediments if no sediment removal is implemented (ecological and economic indicator)
4. Megafauna—overlap of proposed HPP dam sites with IUCN data on areas of presence of freshwater megafauna species as an indicator for the potential threat of dam construction and reservoir inundation on biodiversity; given in species number (ecological indicator)
5. Protected Area—overlap of the projected HPP reservoir with protected areas as an indicator for potential HPP impacts on areas of “high ecological value”; given in square kilometres (ecological indicator)
6. Land Use Change—overlap of the projected HPP reservoir with cropland to indicate a potential conflict with food production; given in square kilometres (social and economic indicator)
7. Resettlement—potential resettlement of people living in the area of the HPP reservoir to be flooded; given in number of people (social indicator)
8. Potential Evaporation—change in potential evaporation due to turning a flowing river into a (almost) standing water with a large water-atmosphere interface that favours evaporation; given in cubic kilometres per year (ecological indicator)

While the indicators Megafauna, River Regulation, River Fragmentation, and Sediment Entrapment measure effects that can be traced back directly to the construction of an HPP (including a dam wall as a barrier), the indicators Protected Area, Land Use Change, Resettlement, and Potential Evaporation depict indirect effects caused by the flooding of an area (projected reservoir area).

Depending on the indicator, alterations accumulate and/or are delayed in time, causing synergistic effects on larger spatial and temporal scales. All calculations to quantify the selected impact indicators for each proposed HPP in both datasets were carried out and their results visualised with ArcGIS Pro 2.9.4^44^.

### Quantification of potential impacts of proposed hydropower plants according to selected indicators

For the indicators River Regulation and Fragmentation, the River Regulation Index (RRI) and the River Fragmentation Index (RFI) were calculated, respectively. RRI and RFI include the current degree of regulation and fragmentation caused by existing HPPs and other dam infrastructure. Both indices were calculated by applying a methodology developed by Grill et al.^45^. The basis for these calculations is data from the HydroSHEDS database^46^ and the HydroBASINS dataset (Level 3, Level 4)^40^. Discharge data is obtained from the WaterGAP model^41^. To calculate the number of years until the reservoir would be entirely filled with catchment-eroded sediments (Sediment Entrapment), a revised version of the Universal Soil Loss Equation (USLE) considering the entire basin with existing reservoirs of all purposes and projected HPP reservoirs was used. The USLE, an empirical model designed to predict the annual average soil erosion from runoff using information on soil properties, rainfall, topography, and land use, is used in combination with a geographic information system (GIS)-based Index of Connectivity (IC) to estimate the fraction of catchment-eroded material that is likely to reach the fluvial network and ultimately be trapped behind the dam wall^47,48^. The IC integrates an upslope component and a downslope component. The upslope component represents the erosion potential of the contributing area as a function of catchment area, average slope gradient and land-use impedance (USLE C-factor). The downslope component represents the flow-path-weighted distance to the nearest sink, here defined as the dam location. This combined approach thus estimates sediment delivery to each proposed reservoir, rather than full catchment-scale sediment transport. Existing dams and reservoirs from the GRanD database were included into the IC calculation as intermediate sinks within the flow network, thereby accounting for the sediment-trapping effect of upstream infrastructure on the connectivity of eroded material to downstream proposed HPPs^49^. This combined USLE–IC approach targets the physical soil-erosion process and the connectivity of eroded material to the reservoir; it does not simulate full catchment-scale sediment transport or yield and neglects instream sediment dynamics, and the resulting values therefore carry greater uncertainty than the flow-based indices RRI and RFI. Accordingly, the indicator is intended for the comparative ranking of proposed HPPs rather than for the precise quantification of sediment volumes delivered to each reservoir^50^.

Impact calculations of all other single indicators were based on projected reservoir areas and considered proposed HPPs only. To estimate the number of megafauna freshwater species (≥ 30 kg, Megafauna), including reptiles, amphibians, and mammals that might be affected by dam construction, we intersected data on the presence of freshwater megafauna with the georeferenced dam locations of proposed hydropower plants^12,13^. For the indicator Protected Area, projected reservoir areas were intersected with protected areas using a polygon layer from the World Database on Protected Areas^51^. For the indicator Land Use Change, we used land cover maps from 2015 (Land Cover Maps v2.0.7) provided by the European Space Agency’s Climate Change Initiative with a resolution of 300 m × 300 m^52^. We extracted cropland as an indicator for food production and overlaid it with the projected reservoir areas.

In order to estimate the number of people that must be resettled from potentially inundated reservoir areas (Resettlement), we overlapped projected reservoir areas with a population raster dataset obtained by the WorldPop Project with a resolution of 3 arc seconds^53^. For the indicator Potential Evaporation, the potential evaporation values [mm/year] were obtained from the FAO GeoNetwork database with a resolution of 10 arc minutes^54^. Evaporation values of the raster feature were bound to reservoir points, and potential evaporation [m^3^/year] was calculated by multiplying each reservoir area [m^2^] with its respective evaporation value [mm/year].

Correlation between independent indicators and projected reservoir areas was tested with R using the rank correlation coefficient Spearman’s rho. This allows us to analyse a potential bias of the ranking of the indicators towards an overlap of the indicators in their interpretation, e.g. “Protected Area” and “Megafauna” as indicators for biodiversity.

### Ranking of proposed hydropower plants considering all indicators and indicator compositions

**Fig. 2:**
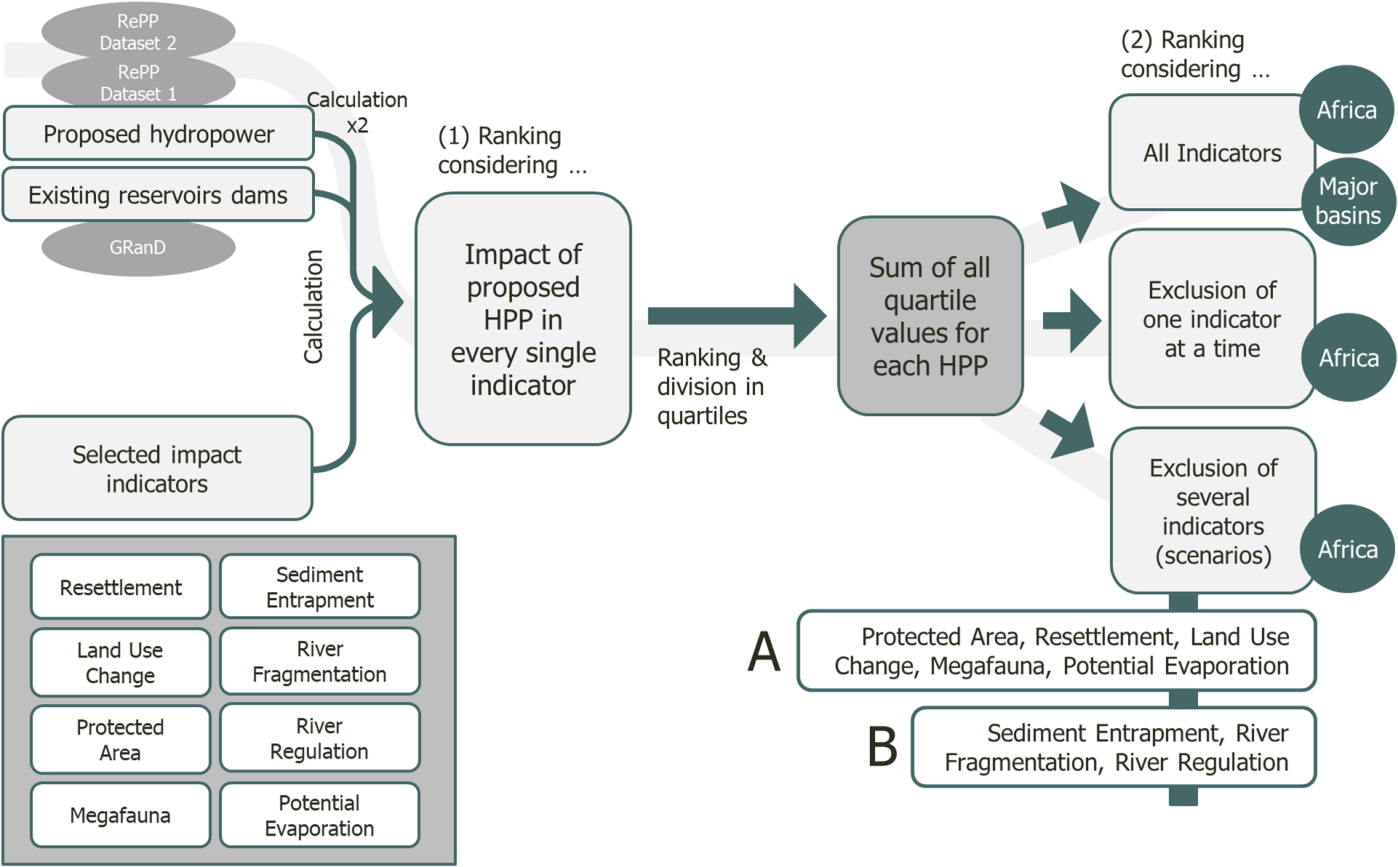
Methodological approach: Impact quantification of proposed hydropower plants and ranking for every single indicator (1) and for different indicator compositions (2). All calculations were run twice: Dataset 1 includes all proposed hydropower plants (HPPs) of type reservoir and type unknown, and dataset 2 includes all proposed hydropower plants of type reservoir. Data on hydropower plants was obtained from the Renewable Power Plant Database (RePP) Africa^6^ and the Global Reservoir and Dam (GRanD) Database^49^. Additionally, we used data on existing dam and reservoir infrastructure for hydropower and other purposes from GRanD^49^.

The impacts of proposed HPPs in Africa were quantified for dataset 1 (HPPs of type reservoir and type unknown), and dataset 2 (HPPs of type reservoir), following the methodological approach summarised in Fig. 2. First, indicators of ecological and socioeconomic relevance were selected and analysed separately. The proposed HPPs were ranked for one indicator at a time according to their calculated impact, and quartiles were determined. Second, following the ranking of each single indicator and the resulting quartile values, every HPP was given the value of the respective quarter they belonged to: from one representing low impact; two, moderate impact; three, heavy impact; to four, severe impact. Third, quarter values were summed for each proposed HPP. Every proposed HPP was ranked according to its overall impact (sum of quarters) (1) for the entire continent and (2) within major basins (Congo, Niger, Nile, Volta, Zambezi). Weighting of indicators could be integrated at this point if stakeholder opinion or discussion outcomes of (local) representatives would support emphasizing of specific indicators, but was neglected here to avoid additional bias. Because impacts of large environmental projects occur in various sectors on different spatial scales and across time, it can be crucial to test if a change of a certain input parameter or an input parameter composition changes the outcome^55^. In order to test the sensitivity of the model to the composition of the indicators, analyses were re-run in a “leave-one-out” fashion (one indicator was excluded at a time, local sensitivity approach^55,56^), and then again through excluding several indicators by creating two different scenarios (Scenario A: local-scale indicators; Scenario B: catchment-scale indicators). After the exclusion of one or more indicators at a time, rankings for both spatial scales, (1) continental and (2) major basins, were created. Results of the impact quantification considering single indicators, all indicators, and indicator compositions were compared among each other (Fig. 2) and visualised with ArcGIS Pro^44^.

We created two different indicator-composition scenarios to better understand if and how the selection of indicators might change the ranking of the proposed HPPs. Scenario A includes the indicators Protected Area, Resettlement, Land Use Change, Megafauna, and Potential Evaporation, which assess the direct effect of reservoir inundation and dam construction on a local scale. Scenario B includes the indicators River Regulation and Fragmentation as well as Sediment Entrapment, all of which are consequences of dam construction. All three indicators assess impacts that cause cumulative system alterations at a large scale, and their calculation considers basin-wide effects of existing dams as well as the complementary building of new ones. The created scenarios are examples that intend to show that rankings at basin or continental level generally depend on the selection of indicators.

## Results

### Quantification and ranking for selected indicators

If all proposed HPPs were constructed, the degree of regulation, expressed as the River Regulation Index (RRI), would range across African sub-catchments from 0 to 1265% for dataset 1 and from 0.8% to 1226% for dataset 2. The wide range corresponds with the results of Grill et al. (2015), who introduced the RRI as a method to quantify full-basin impacts in a single index. The index is calculated by weighting the degree of regulation of each river reach by its corresponding river volume and subsequently averaging these values across the entire basin. The degree of fragmentation, expressed as the River Fragmentation Index (RFI), of sub-catchments would range from 0.04% to 92% for dataset 1 and from 0.04% to 81% for dataset 2. RRI and RFI values peaked in the upper Niger, Nile, and Zambezi basins (Fig. S1, S2; Tab. S2, S4). A total of 2,191 million tons of sediment per year was calculated to be trapped by reservoirs (dataset 2: 1,490 million tons per year, Fig. S3). Under these conditions, the estimated lifetime of the HPPs, defined as the time required until the reservoir would be filled with sediments, could be as short as one year. These estimates neglect sediment-management measures such as sediment flushing. Seventeen proposed HPPs (> 10 MW) with a calculated reservoir lifespan of less than one year were excluded from subsequent analyses, as complete sedimentation of a reservoir of a > 10-MW HPP within such a short period is unlikely and most likely reflects imprecise dam-location coordinates. The combined area flooded by all projected reservoirs was estimated at 46,389 km^2^ (dataset 2: 34,721 km^2^), with a total reservoir volume of 969 km^3^ (dataset 2: 826 km^3^). For both datasets, dam construction and reservoir inundation would impact between one and 11 freshwater megafauna species at individual dam locations (Fig. S4) and overlap with between one and eight protected areas (Fig. S5). In total, 168 projected reservoirs (dataset 2: 84) would overlap with 297 protected areas (dataset 2: 167) and potentially inundate 24,092 km^2^ (dataset 2: 22,310 km^2^), equivalent to 0.4% of Africa´s protected area network. Projected reservoirs would also flood 13,039 km^2^ of cropland (dataset 2: 8,186 km^2^, Fig. S6), and 4.9 million people currently living in potentially flooded areas would need to be resettled (dataset 2: 2.7 million people, Fig. S7). In addition, the projected reservoir surface would increase evaporation by 6 km^3^ per year, which corresponds to a 12% increase relative to evaporation from existing reservoirs and natural lakes combined (Fig. S6).

The calculated impacts of proposed HPPs and the impact rankings of proposed HPPs (both datasets) for each indicator and for all indicators integrated are listed in the Supplementary Information (Fig. S1–S8; Tab. S2, S4).

### Ranking considering all indicators for the entire continent and for major basins

Each HPP with a calculated impact for all eight indicators was included in the integrated impact quantification and the subsequent ranking (Table 1). In total, 448 proposed HPPs of type reservoir and type unknown were included (dataset 1; dataset 2: 211 HPPs).

**Table 1:**
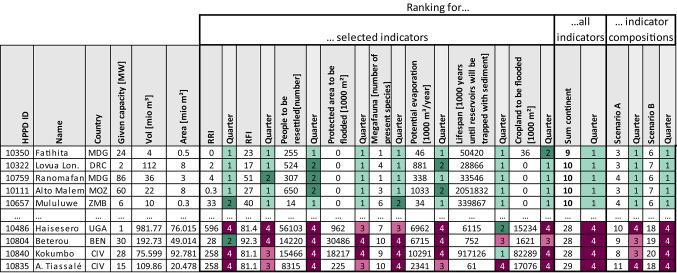
Summary on different rankings for the lowest ranked and top ranked hydropower plants (HPPs) for dataset 1. Quarter values are indicated following the ranking of each selected indicator. A quarter value of one was given for HPPs ranked in the lowest quartile (<Quartile(Q) 1, weak, light mint green), followed by quarter two (Q1–Q2, moderate, sea green), three (Q2–Q3, heavy, pink) and four (>Q3, severe, plum). These quarters are summed up for each HPP (sum continent). Again, quarter values are assigned, considering all HPPs across Africa. For the ranking considering indicator compositions, only a selection of indicators was included to derive quartiles (Scenario A: Protected Area, Resettlement, Land Use Change, Megafauna, Potential Evaporation; Scenario B: River Regulation, River Fragmentation, Sediment Entrapment). Detailed information for all HPPs including dataset 2 is listed in SI1-5).

**Fig. 3:**
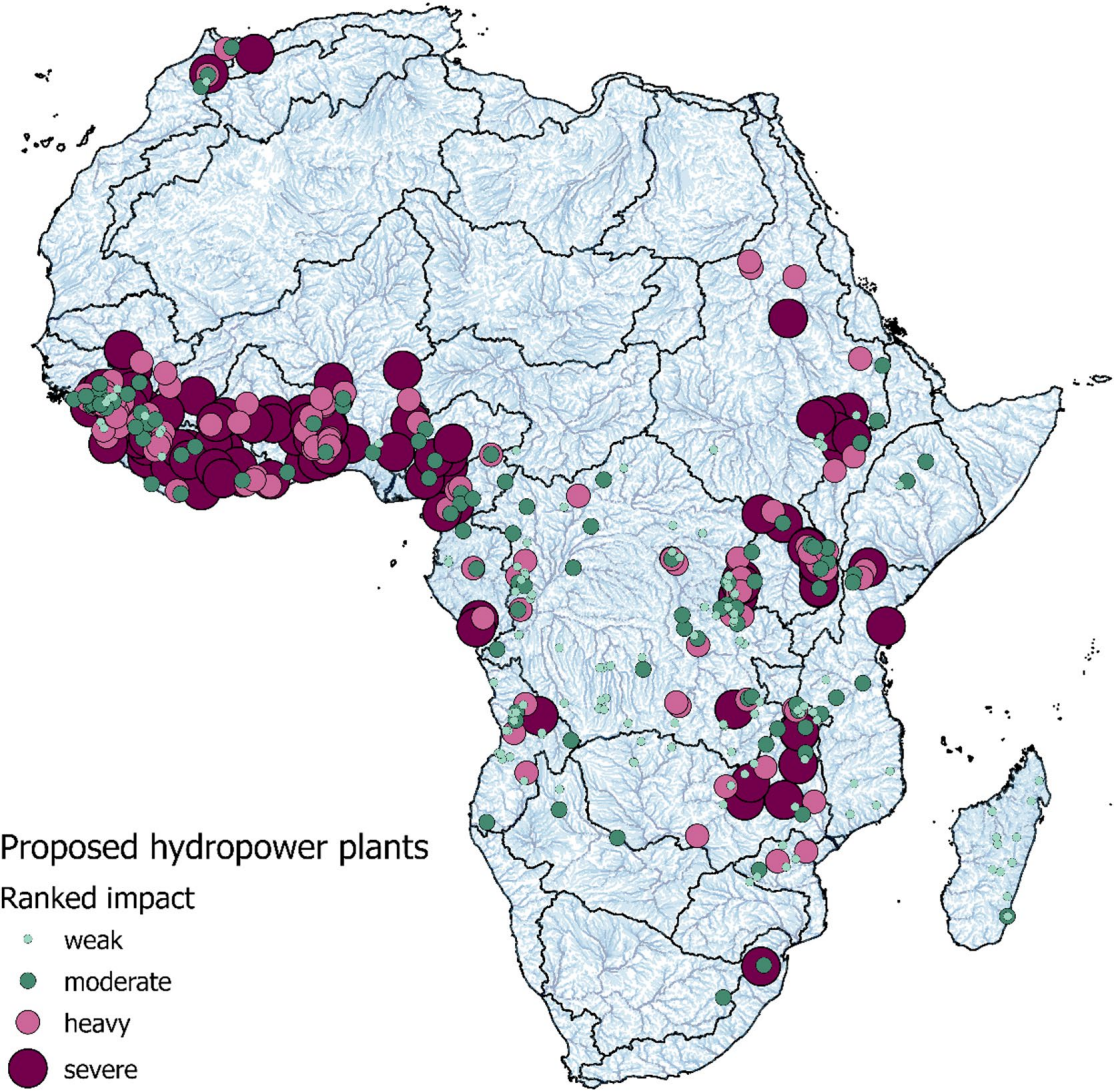
Results of the ranking at continental scale (all indicators) for hydropower plants of type reservoir and type unknown (dataset 1): Circles indicate proposed hydropower plants and size increases with indicated quarter value from weak (< Quartile(Q) 1), moderate (Q1–Q2), heavy (Q2–Q3), to severe (> Q3). Boundaries of major river basins are indicated (HydroBASIN Level 3). Created with ArcGIS Pro 2.9.4, ESRI (https://arcgis.esri.de/arcgis-pro/).

Based on the eight indicators selected, the aggregated impact value could range from eight (i.e., each of the eight indicators has an impact value of 1) to 32 (i.e., each of the eight indicators has an impact value of 4). For individual HPPs (at the continental scale), the calculated total value of all eight indicators combined varied from 9 to 29 for dataset 1 (mean: 19.2 ± 4.1) and from 9 to 27 for dataset 2 (mean: 19.0 ± 3.9) (Fig. 3, S9, S12).

In total, 102 HPPs in dataset 1 and 43 HPPs in dataset 2 were assessed causing severe impacts across the African continent, while 127 (dataset 1) and 61 (dataset 2) HPPs were classified as having low impact (Fig. 3). Overall, 23 countries in dataset 1 and 19 countries in dataset 2 contain proposed HPPs that were assessed as causing severe impacts.

The mean capacity of the twelve highest-ranked HPPs in dataset 1 (total impact values of 27 to 29) was 76 MW, whereas the mean capacity of the ten lowest-ranked HPPs (total impact values of 9 to 11) was 47 MW. Compared with the mean capacity of the complete dataset (152 MW), these values suggest that installed capacity size alone is not an adequate factor to assess an HPP’s impact (Table 1).

Dataset 2 excludes HPPs of unknown type, which are mainly small- and medium-size projects. As a result, the mean capacity in dataset 2 (214 MW) is larger than in dataset 1. Moreover, the difference between the highest- and lowest-ranked HPPs in dataset 2 was less pronounced than in dataset 1. Nevertheless, a remarkable contrast remained, with a mean capacity of 122 MW for the eleven highest-ranked HPPs (total value of 26 to 27) and 198 MW for the nine lowest-ranked HPPs (total value of 9 to 12). These results confirm that HPPs impact and installed capacity are not generally correlated.

**Fig. 4:**
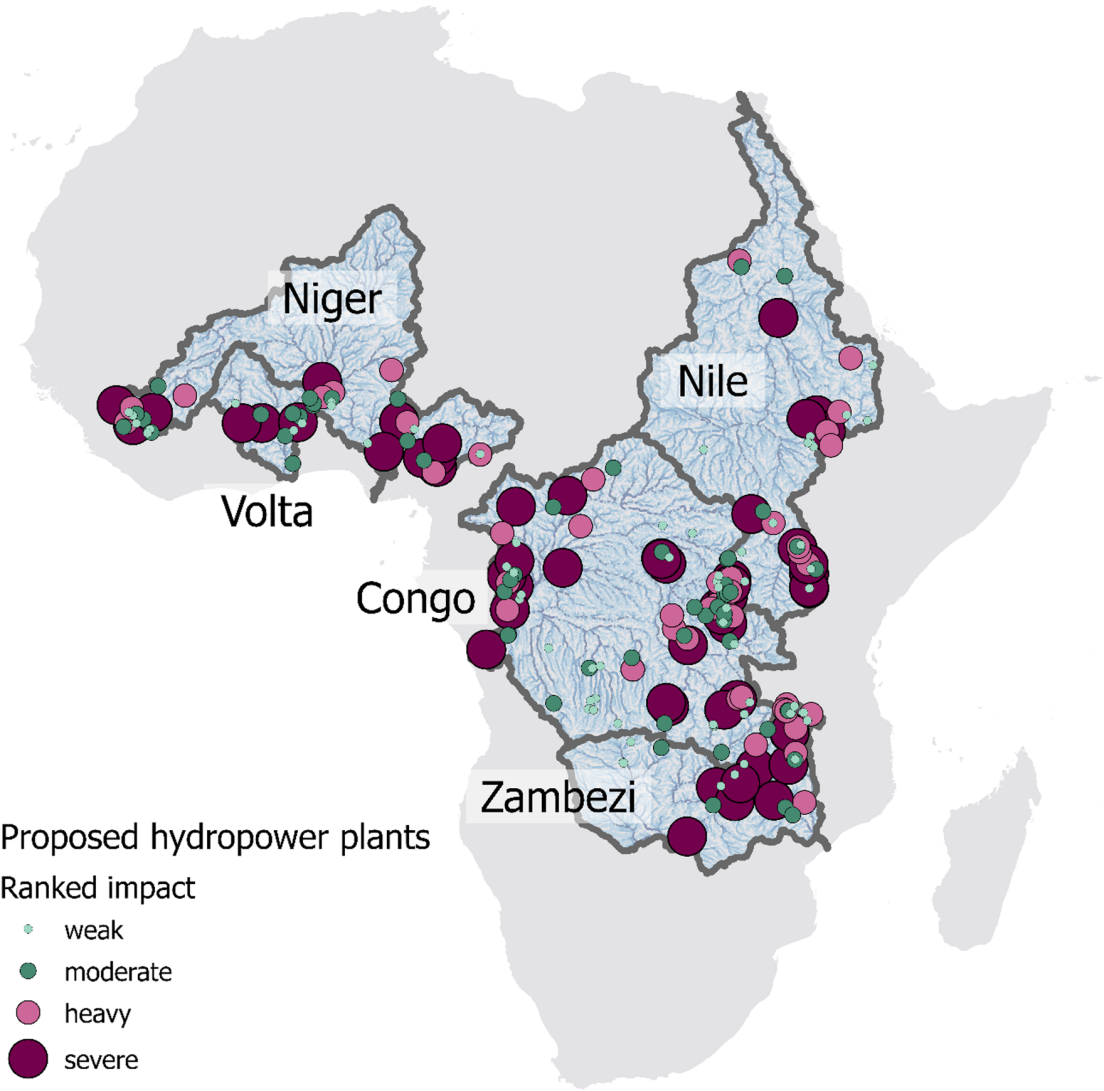
Results of the ranking at basin scale, including all indicators for hydropower plants of type reservoir and type unknown (dataset 1). Basin-scale rankings were conducted for the Congo, Niger, Nile, Volta, and Zambezi river basins (HydroBASIN Level 3). Circles indicate proposed hydropower plants, and circle size increases from weak (< Quartile(Q) 1), moderate (Q1–Q2), heavy (Q2–Q3), to severe (> Q3) cumulative impact. Created with ArcGIS Pro 2.9.4, ESRI (https://arcgis.esri.de/arcgis-pro/).

The overall rankings of all proposed HPPs (i.e., sum of the assigned quarter value per indicator) are listed in the Supplementary Information (Tab. S3, S5). Spearman’s correlation coefficients revealed no strong or very strong relationships among individual indicators (rs > 0.6, rs < − 0.6), with few exceptions (Tab. S6). We conducted an integrated impact quantification for the Congo, Niger, Nile, Volta, and Zambezi basins (Fig. 4). In the continental-scale assessment, 83% (dataset 2: 83%) of proposed HPPs in the Congo Basin were classified as having low to medium impact. However, the basin-scale assessment revealed that 35 of 84 HPPs in dataset 1 and 5 of 12 HPPs in dataset 2 may cause heavy and severe impacts relative to all proposed HPPs within the basin.

This difference is mainly due to the spatial scale at which indicators are aggregated: At the continental scale, quantified impacts are distributed over a wide range of values, with the highest values assigned mainly to HPPs located in the Nile basin and eastern Africa. Simultaneously, most HPPs proposed for the Congo Basin are classified as having low to moderate impacts in the continental comparison. Applying the same ranking method at basin scale changes both HPP rankings and quartile assignments because the assessment is based on a smaller set of HPPs. Consequently, several HPPs located in the Congo Basin are reclassified as having heavy or severe impacts. Basin-scale results for dataset 2 are presented in the Supplementary Information (Fig. S10).

The basin-scale assessment shows that several proposed high-impact HPPs in the Congo Basin received comparatively lower rankings in the continental assessment. According to the continental ranking, only one proposed HPP in the Congo Basin was classified as having severe impact (N = 448). In contrast, the ranking of scenario A assigned a total of 11 proposed HPPs to the highest impact category (N = 448). Considering only HPPs within the Congo Basin, 20 of the 84 proposed HPPs were classified as causing severe impacts (N = 84). The Congo Basin contains large regions that are sparsely populated, leading to lower impacts related to human resettlement and cropland loss than in the Nile, Niger, Volta, and Zambezi basins. At the same time, the Congo represents one of the last very large free-flowing rivers in the world^27^ and harbours a very high and unique biodiversity^57^. Consequently, an implementation of the 84 proposed HPPs along the Congo River and its tributaries must be carefully evaluated within the broader water-energy-food-biodiversity nexus. If alternative energy options were exploited and electricity shared across borders, the Congo River system could be maintained in a largely free-flowing state.

### Ranking considering different indicator compositions

Not all proposed HPPs classified as causing severe impacts in the integrated assessment received the same outcome for the single indicator ranking. Omitting one indicator at a time caused 83 to 107 HPPs in dataset 1 (50 to 77) to be reassigned to a different quarter (Tab. S3, S5). The ranking proved most sensitive to the removal of the indicators Protected Area and River Regulation, which led to the largest number of quartile reassignments. In contrast, excluding Land Use Change had the smallest effect on the overall ranking.

**Fig. 5:**
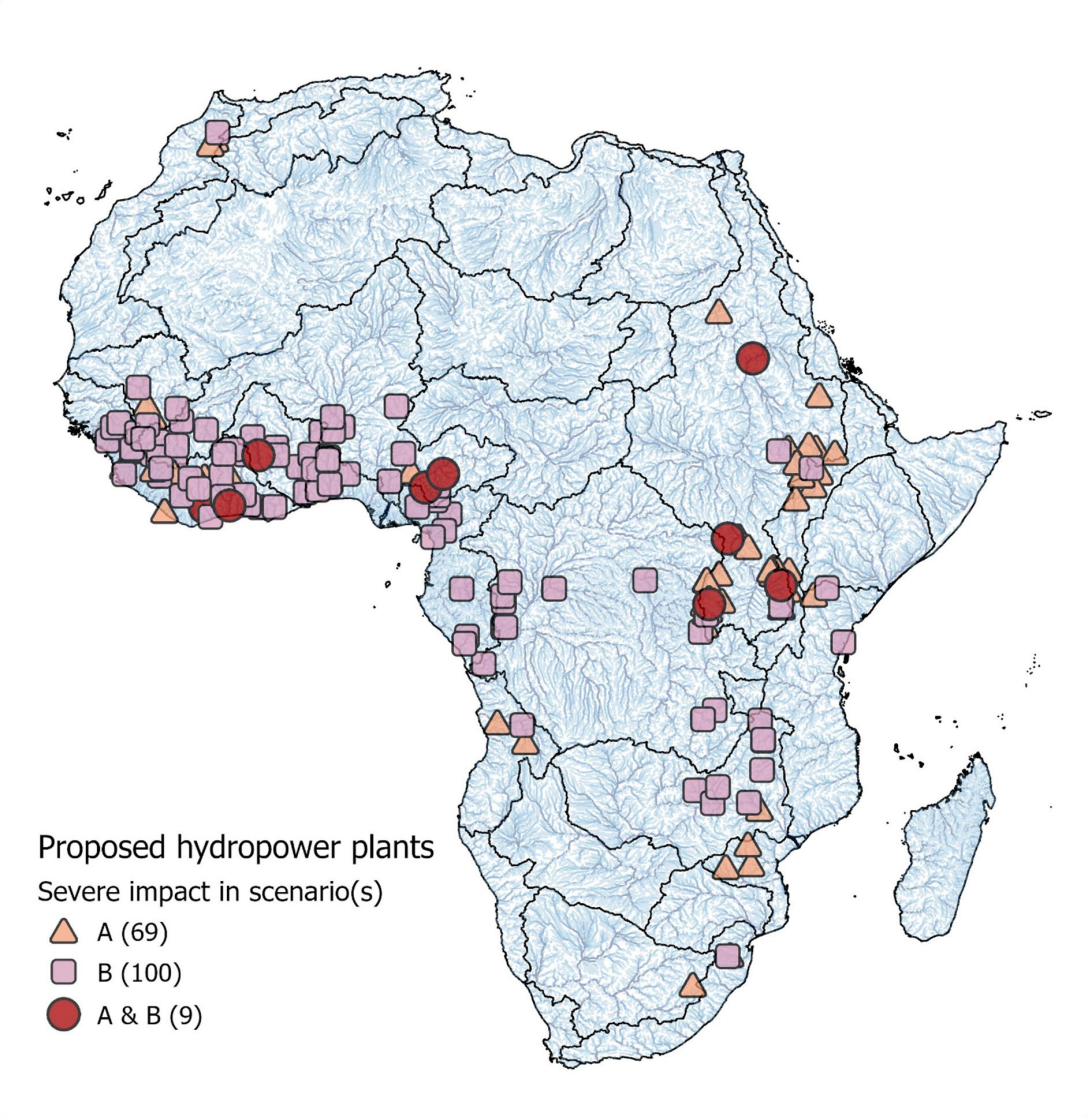
Results of the scenario ranking at continental scale by applying two different scenarios of indicator composition for hydropower plants of type reservoir and type unknown (dataset 1) Scenario A: Protected Area, Resettlement, Land Use Change, Megafauna, Potential Evaporation; Scenario B: River Regulation, River Fragmentation, Sediment Entrapment: Symbols indicate proposed hydropower plants (HPPs) in the top quarter (> Quartile 3; severe impact) of one (A: orange triangle, B: pink), or both scenarios (red circle). Total number of HPPs in each category given in brackets. Created with ArcGIS Pro 2.9.4, ESRI (https://arcgis.esri.de/arcgis-pro/).

Two scenarios were developed to assess how different indicator compositions influence the total ranking of proposed HPPs (Fig. 5). Scenario A includes the indicators Protected Area, Resettlement, Land Use Change, Megafauna, and Potential Evaporation, which serve as proxies for the direct impacts of reservoir inundation and dam construction (Fig. 1). Applying this scenario, HPPs proposed within the Nile Basin were assessed as causing severe impact (i.e., located in the top quarter). Scenario B includes the indicators River Regulation, River Fragmentation, and Sediment Entrapment. These indicators are proxies for impacts measurable at larger spatial scales and capture the effects of dam construction on entire river networks at the basin level. HPPs classified as causing severe impacts under Scenario B were mainly located in West Africa and in the Zambezi and Congo basins (Fig. 5).

A total of nine proposed HPPs were assessed as causing severe impacts under both indicator-composition scenarios: These included the HPPs Sabaloka (205 MW, Sudan), Nandi Forest (50 MW, Kenya), Agbinika (20 MW, Uganda), and Haiserero (1 MW, Uganda) proposed in the Nile Basin; the HPPs Katsina-Ala (109 MW, Nigeria), and Garin Dalli (92 MW, Nigeria) proposed in the Niger Basin; the HPPs Boutoubre (156 MW) and Aderci Tiassalé (15 MW) proposed for Ivory Coast; and the HPP Noumbiel (60 MW, Burkina Faso) proposed for the Volta Basin. Results for dataset 2 are provided in the Supplementary Information (Fig. S11). The comparison confirms that HPPs rankings are influenced not only by the selection of indicators, but also by the number of HPPs projects considered in the analysis.

The nine proposed HPPs identified as causing severe impacts under both indicator-composition scenarios mark locations where local-scale impacts (Scenario A) and catchment-scale impacts (Scenario B) coincide. The spatial distribution of the remaining severe-impact HPPs differ markedly between the two scenarios: Scenario A identifies severe-impact projects predominantly within the Nile Basin, whereas Scenario B highlights projects mainly in Western Africa and the Zambezi Basin. This spatial divergence shows that the dominant type of hydropower impact is not uniformly distributed across the African continent. Local-scale pressures, such as resettlement, land-use change, and impacts on protected areas, tend to concentrate in different regions than catchment-scale pressures, such as river regulation, fragmentation, and sediment entrapment. Consequently, areas experiencing the highest local impacts do not necessarily coincide with those facing the greatest basin-wide ecological alterations.

## Discussion

**Fig. 6:**
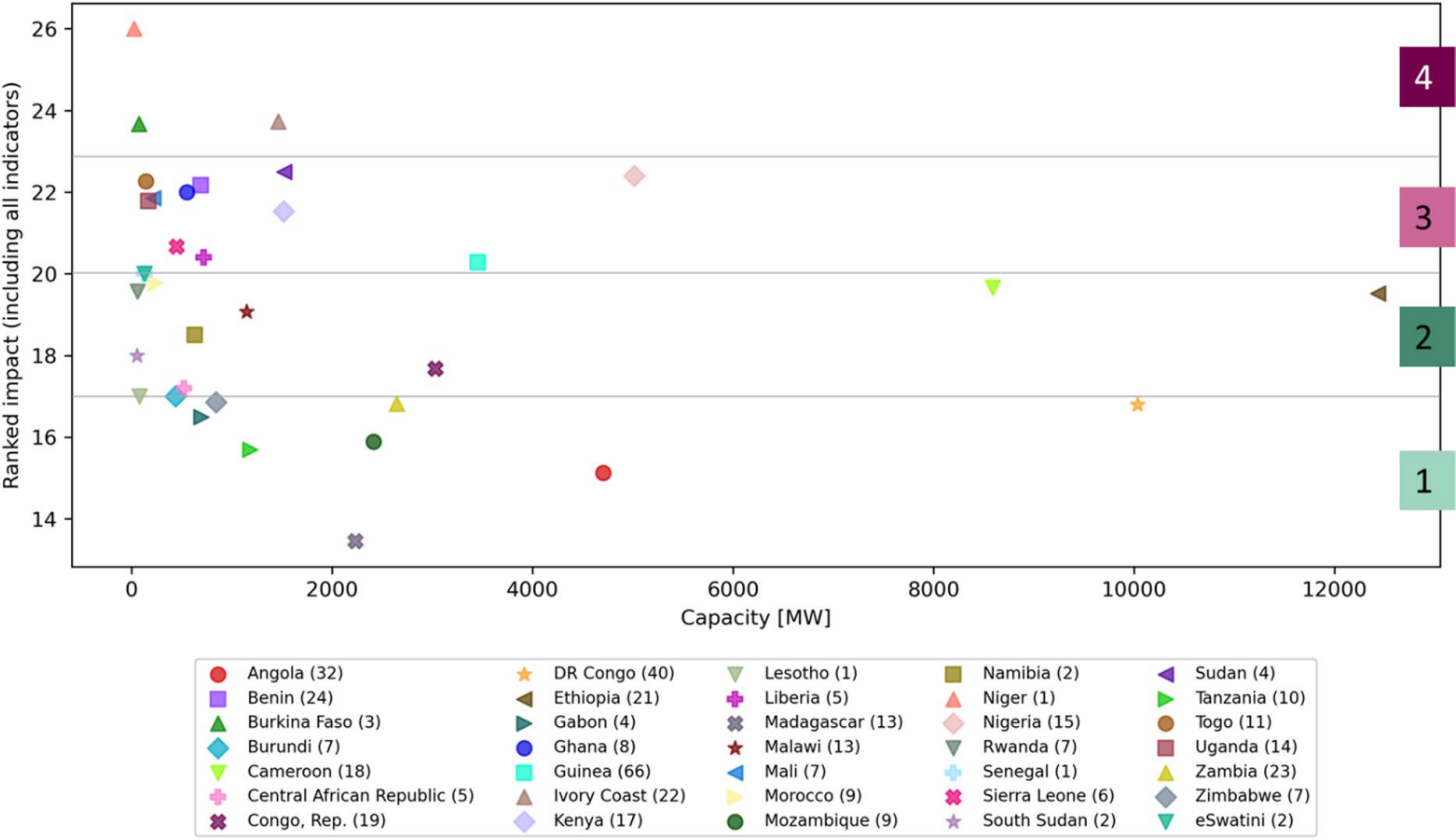
Summary of ranked impact (mean in numbers) per cumulative national capacity (sum in megawatts [MW]) at continental scale (all indicators) for hydropower plants of type reservoir and type unknown (dataset 1): Countries vary by colour and symbol. Grey lines indicate the derived quartiles with indicated quarter values from one (< Quartile(Q) 1, weak, light mint green), followed by quarter two (Q1–Q2, moderate, sea green), three (Q2–Q3, heavy, pink), and four (> Q3, severe, plum). The number of included hydropower plants is given in brackets.

A central finding of this study is that large hydropower capacity is not a reliable predictor of cumulative impact when HPPs are evaluated at continental or basin scales. The average cumulative impact score of the 30 proposed HPPs with capacities ranging from 500 to 7,180 MW was assessed as high (20.6 ± 3.9), whereas the impact of the 116 proposed HPPs with capacities between one and 10 MW was assessed as medium (18.6 ± 3.7). Despite this relatively small difference in impact scores, the 30 largest HPPs would provide approximately 60 times the installed capacity of the 116 smallest projects (< 10 MW). These findings demonstrate that projects with vastly different capacities can be associated with comparable cumulative impacts (Fig. 6). Consequently, installed capacity alone provides limited information on the environmental and socioeconomic consequences of hydropower development. Instead, impacts are strongly influenced by site-specific conditions, highlighting the need to explicitly link energy-generation benefits with ecological and socioeconomic costs when evaluating and prioritizing HPP projects^58^.

The results of the integrated impact quantification, including both indicator-composition scenarios (Scenario A: local-scale indicators; Scenario B: catchment-scale indicators) and the leave-one-out sensitivity analysis, highlight that overall rankings are strongly influenced by indicator selection. In addition, the indicators selected in this study do not represent a full set of potential impact metrics. Depending on the objectives of a given assessment, this framework can be transferred to other river basins (within and outside of Africa), applied at different spatial scales, extended to dams serving purposes other than hydropower (e.g. irrigation, flood control), and adapted to incorporate additional impact indicators, such as changes in river water temperature^67^.

The key strengths of the proposed impact quantification approach are its continental coverage, the integration of complementary ecological and socioeconomic indicators, and its transparency and reproducibility. Among the indicators considered, River Regulation and River Fragmentation are particularly difficult to omit, as they capture the cumulative network-scale effects most distinctive to hydropower development and are often overlooked in individual plant-based EIAs. The approach could be further improved by extending the biodiversity assessment beyond protected areas to include endangered and endemic species and by refining the Sediment Entrapment indicator. Additional indicators related to water availability could also be added, particularly for river flows are critical for local livelihoods. Further indicators may emerge through stakeholder consultations and participatory planning processes.

In addition, indicator weighting could be integrated if stakeholder consultations or discussion outcomes with (local) representatives support placing greater emphasis on specific indicators. herein the present study, however, all indicators were weighted equally to avoid introducing additional bias. The scenario analyses highlight a limitation of conventional project-based assessments: impacts evaluated at the scale of individual HPPs may differ substantially from those emerging when multiple HPPs are considered simultaneously across entire river networks. In this study, HPP rankings depend not only on the selected indicators at different spatial levels but also on the number of HPPs included in the assessment. To address this issue, we applied the same impact-quantification method at different spatial levels and across two datasets. This approach acknowledges the uncertainty associated with proposed HPPs, some of which may not be implemented as proposed or entirely cancelled. Future studies should explicitly examine how both indicator selection and the number of included plants influence ranking outcomes.

The sensitivity of rankings to indicator selection has important implications for the practical application of integrated impact assessments. Different indicator sets can produce divergent rankings, creating the possibility that stakeholders with differing interests may consciously or unconsciously favour indicators that support preferred outcomes (i.e., self-fulfilling results). Standardised and comparable assessment procedures are therefore essential. Rather than concealing the influence of indicator choice, our framework makes it explicit and open to discussion. The selection and, where appropriate, weighting of indicators can be agreed upon by stakeholders affected by hydropower development, including river basin organisations, national energy and environmental authorities, affected communities and conservation bodies.

Used in this way, the continental- and basin-scale rankings provide a shared, evidence-based basis for discussion rather than a prescriptive verdict on individual projects. They can help identify which proposed HPPs warrant more detailed site-specific assessment, where cumulative network-scale effects are likely to be greatest and where alternatives—such as regional electricity sharing to reduce pressure on largely free-flowing systems—merit further consideration. The proposed ranking method should therefore be viewed as a screening and prioritisation tool that complements, rather than replaces, detailed environmental and social impact assessments required for final siting and investment decisions.

Regarding spatial scale, a continental-scale ranking inevitably compares HPPs across environments that are as ecologically distinct as the Sahel, the Congo Basin rainforest, and the East African highlands. A given reservoir area in a biodiverse tropical forest may have fundamentally different ecological impacts than a reservoir in a semi-arid environment, yet both may receive comparable quarter scores if their indicator values are comparable. This limitation is inherent to any continental-scale screening approach. To reduce this bias, we did not use general ecologic categories such as biomes as indicators, but instead relied on variables such as freshwater species numbers and protected areas that represent locations formally recognized as having high ecologic value. Consequently, the ranking should be interpreted as a prioritisation tool that guides more detailed, biome-sensitive local assessments rather than as a definitive measure of absolute ecological impact. The basin-scale assessments (Fig. 4) partly address this limitation by comparing HPPs under more similar conditions, suggesting the approach can be adapted to ecologically more coherent units and potentially to specific biomes.

Although our analyses are restricted to a selected set of indicators and datasets, the results highlight several challenges that emerge when moving from individual plant-based EIAs to large-scale assessments. First, rankings are sensitive to the selection of impact indicators. In the absence of standardized impact assessments, stakeholders with different interests may consciously or unconsciously favour specific indicators that support preferred outcomes, thereby influencing overall rankings. Second, rankings depend on the number of proposed HPPs included in the ranking analysis and, consequently, on the spatial scale of comparison. Furthermore, it is unlikely that all proposed plants will be implemented as currently planned. Nevertheless, considering multiple proposed HPPs simultaneously reveals cumulative and synergistic effects on river connectivity, flow regulation, and sediment entrapment that remain largely overlooked in conventional project-based EIAs. To account for uncertainty in the database, future studies should routinely evaluate the sensitivity of rankings to alternative datasets and project portfolios, as demonstrated here by excluding HPPs with an uncertain operating type (dataset 2).

While the need for renewable electricity undoubtedly exists and hydropower continues to be among the favoured sources of low-carbon energy, existing mechanisms often fall short of capturing the full range of ecological and socioeconomic impacts associated with hydropower development. Ecological and socioeconomic impact assessments of proposed HPPs are typically conducted for individual HPPs and are both time- and resource-intensive^59^. As a result, they are poorly suited to evaluating the potentially cumulative and synergistic impacts of multiple HPPs operating within connected river networks^10,35,36^.

Beyond these methodological limitations, advocates often overestimate benefits and underestimate the far-reaching effects on biodiversity, including impacts on endemic fish species^60,61^. A prominent example for hydropower construction-induced trade-offs on biodiversity is the Julius Nyerere HPP on the Rufiji River in Tanzania. Despite its projected hydropower capacity of 2,115 MW, the project has been strongly criticised because it is located within the Selous National Park, a UNESCO World Heritage Site. Reviews of the project have concluded that existing assessments to not fully capture the irreversible environmental and socioeconomic consequences associated with the construction of the 130 m dam and the 914 km^2^ reservoir^62^. Renowned international organisations such as IUCN and UNESCO have therefore called for independent, integrative, and large-scale assessments that meet international guidelines and standards^63^. In this regard, our results outline the importance of integrated and independent impact assessments that include multiple indicators, cumulative effects of concurrent dam construction, and uncertainty though sensitivity analysis. These findings suggest that basin-scale hydropower planning is both mandatory and feasible.

These findings must also be viewed within a broader policy context. The International Renewable Energy Agency (IRENA) highlights that meeting the Paris Agreement climate targets will require a complete decarbonisation, which, according to IRENA, is only feasible if the globally installed hydropower capacity more than doubles by 2050^64^. Consequently, many current energy policies continue to favour hydropower expansion^6,8^. In Africa, for example, the technically and economically exploitable hydropower potential is calculated to be 6,000 TWh per year, roughly four times current generation levels^65^. However, a study on the economic feasibility of hydropower development under climate change suggests that hardly any new HPPs will be economically feasible in Africa after 2030^66^. Still, 673 HPPs are currently proposed across the continent^6^. This number may appear low for a continent of Africa’s size and its overall hydropower potential. In particular, central African countries depend on hydropower, as it provides up to 80% of the total electricity in the Democratic Republic of Congo, Ethiopia, Malawi, Mozambique, Uganda, and Zambia. More HPPs might be in the pipeline in particular across these countries. However, our data follow strict quality criteria as applied in the RePP Africa database, which requires confirmation of the planning stage by at least two independent sources.

## Conclusions

In Africa, the number of HPPs operating with dams and reservoirs could increase from 247 (2022, including 29 plants under construction) to 771 if all proposed HPPs are constructed (from 203 to 446 for dataset 2, excluding HPPs where the operation type is uncertain). The total capacity would increase from 29 to 159 GW. Such an expansion would substantially alter many of the continent´s major river systems. At the country level, it is becoming evident that electricity planning needs to move from individual plant-based impact screening towards integrated basin-scale planning that considers cumulative ecological and socioeconomic impacts. River basin organisations (RBOs) are well positioned to support such assessments, particularly in transboundary river basins, but currently lack widely adopted and standardised methods for evaluating the combined effects of multiple proposed HPPs.

Based on our findings, we offer the following recommendations to support decision-making on hydropower development and river management:

- Integrated impact quantification, applying a comparable set of criteria, should be conducted at the basin scale for both small and large HPPs. The screening framework should explicitly include measures and scenarios, considering the potential ecological and socioeconomic impacts of the implementation of several proposed HPPs, and serves as a basis for prioritising which sites require more detailed, site-specific environmental and social impact assessment.
- Integrated impact quantification should include sensitivity analyses. Because rankings are influenced by indicator selection, spatial scale, and the set of projects included, policy discussions should explicitly consider how these methodological choices affect outcomes. Scenario comparisons should therefore become an integral component of the planning process.
- Continental-scale screening can complement basin-scale assessments by enabling comparisons across river basins and helping identify development pathways and project portfolios that minimize impacts at scale while meeting energy objectives.

Hydropower will continue to play an important role in Africa’s electricity supply, including through ancillary services such as grid stability that other renewable technologies currently cannot provide to the same extent. The integrated impact quantification approach presented here does not question that role. Rather, it provides a transparent and reproducible basis for identifying projects with comparatively lower impacts and for supporting more informed planning decisions. Our findings demonstrate that hydropower impacts cannot be adequately assessed through project-by-project evaluation alone. Because rankings depend on indicator selection, spatial scale, and the portfolio of projects considered, transparent and standardised basin-scale assessments are required to support energy development while minimising cumulative ecological and socioeconomic impacts.

## Supplementary Information

Below is the link to the electronic supplementary material.

Supplementary Material 1

## Data Availability

Data on existing and proposed hydropower plants from the Renewable Power Plant Database (RePP) Africa is available from 10.6084/m9.figshare.c.6058565.v1 (2022). Data on existing hydropower plants and other dam infrastructure was obtained from the Global Reservoir and Dam Database (GRanD) and is available from https://www.globaldamwatch.org/directory. For complementing the hydropower plant characteristics, projected reservoir areas for the proposed hydropower plants were estimated with the Python Toolbox DamTools for ArcGIS Pro^38^. The toolbox is available upon request. The simulation uses input data from the RiverATLAS ((Linke et al. 2019, available from https://www.hydrosheds.org/hydroatlas), the HydroBASIN database (Lehner 2014, available from https://www.hydrosheds.org/products/hydrobasins), simulated annual mean natural discharge data with 15″ resolution from the Water GAP 2.2 model (Döll, Kaspar, and Lehner 2003, available from https://zenodo.org/records/10026943), and the SRTMGL3 3″ DEM (JPL 2013, available from https://lpdaac.usgs.gov/products/srtmgl3v003/). Further descriptions are provided in detail in the Material and Methods section and cited from publicly available sources.

## Code availability

All processing steps included were realised with ArcGIS Pro 2.9.4 software from ESRI^44^. In-depth information is described in detail in the Material and Methods section. The statistical test (calculation of rank correlation coefficient Spearman’s rho) was implemented in R and follows the common procedure. Python 3.12 was used to create Fig. 6^67^. No new stand-alone programming code was created.
