# Supplementary material for: Integrated impact quantification of proposed hydropower plants in Africa
Source: Sci Rep. 2026 Jun 26;16:19628. doi: 10.1038/s41598-026-59624-2 (PMC13309524; doi:10.1038/s41598-026-59624-2)
Supplement: Supplementary file 1 — Supplementary Material 1 [file 41598_2026_59624_MOESM1_ESM.pdf]

Integrated impact assessment for proposed hydropower plants in Africa

Rebecca Peters[1,†], Jürgen Berlekamp[2], Klement Tockner[3,4], and Christiane Zarfl[1]

1 Department of Geosciences, Eberhard Karls University of Tübingen, 72076 Tübingen, Germany.
2 Institute of Environmental Systems Research, University of Osnabrück, 49076 Osnabrück, Germany.
3 Senckenberg Society for Nature Research, 60325 Frankfurt a. M., Germany.
4 Faculty of Biological Sciences, Goethe-University, 60323 Frankfurt a. M., Germany.

Table S1: Proposed hydropower plants. Overview on sums of included and excluded data points per indicator.

**Data (Status *proposed*)**	**R**	**RU**	**Included**	**Excluded**	**Notes**
Hydropower Type *Reservoir (R)*	x		236	7	Reason for exclusion: Failed reservoir delineation (e. g. due to extension of existing hydropower plant)
Hydropower Type *Reservoir* and *unknown (RU)*		x	507	17	Reason for exclusion: Failed reservoir delineation
River Regulation Index (Level 3)	x		242	1	
River Regulation Index (Level 3)		x	515	9	
River Regulation Index (Level 4)	x		242	1	
River Regulation Index (Level 4)		x	515	9	
River Fragmentation Index (Level 3)	x		242	1	
River Fragmentation Index (Level 3)		x	514	10	
River Fragmentation Index (Level 4)	x		230	13	
River Fragmentation Index (Level 4)		x	510	14	
Protected Area	x		84<br>152*	7	*no overlap with protected areas
Protected Area		x	168<br>339*	17	*no overlap with protected areas
Resettlement	x		236	7	
Resettlement		x	506	18	
Land Use Change (Cropland)	x		236	7	
Land Use Change (Cropland)		x	507	17	
Megafauna	x		236	7	
Megafauna		x			
Sediment Entrapment	x		218	25	Hydropower with projected lifespan of <1 year and >10 MW were excluded.
Sediment Entrapment		x	453	71	Hydropower with projected lifespan of <1 year and >10 MW were excluded.
Potential Evaporation	x		236	7	
Potential Evaporation		x	507	17	
Impact assessment	x		211	32	All hydropower plants with failed assessment of one or more indicators were excluded
Impact assessment		x			All hydropower plants with failed assessment of one or more indicators were excluded

Table S2: Proposed hydropower plants of Type *Reservoir*. Each hydropower plant (HPP) can be identified with the respective HPPD_ID from the Renewable Power Plant Database (RePP) Africa (Peters et al., 2023). Calculation of impact per indicator and resulting quartile (Q) for River Regulation (RRI, HydroBASIN level 04), River Fragmentation (RFI, HydroBASIN level 04), Resettlement (Res), Protected Area (PA), Megafauna (MF), Potential Evaporation (PE), Sediment Entrapment (SE) and resulting Lifespan, and Land Use Change for Cropland (LU_C). Quartile values are indicated with 1 (low impact, ≤1st quartile), 2 (moderate impact, >1st to ≤2nd quartile), 3 (heavy impact, <2nd to ≤3rd quartile), and 4 (severe impact, >3rd quartile).

HPPD_ID	Cap	r_vol	r_area	RRI_04	RRI_04_Q	RFI_04	RFI_04_Q	Res	Res_Q	PA_No	PA	PA_Q	MF	MF_Q	PE	PE_Q	SE	Lifespan	SE_Q	LU_C	LU_C_Q	SUM_Q
	[MW]	[million m³]	[million m²]	[%]		[%]		count		count	[m²]		count		[m3/year]		[t/year]	[year]		[m²]		
10006	1000	x	x	39	2	78	3	x	x	x	x	x	x	x	x	x	x	x	x	x	x	x
10007	470	72	5	39	2	78	3	165	1		0	1	8	4	542766	1	21871669	9	4	0	1	17
10015	1000	112	34	39	2	78	3	37258	4	1	17171062	4	9	4	3589321	3	1127706	264	3	630680	2	25
10016	250	1217	64	39	2	78	3	416	2		0	1	9	4	6777019	3	30	106277653	1	4745438	3	19
10018	1800	1327	67	39	2	78	3	1820	2		0	1	9	4	7272455	3	6498931	541	3	3964642	3	21
10019	640	0	0	39	2	78	3	1	1		0	1	9	4	1852	1	50141	5	4	7349	1	17
10020	940	30	9	39	2	78	3	92	1		0	1	9	4	1006429	2	19010349	4	4	107594	2	19
10021	5	57	21	39	2	78	3	114	1		0	1	7	3	2349331	2	5542075	27	4	19504	2	18
10022	33	36	5	2	1	23	1	291	1		0	1	6	3	654628	2	4635378	21	4	0	1	14
10025	67	2513	245	46	2	49	2	1899	2		0	1	9	4	29551980	4	1152088	5781	2	99076	2	19
10027	72	1993	53	46	2	49	2	23662	4		0	1	10	4	5824247	3	3708997	1424	3	4526014	3	22
10028	485	3604	68	46	2	49	2	5368	3	1	6870267	4	10	4	7820842	3	6020412	1586	2	411023	2	22
10033	1200	1577	85	39	2	78	3	10456	4		0	1	8	4	9431898	3	7186173	581	3	0	1	21
10034	400	1579	108	39	2	78	3	7181	3		0	1	8	4	12341525	4	20	211904235	1	2233150	3	21
10035	600	3251	111	10	1	64	3	1517	2	1	45325338	4	6	3	10663587	4	3347491	2574	2	0	1	20

10167	10145	10127	10107	10106	10105	10104	10103	10095	10093	10049	10047	10043	10038	10037	10036		HPPD_ID
410	28	6747	4800	6706	6684	6970	7180	600	150	1000	1000	300	180	40	1	[MW]	Cap
45	8	704	704	704	704	704	704	834	12089	16535	12646	131	492	90	99	[million m^3]	r_vol
10	1	22	22	22	22	22	22	152	371	996	833	62	65	25	15	[million m^2]	r_area
0	585	6	6	6	6	6	6	10	7	45	45	0	10	0	1	[%]	RRI_04
1	4	1	1	1	1	1	1	1	1	2	2	1	1	1	1		RRI_04_Q
47	63	x	x	x	x	x	x	64	31	45	45	5	64	5	9	[%]	RFI_04
1	2	x	x	x	x	x	x	3	1	1	1	1	3	1	1		RFI_04_Q
3055	20	2890	2890	2890	2890	2890	2890	1031	3616	11907	8437	1081	2375	204	18764	count	Res
3	1	3	3	3	3	3	3	2	3	4	4	2	3	1	4		Res_Q
1	3							5		3	2					count	PA_No
4934732	4321021	0	0	0	0	0	0	103346520	0	234331146	246482932	0	0	0	0	[m^2]	PA
4	3	1	1	1	1	1	1	4	1	4	4	1	1	1	1		PA_Q
9	8	11	11	11	11	11	11	8	7	7	7	9	7	5	5	count	MF
4	4	4	4	4	4	4	4	4	3	3	3	4	3	2	2		MF_Q
960644	144461	2102755	2102755	2102755	2102755	2102755	2102755	15076837	37830034	98590496	82514112	6978078	6746656	2890490	1483545	[m3/year]	PE
2	1	2	2	2	2	2	2	4	4	4	4	3	3	3	2		PE_Q
28	20241914	x	x	x	x	x	90	x	330	2204964	10126398	76234591	7318510	10683518	10507	[t/year]	SE
4295234	1	x	x	x	x	x	20765561	x	96956047	19873	3309	5	178	22	24861	[year]	Lifespan
1	4	x	x	x	x	x	1	x	1	1	2	4	3	4	1		SE_Q
956400	0	15769513	15769513	15769513	15769513	15769513	15769513	560918	52814291	98895767	79625983	295532	1084786	0	12832623	[m^2]	LU_C
2	1	4	4	4	4	4	4	2	4	4	4	2	2	1	3		LU_C_Q
18	20	x	x	x	x	x	x	x	18	23	24	18	19	14	15		SUM_Q

HPPD_ID	Cap	r_vol	r_area	RRI_04	RRI_04_Q	RFI_04	RFI_04_Q	Res	Res_Q	PA_No	PA	PA_Q	MF	MF_Q	PE	PE_Q	SE	Lifespan	SE_Q	LU_C	LU_C_Q	SUM_Q
	[MW]	[million m³]	[million m²]	[%]		[%]		count		count	[m²]		count		[m3/year]		[t/year]	[year]		[m²]		
10174	35	20	2	1	1	9	1	28	1	1	1961687	3	7	3	156324	1	1825	29030	1	0	1	12
10175	73	163	10	1	1	9	1	27	1		0	1	9	4	1019579	2	662424	650	3	0	1	14
10176	180	27	6	0	1	47	1	111	1	1	7248011	4	9	4	586230	2	419	173187	1	181691	2	15
10185	8	1041	17	27	2	61	2	24519	4		0	1	6	3	1728134	2	189988	14518	1	17113734	4	19
10204	10	126	6	27	2	61	2	9924	4		0	1	6	3	720399	2	31	10784008	1	5212775	3	18
10211	1700	571	39	338	4	81	4	1041	2	1	7992235	4	4	2	5289469	3	49915129	30	4	374262	2	25
10212	2000	1536	64	338	4	81	4	10107	4		0	1	3	1	7850060	3	15588270	261	3	15632900	3	24
10213	150	2150	49	292	4	69	3	17217	4		0	1	2	1	6082534	3	42809044	133	3	0	1	20
10214	88	116	18	9	1	6	1	1505	2	1	5124061	4	1	1	2818423	3	74423524	4	4	0	1	17
10215	301	1262	21	338	4	81	4	8693	4		0	1	3	1	2479388	2	1213030	2756	2	14204787	3	21
10232	371	188	4	212	4	75	3	1306	2		0	1	3	1	568337	1	106608	4683	2	2318291	3	17
10233	1600	284	13	338	4	81	4	7762	4		0	1	3	1	1611848	2	240519548	3	4	97033	2	22
10234	278	26	2	338	4	81	4	558	2		0	1	3	1	184313	1	29031	2354	2	0	1	16
10235	265	132	3	338	4	81	4	2063	2		0	1	3	1	291188	1	1895299	184	3	646193	2	18
10236	189	692	7	338	4	81	4	3349	3		0	1	3	1	782156	2	x	x	x	987289	2	x
10238	150	15	2	292	4	69	3	2729	3		0	1	2	1	189238	1	3354485	12	4	478515	2	19

HPPD_ID		10239	10241	10242	10243	10245	10246	10247	10248	10249	10250	10280	10281	10282	10283	10290	10292
Cap	[MW]	423	424	304	467	507	166	1700	560	100	256	34	99	56	25	120	2
r_vol	[million m³]	16322	22889	76735	26	317	64	192	353	173	878	545	300	0	3129	572	0
r_area	[million m²]	466	1400	2310	3	10	1	5	25	5	36	30	29	0	157	19	0
RRI_04	[%]	292	338	338	8	8	8	8	292	52	52	38	585	585	585	585	585
RRI_04_Q		4	4	4	1	1	1	1	4	3	3	2	4	4	4	4	4
RFI_04	[%]	69	81	81	23	23	23	23	69	57	57	52	63	63	63	63	63
RFI_04_Q		3	4	4	1	1	1	1	3	2	2	2	2	2	2	2	2
Res	count	86802	139302	228107	641	2881	514	165	1310	286	1694	16531	97	1	985	33638	236
Res_Q		4	4	4	2	3	2	1	2	1	2	4	1	1	2	4	1
PA_No	count	1	1	1				2	2			1	1	1	2		
PA	[m²]	95257	618928156	844941410	0	0	0	5531660	15861142	0	0	7325705	30651978	41342	158439864	0	0
PA_Q		3	4	4	1	1	1	4	4	1	1	4	4	3	4	1	1
MF	count	2	4	4	4	7	6	6	5	2	2	2	5	5	5	6	5
MF_Q		1	2	2	2	3	3	3	2	1	1	1	2	2	2	3	2
PE	[m3/year]	54336092	181219462	299009007	368146	1347034	160893	585186	3658979	807965	5630728	4537510	3995771	3472	21040993	2547445	24309
PE_Q		4	4	4	1	2	1	1	3	2	3	3	3	1	4	2	1
SE	[t/year]	3539782	615	x	4028602	12171336	2662555	86554	11603308	1698063	701194	216667	2064545	118	1480	3144740	2982842
Lifespan	[year]	12219	98559989	x	17	69	64	5865	81	270	3317	6671	385	1710	5602198	482	0
SE_Q		2	1	x	4	4	4	2	4	3	2	2	3	2	1	3	4
LU_C	[m²]	262473641	590652171	987081649	0	0	0	0	0	0	403110	26007559	19961627	21054	148376783	21662990	193890
LU_C_Q		4	4	4	1	1	1	1	1	1	2	4	4	2	4	4	2
SUM_Q		25	27	x	13	16	14	14	23	14	16	22	23	17	23	23	17

10360	10358	10357	10356	10348	10343	10342	10323	10322	10314	10311	10309	10301	10300	10299	10293		**HPPD_ID**
41	155	43	105	300	600	580	240	86	693	90	19	20	45	80	50	**[MW]**	**Cap**
2033	107	73	102	93	505	5	13	36	1169	419	1	109	0	450	291	**[million m^3]**	**r_vol**
260	34	24	33	1	39	1	1	3	76	28	0	2	0	8	14	**[million m^2]**	**r_area**
107	0	0	0	0	4	4	0	4	87	87	87	292	292	292	585	**[%]**	**RRI_04**
3	1	1	1	1	1	1	1	1	3	3	3	4	4	4	4		**RRI_04_Q**
6	46	46	46	46	51	51	x	51	57	57	57	69	69	69	63	**[%]**	**RFI_04**
1	1	1	1	1	2	2	x	2	2	2	2	3	3	3	2		**RFI_04_Q**
81986	4607	2517	4376	320	2994	111	320	307	2968	2174	1863	3587	183	5370	2700	**count**	**Res**
4	3	3	3	1	3	1	1	1	3	2	2	3	1	3	3		**Res_Q**
						1			2				1	1	1	**count**	**PA_No**
0	0	0	0	0	0	865896	0	0	14143859	0	0	0	29928	9752066	10101411	**[m^2]**	**PA**
1	1	1	1	1	1	3	1	1	4	1	1	1	3	4	4		**PA_Q**
3	1	1	1	1	1	1	1	1	3	4	4	4	3	3	6	**count**	**MF**
1	1	1	1	1	1	1	1	1	1	2	2	2	1	1	3		**MF_Q**
33034776	3703440	2630029	3619914	119332	4227982	113916	88102	337608	13157733	4365786	26607	304471	2086	1045401	1961355	**[m3/year]**	**PE**
4	3	3	3	1	3	1	1	1	4	3	1	1	1	2	2		**PE_Q**
x	83	468	12874384	2123653	445	4966	6851537	2848	2617580	2618836	116676	230746	82257	104972	679036	**[t/year]**	**SE**
x	3389135	410208	21	116	3004579	2701	5	33546	1184	424	24	1251	6	11352	1137	**[year]**	**Lifespan**
x	1	1	4	4	1	2	4	1	3	3	4	3	4	2	3		**SE_Q**
158432029	23055227	14843060	22409875	0	2218247	0	0	0	27614105	27047178	225147	1409279	17096	6535877	4797928	**[m^2]**	**LU_C**
4	4	3	4	1	3	1	1	1	4	4	2	3	1	3	3		**LU_C_Q**
x	15	14	18	11	15	12	x	9	24	20	17	20	18	22	23		**SUM_Q**

HPPD_ID	Cap	r_vol	r_area	RRI_04	RRI_04_Q	RFI_04	RFI_04_Q	Res	Res_Q	PA_No	PA	PA_Q	MF	MF_Q	PE	PE_Q	SE	Lifespan	SE_Q	LU_C	LU_C_Q	SUM_Q
	[MW]	[million m³]	[million m²]	[%]		[%]		count		count	[m²]		count		[m3/year]		[t/year]	[year]		[m²]		
10395	44	5256	154	585	4	63	2	197701	4		0	1	5	2	15654905	4	930	14978620	1	167698206	4	22
10398	4	6	1	585	4	63	2	2209	2		0	1	4	2	55714	1	51645	285	3	614332	2	17
10402	14	102	45	0	1	21	1	7930	4		0	1	4	2	6460362	3	5	53448255	1	4474941	3	16
10410	360	89	25	252	4	80	4	1178	2		0	1	6	3	5389381	3	1396051	170	3	4483629	3	23
10412	312	47	18	252	4	80	4	2415	3		0	1	7	3	4466772	3	6855	18355	1	1799236	3	22
10413	420	8	14	252	4	80	4	65	1		0	1	7	3	3170109	3	x	x	x	113239	2	x
10414	205	187	100	253	4	50	2	54823	4		0	1	8	4	18716582	4	198401663	2	4	52326529	4	27
10421	648	243	66	252	4	80	4	3860	3		0	1	6	3	13847909	4	4910	131109	1	9963034	3	23
10429	47	x	x	56	3	47	1	x	x	x	x	x	x	x	x	x	x	x	x	x	x	x
10431	118	3095	52	107	3	6	1	1528	2		0	1	3	1	6828844	3	1165033	7040	2	705704	2	15
10452	160	363	19	56	3	47	1	453	2	2	29121714	4	4	2	2640784	3	192	5007800	1	58000	2	18
10459	60	8	5	56	3	47	1	295	1	2	8679651	4	2	1	729218	2	20	1035049	1	779519	2	15
10462	358	x	x	56	3	47	1	x	x	x	x	x	x	x	x	x	x	x	x	x	x	x
10469	300	58	1	107	3	6	1	136	1		0	1	4	2	163458	1	12794	12012	2	0	1	12
10474	520	361	6	107	3	6	1	1031	2		0	1	3	1	782921	2	316379	3022	2	187521	2	14
10479	7	12	1	585	4	63	2	2856	3		0	1	8	4	88405	1	1213807	26	4	873491	2	21

HPPD_ID	Cap	r_vol	r_area	RRI_04	RRI_04_Q	RFI_04	RFI_04_Q	Res	Res_Q	PA_No	PA	PA_Q	MF	MF_Q	PE	PE_Q	SE	Lifespan	SE_Q	LU_C	LU_C_Q	SUM_Q
	[MW]	[million m³]	[million m²]	[%]		[%]		count		count	[m²]		count		[m3/year]		[t/year]	[year]		[m²]		
10515	360	0	0	585	4	63	2	0	1	1	18121	3	9	4	1130	1	x	x	x	0	1	x
10526	45	9	0	585	4	63	2	182	1	1	241058	3	5	2	21905	1	7647	3082	2	0	1	16
10535	38	1	0	585	4	63	2	494	2		0	1	4	2	21926	1	96	14003	1	2377	1	14
10550	26	141	18	0	1	x	x	125	1		0	1	4	2	2166521	2	124	3027324	1	490797	2	x
10569	381	2274	166	94	3	67	3	5108	3		0	1	3	1	18470886	4	2103602	2865	2	5010206	3	20
10570	260	54	5	94	3	67	3	143	1		0	1	3	1	573004	1	41981	3439	2	341926	2	14
10571	330	3	1	94	3	67	3	55	1		0	1	4	2	109468	1	663993	11	4	0	1	16
10572	453	65	7	94	3	67	3	235	1		0	1	4	2	800569	2	155409	1107	3	0	1	16
10573	460	267	14	94	3	67	3	660	2		0	1	4	2	1607411	2	328424	2157	2	6696	1	16
10574	114	51	3	94	3	67	3	220	1		0	1	4	2	384928	1	24860	5437	2	20005	2	15
10575	120	35557	3202	94	3	67	3	16103	4	2	965594865	4	3	1	366089020	4	222	424049347	1	17345704	4	25
10576	203	47	1	36	2	0	1	116	1		0	1	3	1	145197	1	142937	868	3	0	1	11
10577	121	1151	64	36	2	0	1	2280	2		0	1	3	1	6843337	3	1810115	1685	2	12537037	3	16
10578	217	34	5	49	3	7	1	364	2		0	1	3	1	556315	1	273432	332	3	727849	2	14
10579	283	59	2	49	3	7	1	491	2		0	1	3	1	247541	1	968470	162	3	0	1	13
10581	403	4617	440	49	3	7	1	32827	4		0	1	3	1	46555523	4	9278378	1319	3	127897811	4	21

10670	10669	10653	10650	10649	10647	10642	10638	10637	10604	10602	10596	10594	10585	10584	10583		**HPPD_ID**
200	650	350	55	50	50	150	30	60	74	230	190	41	57	86	157	**[MW]**	**Cap**
5	65	1336	239	19	10	789	29	7	6785	520	16	198	3	41	138	**[million m^3]**	**r_vol**
2	25	64	10	2	5	14	2	0	295	37	1	8	0	5	10	**[million m^2]**	**r_area**
1066	1066	107	107	107	107	107	107	107	140	125	2	2	94	94	94	**[%]**	**RRI_04**
4	4	3	3	3	3	3	3	3	4	3	1	1	3	3	3		**RRI_04_Q**
60	60	6	6	6	6	6	6	6	x	50	13	13	67	67	67	**[%]**	**RFI_04**
2	2	1	1	1	1	1	1	1	x	2	1	1	3	3	3		**RFI_04_Q**
72	332	3629	0	0	1317	2368	312	39	5107	2604	150	1932	51	231	1902	**count**	**Res**
1	1	3	1	1	2	3	1	1	3	3	1	2	1	1	2		**Res_Q**
			1	1				1								**count**	**PA_No**
0	0	0	10534017	2046802	0	0	0	135334	0	0	0	0	0	0	0	**[m^2]**	**PA**
1	1	1	4	3	1	1	1	3	1	1	1	1	1	1	1		**PA_Q**
5	5	4	4	4	4	4	5	5	3	2	5	5	3	3	3	**count**	**MF**
2	2	2	2	2	2	2	2	2	1	1	2	2	1	1	1		**MF_Q**
336698	3616344	8832391	1261066	224603	710469	1697339	244592	51781	38161111	4556683	82785	880910	47089	576208	1111220	**[m3/year]**	**PE**
1	3	3	2	1	2	2	1	1	4	3	1	2	1	1	2		**PE_Q**
10560533	14	41701904	966877	113	1145611	2555225	584863	75968	6003028	14	10625369	549199	2069821	356	110153	**[t/year]**	**SE**
1	12452332	85	654	450798	23	818	130	256	2995	96293997	4	956	3	307679	3331	**[year]**	**Lifespan**
4	1	4	3	1	4	3	4	3	2	1	4	3	4	1	2		**SE_Q**
0	381097	53886044	0	0	4934560	1275210	768093	335364	25608759	0	8737	0	0	0	0	**[m^2]**	**LU_C**
1	2	4	1	1	3	2	2	2	4	1	1	1	1	1	1		**LU_C_Q**
16	16	21	17	13	18	17	15	16	x	15	12	13	15	12	15		**SUM_Q**

HPPD_ID	Cap	r_vol	r_area	RRI_04	RRI_04_Q	RFI_04	RFI_04_Q	Res	Res_Q	PA_No	PA	PA_Q	MF	MF_Q	PE	PE_Q	SE	Lifespan	SE_Q	LU_C	LU_C_Q	SUM_Q
	[MW]	[million m³]	[million m²]	[%]		[%]		count		count	[m²]		count		[m3/year]		[t/year]	[year]		[m²]		
10672	1245	247309	4728	1066	4	60	2	38424	4	2	4608230075	4	5	2	654496164	4	30150183	21737	1	330168092	4	25
10680	612	700	17	125	3	50	2	105	1	2	16608402	4	5	2	1871109	2	10	194015391	1	0	1	16
10722	320	83372	2626	509	4	36	1	51394	4	8	4382205770	4	5	2	364860007	4	25195161	8769	2	1211718480	4	25
10741	10	8	2	26	2	52	2	170	1	1	1034517	3	4	2	313033	1	17918026	1	4	541796	2	17
10743	100	1232	106	92	3	52	2	2085	2	1	851044	3	3	1	13613185	4	521005	6264	2	7055842	3	20
10744	14	9	4	15	1	19	1	126	1		0	1	4	2	487488	1	6069356	4	4	456622	2	13
10745	1	0	0	15	1	19	1	9	1		0	1	3	1	10206	1	155908	4	4	87473	2	12
10748	40	2953	122	15	1	19	1	2264	2	4	67845340	4	4	2	13882976	4	16	494676069	1	0	1	16
10754	330	257	14	92	3	52	2	200	1		0	1	3	1	1778971	2	297873	2282	2	0	1	13
10760	132	1417	87	27	2	61	2	939	2	1	89476967	4	6	3	11213106	4	1748637	2147	2	844096	2	21
10761	86	599	21	27	2	61	2	290	1	2	22684516	4	6	3	2712523	3	275298	5767	2	487	1	18
10762	108	1386	46	27	2	61	2	185	1	1	21574061	4	6	3	5916935	3	268765	13665	2	383657	2	19
10770	200	196	18	44	2	65	3	3457	3		0	1	4	2	2176764	2	2317786	224	3	7947420	3	19
10772	320	83372	2626	509	4	36	1	51394	4	8	4382205770	4	5	2	364860007	4	x	x	x	1211718480	4	x
10783	620	935	70	138	4	50	2	593	2	3	55703698	4	5	2	9813790	3	29923472	83	4	222954	2	23
10786	15	7	1	44	2	65	3	338	2		0	1	4	2	130347	1	1121	15458	1	28195	2	13

HPPD_ID	Cap	r_vol	r_area	RRI_04	RRI_04_Q	RFI_04	RFI_04_Q	Res	Res_Q	PA_No	PA	PA_Q	MF	MF_Q	PE	PE_Q	SE	Lifespan	SE_Q	LU_C	LU_C_Q	SUM_Q
	[MW]	[million m³]	[million m²]	[%]		[%]		count		count	[m²]		count		[m3/year]		[t/year]	[year]		[m²]		
10787	5	0	0	44	2	65	3	26	1	1	17042	3	3	1	964	1	313323	1	4	0	1	16
10793	160	1506	109	10	1	81	4	8348	4	2	29048485	4	11	4	13768004	4	185842	21469	1	18536099	4	26
10794	108	177	19	10	1	81	4	2110	2	2	4947310	4	11	4	2396095	2	6332758	74	4	3151148	3	24
10820	11	1959	294	103	3	6	1	7986	4	4	96461901	4	6	3	39935919	4	4265403	1217	3	71417679	4	26
10822	60	759	201	162	4	56	2	12625	4	1	32764363	4	4	2	26967145	4	26971417	75	4	1706711	3	27
10825	5	259	108	20	1	20	1	8109	4	2	38035998	4	4	2	15512754	4	19	36127970	1	4221473	3	20
10827	14	x	x	94	3	70	3	x	x	x	x	x	x	x	x	x	x	x	x	x	x	x
10832	90	68	5	103	3	6	1	609	2	2	2961365	3	7	3	513853	1	42138	4279	2	343970	2	17
10833	90	1486	261	103	3	6	1	48911	4	4	67185888	4	7	3	29323824	4	16944139	232	3	241172188	4	26
10847	280	2403	208	49	3	67	3	1484	2	1	14701895	4	9	4	22840478	4	541454	11761	2	206011716	4	26
10852	156	108	12	49	3	67	3	7979	4		0	1	9	4	1307808	2	28972411	10	4	7264134	3	24
10853	150	311	61	49	3	67	3	7421	3	3	35147975	4	9	4	6821144	3	530	1555186	1	39132797	4	25
10860	225	32	11	22	1	80	4	181	1		0	1	8	4	1143762	2	3569	23893	1	1880257	3	17
10871	50	403	100	8	1	65	3	46629	4		0	1	8	4	10816525	4	6138590	174	3	97999365	4	23
10876	87	229	95	39	2	50	2	7894	4		0	1	11	4	12525100	4	88	6906617	1	11751312	3	21
10877	43	413	146	94	3	70	3	7710	4		0	1	8	4	19855855	4	9610620	114	4	5785115	3	26

HPPD_ID	Cap	r_vol	r_area	RRI_04	RRI_04_Q	RFI_04	RFI_04_Q	Res	Res_Q	PA_No	PA	PA_Q	MF	MF_Q	PE	PE_Q	SE	Lifespan	SE_Q	LU_C	LU_C_Q	SUM_Q
	[MW]	[million m^3]	[million m^2]	[%]		[%]		count		count	[m^2]		count		[m3/year]		[t/year]	[year]		[m^2]		
10878	36	1479	188	94	3	70	3	6699	3	1	30149747	4	6	3	25802348	4	23	167759831	1	186745	2	23
10892	13	x	x	22	1	80	4	x	x	x	x	x	x	x	x	x	x	x	x	x	x	x
10895	100	189	18	22	1	80	4	2974	3	1	270785	3	6	3	2391767	2	707688	706	3	11238166	3	22
10899	2	3	1	22	1	80	4	289	1		0	1	6	3	105507	1	216736	40	4	587461	2	17
10901	67	244	16	22	1	80	4	2145	2		0	1	6	3	2133908	2	1283849	504	3	4583932	3	19
10906	27	751	89	22	1	80	4	10707	4		0	1	5	2	11126965	4	231	8625250	1	15391821	3	20
10907	23	381	28	22	1	80	4	2049	2		0	1	5	2	3649057	3	1433315	705	3	1172928	2	18
10908	15	56	13	22	1	80	4	2352	3		0	1	5	2	1635926	2	15913	9358	2	2949511	3	18
10909	40	1031	33	22	1	80	4	2276	2		0	1	5	2	4104656	3	780970	3497	2	1039164	2	17
10911	29	1818	128	22	1	80	4	9442	4		0	1	5	2	16481535	4	44700	107781	1	8783330	3	20
10912	21	1069	91	22	1	80	4	7762	4		0	1	5	2	11716274	4	134618	21040	1	6125639	3	20
10913	24	1462	64	22	1	80	4	5897	3		0	1	5	2	7995614	3	294985	13130	2	6662101	3	19
10914	14	231	16	22	1	80	4	3014	3		0	1	5	2	2049947	2	905225	675	3	5311244	3	19
10915	19	18	1	22	1	80	4	227	1		0	1	5	2	116597	1	9024	5321	2	448030	2	14
10916	84	600	14	22	1	80	4	2452	3	1	1098518	3	5	2	1671709	2	229057	6946	2	1303403	3	20
10917	81	95	7	22	1	80	4	2838	3		0	1	5	2	897100	2	594253	422	3	0	1	17

10986	10984	10980	10970	10968	10965	10962	10956	10944	10930	10926	10924	10922	10921	10920	10919		**HPPD_ID**
161	281	76	40	98	58	21	18	174	128	16	82	36	90	115	120	**[MW]**	**Cap**
3670	27089	229	89	349	503	843	122	1798	1990	25	1273	81	87	141	512	**[million m^3]**	**r_vol**
220	612	80	18	24	15	78	1	57	128	10	60	12	8	11	40	**[million m^2]**	**r_area**
958	958	958	22	22	958	22	22	22	84	22	22	22	22	22	22	**[%]**	**RRI_04**
4	4	4	1	1	4	1	1	1	3	1	1	1	1	1	1		**RRI_04_Q**
68	68	68	80	80	68	80	80	80	x	80	80	80	80	80	80	**[%]**	**RFI_04**
3	3	3	4	4	3	4	4	4	x	4	4	4	4	4	4		**RFI_04_Q**
5532	31603	9779	2341	5207	1586	2182	155	9298	3614	1080	2820	2456	1819	2167	5269	**count**	**Res**
3	4	4	3	3	2	2	1	4	3	2	3	3	2	2	3		**Res_Q**
2	4										1					**count**	**PA_No**
105422144	366265105	0	0	0	0	0	0	0	0	0	9351874	0	0	0	0	**[m^2]**	**PA**
4	4	1	1	1	1	1	1	1	1	1	4	1	1	1	1		**PA_Q**
3	4	4	5	6	5	5	6	5	5	5	5	6	6	6	6	**count**	**MF**
1	2	2	2	3	2	2	3	2	2	2	2	3	3	3	3		**MF_Q**
30374598	80572712	10148277	2395727	3145750	1967740	10071541	160926	7175296	17709249	1324146	7869937	1574333	1028968	1395101	5138845	**[m3/year]**	**PE**
4	4	3	2	3	2	3	1	3	4	2	3	2	2	2	3		**PE_Q**
11097962	833	1567817	475	379591	94848	13053	25041	1722702	8714395	7730440	1708387	1130038	825529	8175	222146	**[t/year]**	**SE**
876	86194849	387	498151	2436	14051	171132	12916	2765	605	9	1975	190	279	45850	6112	**[year]**	**Lifespan**
3	1	3	1	2	1	1	2	2	3	4	2	3	3	1	2		**SE_Q**
8730413	2421674	186578	983612	1156138	373198	1677	0	4038111	501971	0	2458221	4902767	5171439	6956140	19844808	**[m^2]**	**LU_C**
3	3	2	2	2	2	1	1	3	2	1	3	3	3	3	4		**LU_C_Q**
25	25	22	16	19	17	15	14	20	x	17	22	20	19	17	21		**SUM_Q**

11048	11047	11045	11042	11041	11039	11036	11029	11024	11023	11021	11012	11009	11002	10990	10989		HPPD_ID
26	123	18	70	30	50	4	25	518	150	9	48	48	100	90	72	[MW]	Cap
8713	98	307	860	1095	30	377	882	7652	144	0	36	107	1029	1070	806	[million m³]	r_vol
744	52	81	113	107	11	115	528	480	40	0	10	25	225	163	153	[million m²]	r_area
62	62	1226	7	958	958	30	62	22	22	22	22	22	30	30	30	[%]	RRI_04
3	3	4	1	4	4	2	3	1	1	1	1	1	2	2	2		RRI_04_Q
71	71	9	18	68	68	26	71	80	80	80	80	80	26	26	26	[%]	RFI_04
3	3	1	1	3	3	1	3	4	4	4	4	4	1	1	1		RFI_04_Q
5492	2974	21572	1216	1901	433	14701	23347	32879	2729	93	3925	3324	7304	6861	2261	count	Res
3	3	4	2	2	2	4	4	4	3	1	3	3	3	3	2		Res_Q
7	5	1	1										2	2	2	count	PA_No
2720576957	55096399	556508	357805	0	0	0	0	0	0	0	0	0	322811489	164964235	73494252	[m²]	PA
4	4	3	3	1	1	1	1	1	1	1	1	1	4	4	4		PA_Q
8	7	4	2	2	3	7	3	8	8	8	6	6	8	10	10	count	MF
4	3	2	1	1	1	3	1	4	4	4	3	3	4	4	4		MF_Q
124541269	9049881	13140229	17672070	15797011	1542745	17551501	106927676	54301816	4421484	10123	1063459	2735909	29503594	22027089	20985979	[m3/year]	PE
4	3	4	4	4	2	4	4	4	3	1	2	3	4	4	4		PE_Q
1907155	x	20784016	24744260	3327066	4975103	1470	x	859116	2858405	661	321397	3542357	8820319	x	4796843	[t/year]	SE
12107	x	39	92	872	16	679541	x	23603	134	1580	299	80	309	x	445	[year]	Lifespan
2	x	4	4	3	4	1	x	1	3	3	3	4	3	x	3		SE_Q
137668072	22172888	48423240	35844817	12146474	0	63012907	20145429	223325879	31973698	71661	7477506	11472678	841813	15148843	0	[m²]	LU_C
4	4	4	4	3	1	4	4	4	4	2	3	3	2	3	1		LU_C_Q
26	x	25	20	21	18	20	x	23	23	17	20	21	22	x	21		SUM_Q

HPPD_ID	Cap	r_vol	r_area	RRI_04	RRI_04_Q	RFI_04	RFI_04_Q	Res	Res_Q	PA_No	PA	PA_Q	MF	MF_Q	PE	PE_Q	SE	Lifespan	SE_Q	LU_C	LU_C_Q	SUM_Q
	[MW]	[million m³]	[million m²]	[%]		[%]		count		count	[m²]		count		[m3/year]		[t/year]	[year]		[m²]		
11051	1870	5005	1120	130	4	x	x	157118	4	4	201191338	4	9	4	150084219	4	94	140485611	1	131328366	4	x
11066	4	616	82	44	2	19	1	32604	4	3	13327781	4	3	1	15283112	4	214771	7600	2	37149586	4	22
11068	1	167	15	105	3	64	3	45573	4		0	1	5	2	2603476	3	41953	10575	2	14894933	3	21
11069	360	1035	63	105	3	64	3	10699	4	2	24307154	4	8	4	9620054	3	1476592	1858	2	616519	2	25
11072	20	33	0	46	2	49	2	91	1	1	257984	3	7	3	33918	1	233399	371	3	0	1	16
11073	1	1	6	46	2	49	2	70	1		0	1	8	4	809292	2	114802	12	4	20966	2	18
11074	1062	2614	499	46	2	49	2	238484	4	2	12962097	4	9	4	69068581	4	x	x	x	91128126	4	x
11076	2	0	0	105	3	64	3	12	1		0	1	7	3	1055	1	43	3130	2	0	1	15
11079	9	694	76	10	1	81	4	13183	4		0	1	7	3	9145866	3	x	x	x	17121900	4	x
11081	2192	4942	439	14	1	x	x	70870	4	3	112057721	4	9	4	47677854	4	15325810	855	3	17430549	4	x
11103	24	230	49	10	1	81	4	4859	3	1	13789792	4	8	4	6065310	3	5449805	112	4	38368269	4	27
11104	9	44	14	10	1	81	4	1738	2		0	1	8	4	1767357	2	109469	1053	3	4604150	3	20
11106	8	52	14	10	1	81	4	1550	2		0	1	8	4	1723633	2	997754	138	3	0	1	18
11110	6	19	5	39	2	50	2	436	2	1	273410	3	7	3	605187	2	1283658	39	4	0	1	19
11113	13	13	3	39	2	50	2	649	2	1	187340	3	6	3	468375	1	1049533	33	4	2142853	3	20
11118	2100	x	x	x	x	x	x	x	x	x	x	x	x	x	x	x	x	x	x	x	x	x

HPPD_ID	Cap	r_vol	r_area	RRI_04	RRI_04_Q	RFI_04	RFI_04_Q	Res	Res_Q	PA_No	PA	PA_Q	MF	MF_Q	PE	PE_Q	SE	Lifespan	SE_Q	LU_C	LU_C_Q	SUM_Q
	[MW]	[million m³]	[million m²]	[%]		[%]		count		count	[m²]		count		[m3/year]		[t/year]	[year]		[m²]		
11148	26	91	3	533	4	56	2	719	2	3	1692724	3	1	1	383897	1	21643	11103	2	550104	2	17
11149	12	11	1	533	4	56	2	406	2	1	320575	3	1	1	83723	1	6177298	5	4	312007	2	19
11152	18	1	0	533	4	56	2	35	1		0	1	1	1	14470	1	283349	12	4	0	1	15
11156	38	136	7	533	4	56	2	2756	3		0	1	1	1	777867	2	1404301	256	3	505590	2	18
11157	30	983	39	533	4	56	2	1539	2	1	3340999	3	1	1	4449079	3	1124501	2316	2	5457411	3	20
11158	63	1003	38	533	4	56	2	120890	4	1	27761625	4	1	1	4290385	3	79	33860652	1	22073197	4	23
11174	30	1467	36	398	4	65	3	21628	4		0	1	1	1	3865234	3	80	48500743	1	26437227	4	21
11175	10	67	12	398	4	65	3	3565	3		0	1	1	1	1259695	2	357669	495	3	3977637	3	20
11178	12	1383	35	109	3	81	4	801	2	2	30640554	4	1	1	4048068	3	1386709	2644	2	7545957	3	22
11185	238	24194	959	92	3	52	2	13120	4	5	629373844	4	5	2	132110856	4	18136723	3535	2	43166557	4	25
11186	160	6782	203	107	3	6	1	45223	4		0	1	5	2	25897728	4	713965	25172	1	108468647	4	20
11187	150	2406	103	107	3	6	1	29626	4		0	1	4	2	13106717	4	1966688	3242	2	37997659	4	21
11191	480	2	0	94	3	67	3	27	1		0	1	2	1	38753	1	x	x	x	0	1	x
11193	147	205	3	27	2	61	2	4425	3		0	1	6	3	329407	1	4922776	110	4	917657	2	18
11194	287	313	4	27	2	61	2	5367	3		0	1	6	3	443152	1	2673	310179	1	1570194	3	16
11196	17	13	3	8	1	65	3	322	1	1	8307	3	7	3	274459	1	2314401	15	4	2934287	3	19

HPPD_ID	Cap	r_vol	r_area	RRI_04	RRI_04_Q	RFI_04	RFI_04_Q	Res	Res_Q	PA_No	PA	PA_Q	MF	MF_Q	PE	PE_Q	SE	Lifespan	SE_Q	LU_C	LU_C_Q	SUM_Q
	[MW]	[million m³]	[million m²]	[%]		[%]		count		count	[m²]		count		[m3/year]		[t/year]	[year]		[m²]		
11197	20	269	51	8	1	65	3	15172	4		0	1	7	3	5551890	3	468713	1518	3	55173850	4	22
11198	51	x	x	8	1	65	3	x	x	x	x	x	x	x	x	x	x	x	x	x	x	x
11202	20	407	31	585	4	63	2	18164	4		0	1	9	4	3919485	3	194326	5551	2	17810819	4	24
11226	380	291	19	212	4	75	3	4949	3		0	1	3	1	2890819	3	83622263	9	4	4922066	3	22

Table S3: Proposed hydropower plants of Type *Reservoir*. Each hydropower plant (HPP) can be identified with the respective HPPD_ID from the Renewable Power Plant Database (RePP) Africa (Peters et al., 2023). Rankings including all indicators for the entire continent (Ranking_all continent), and the major basins Congo, Niger, Nile, Volta, Zambezi (Ranking_all basin); rankings leaving out one indicator (ex_...) at a time (River Regulation (RRI), River Fragmentation (RFI), Resettlement (Res), Protected Areas (PA), Megafauna (MF), Potential Evaporation (PE), Sediment Entrapment (SE), and Land Use Change Cropland (LU_C); and rankings for different indicator compositions with A including PA, Res, and PU; B including MF, and SE; and C including PE, RFI, and RRI. Quartile values are indicated with 1 (low impact, $\leq 1^{st}$ quartile), 2 (moderate impact, $>1^{st}$ to $\leq 2^{nd}$ quartile), 3 (heavy impact, $>2^{nd}$ to $\leq 3^{rd}$ quartile), and 4 (severe impact, $>3^{rd}$ quartile).

HPPD_ID	Ranking_all continent	HYBAS_ID	Basin name	Ranking_all basin	Ex_RRI	Ex_RFI	Ex_Res	Ex_PA	Ex_MF	Ex_PE	Ex_SE	Ex_LU_C	Ranking A	Ranking B
10007	2	1030020050			2	1	2	2	1	2	1	2	3	1
10015	4	1030020050			4	4	4	4	4	4	4	4	3	4
10016	2	1030020050			2	2	3	3	2	2	3	2	1	3
10018	3	1030020050			3	3	3	3	3	3	3	3	3	3
10019	2	1030020050			2	1	2	2	1	2	1	2	3	1
10020	2	1030020050			2	2	3	3	2	3	2	3	3	2
10021	2	1030020050			2	2	3	2	2	2	1	2	3	1
10022	1	1030040190			1	1	1	1	1	1	1	1	1	1
10025	2	1030022420	Niger	1	2	3	3	3	2	2	3	3	1	3
10027	3	1030022420	Niger	3	4	4	3	4	3	3	3	3	2	4

HPPD_ID	Ranking_all continent	HYBAS_ID	Basin name	Ranking_all basin	Ex_RRI	Ex_RFI	Ex_Res	Ex_PA	Ex_MF	Ex_PE	Ex_SE	Ex_LU_C	Ranking A	Ranking B
10028	3	1030022420	Niger	3	4	4	3	3	3	3	4	4	1	4
10033	3	1030020050			3	3	3	3	3	3	3	4	3	3
10034	3	1030020050			3	3	3	3	3	3	4	3	1	4
10035	3	1030020040	Congo	4	3	3	3	2	3	2	3	3	1	3
10036	1	1030020050			2	1	1	1	1	1	1	1	1	3
10037	1	1030020040	Congo	2	1	1	1	1	1	1	1	1	1	1
10038	2	1030020040	Congo	3	3	2	2	3	2	2	2	3	2	3
10043	2	1030020040	Congo	2	2	3	2	2	1	2	1	2	1	3
10047	4	1030020050			4	4	4	3	4	4	4	4	1	4
10049	4	1030020050			4	4	3	3	3	3	4	3	1	4
10093	2	1030020040	Congo	2	2	3	2	2	2	1	3	1	1	4
10145	3	1030034260	Nile	2	2	3	3	2	2	3	2	3	4	2
10167	2	1030020050			2	3	2	1	1	2	3	2	1	4
10174	1	1030020050			1	1	1	1	1	1	1	1	1	1
10175	1	1030020050			1	1	1	1	1	1	1	1	1	1
10176	1	1030020050			2	2	2	1	1	1	2	1	1	3
10185	2	1030020040	Congo	3	2	3	2	3	2	3	3	2	1	3
10204	2	1030020040	Congo	2	2	2	1	2	2	2	3	2	1	3
10211	4	1030034260	Nile	4	4	4	4	4	4	4	4	4	4	3
10212	4	1030034260	Nile	3	3	3	3	4	4	4	4	4	4	3

HPPD_ID	Ranking_all continent	HYBAS_ID	Basin name	Ranking_all basin	Ex_RRI	Ex_RFI	Ex_Res	Ex_PA	Ex_MF	Ex_PE	Ex_SE	Ex_LU_C	Ranking A	Ranking B
10213	3	10300 40260			2	3	2	3	3	3	3	3	4	2
10214	2	10300 08100			2	2	2	1	2	1	1	2	1	2
10215	3	10300 34260	Nile	2	2	3	3	3	3	3	3	3	4	2
10232	2	10300 34260	Nile	2	1	1	2	2	2	2	2	1	3	1
10233	3	10300 34260	Nile	3	3	3	3	4	4	4	3	4	4	2
10234	1	10300 34260	Nile	1	1	1	1	2	2	2	1	2	4	1
10235	2	10300 34260	Nile	2	2	1	2	2	3	3	2	2	4	1
10238	2	10300 40260			2	2	2	3	3	3	2	3	4	1
10239	4	10300 40260			4	4	4	4	4	4	4	4	3	4
10241	4	10300 34260	Nile	4	4	4	4	4	4	4	4	4	3	4
10243	1	10300 34260	Nile	1	1	1	1	1	1	1	1	1	1	1
10245	1	10300 34260	Nile	1	2	2	1	2	1	1	1	2	1	2
10246	1	10300 34260	Nile	1	1	1	1	1	1	1	1	1	1	1
10247	1	10300 34260	Nile	1	1	1	1	1	1	1	1	1	1	2
10248	4	10300 40260			3	4	4	3	4	4	3	4	4	3
10249	1	10300 08100			1	1	1	1	1	1	1	1	3	1
10250	1	10300 08100			1	1	1	2	2	1	1	1	2	1
10280	3	10300 08110			4	4	3	3	4	3	4	3	1	4
10281	4	10300 34260	Nile	3	3	4	4	3	4	4	4	3	3	3
10282	2	10300 34260	Nile	2	1	2	2	1	2	2	2	2	3	1

10402	10398	10395	10358	10357	10356	10348	10343	10342	10322	10314	10311	10309	10301	10300	10299	10293	10292	10290	10283	HPPD_ID
1	2	3	1	1	2	1	1	1	1	4	3	2	3	2	3	4	2	4	4	**Ranking_all continent**
1030034260	1030034260	1030034260	1030035180	1030035180	1030035180	1030035180	1030035180	1030035180	1030035180	1030008110	1030008110	1030008110	1030040260	1030040260	1030040260	1030034260	1030034260	1030034260	1030034260	**HYBAS_ID**
Nile	Nile	Nile														Nile	Nile	Nile	Nile	**Basin name**
1	2	3														4	2	3	3	**Ranking_all basin**
2	1	3	2	1	2	1	2	1	1	4	2	2	2	2	3	4	1	3	3	**Ex_RRI**
2	2	4	1	1	3	1	1	1	1	4	3	2	3	2	3	4	2	4	4	**Ex_RFI**
1	2	3	1	1	2	1	1	1	1	4	3	2	3	3	3	4	2	3	4	**Ex_Res**
2	2	4	1	1	2	1	1	1	1	3	3	2	3	2	3	3	2	4	3	**Ex_PA**
1	2	3	1	1	3	1	1	1	1	4	3	2	3	3	4	4	2	3	4	**Ex_MF**
1	2	3	1	1	2	1	1	1	1	4	3	2	3	3	4	4	2	4	3	**Ex_PE**
2	1	4	1	1	1	1	1	1	1	4	3	1	3	1	4	4	1	4	4	**Ex_SE**
1	2	3	1	1	1	1	1	1	1	4	2	2	3	3	3	4	2	3	3	**Ex_LU_C**
1	3	2	1	1	1	1	1	1	1	3	3	3	4	4	3	3	4	3	2	**Ranking A**
3	1	4	3	2	3	1	2	1	1	4	3	1	2	1	3	4	1	3	4	**Ranking B**

10576	10575	10574	10573	10572	10571	10570	10569	10535	10526	10479	10474	10469	10459	10452	10431	10421	10414	10412	10410	**HPPD_ID**
1	4	1	1	1	1	1	3	1	1	3	1	1	1	2	1	4	4	3	4	**Ranking_all continent**
1030018110	1030018110	1030018110	1030018110	1030018110	1030018110	1030018110	1030018110	1030034260	1030034260	1030034260	1030011660	1030011660	1030008110	1030008110	1030011660	1030034260	1030034260	1030034260	1030034260	**HYBAS_ID**
								Nile	Nile	Nile	Zambezi	Zambezi			Zambezi	Nile	Nile	Nile	Nile	**Basin name**
								1	1	2	1	1			2	3	4	3	3	**Ranking_all basin**
1	4	1	1	1	1	1	2	1	1	2	1	1	1	2	1	3	4	3	3	**Ex_RRI**
1	4	1	1	1	1	1	3	1	1	3	1	1	1	3	1	3	4	3	3	**Ex_RFI**
1	4	1	1	2	2	1	3	1	2	3	1	1	1	2	1	4	4	3	4	**Ex_Res**
1	3	1	2	2	2	1	3	1	1	3	1	1	1	1	1	4	4	4	4	**Ex_PA**
1	4	1	1	1	1	1	3	1	1	3	1	1	1	2	1	3	4	3	3	**Ex_MF**
1	4	1	1	1	2	1	2	1	2	4	1	1	1	2	1	3	4	3	4	**Ex_PE**
1	4	1	1	1	1	1	3	1	1	3	1	1	1	3	1	4	4	4	4	**Ex_SE**
1	4	1	2	2	2	1	3	1	2	3	1	1	1	2	1	4	4	3	4	**Ex_LU_C**
1	2	3	3	3	4	3	3	2	3	4	1	1	1	1	1	3	4	3	4	**Ranking A**
1	4	1	1	1	1	1	3	1	1	2	1	1	2	3	1	3	4	3	3	**Ranking B**

10672	10670	10669	10653	10650	10649	10647	10642	10638	10637	10602	10596	10594	10585	10584	10583	10581	10579	10578	10577	**HPPD_ID**
4	1	1	3	2	1	2	2	1	1	1	1	1	1	1	1	3	1	1	1	**Ranking_all continent**
10300 11660	10300 11660	10300 11660	10300 11660	10300 11660	10300 11660	10300 11660	10300 11660	10300 11660	10300 11660	10300 18110	10300 18110	10300 18110	10300 18110	10300 18110	10300 18110	10300 18110	10300 18110	10300 18110	10300 18110	**HYBAS_ID**
Zamb ezi	Zamb ezi	Zamb ezi	Zamb ezi	Zamb ezi	Zamb ezi	Zamb ezi	Zamb ezi	Zamb ezi	Zamb ezi											**Basin name**
4	2	2	4	3	1	3	3	2	2											**Ranking_all basin**
4	1	1	3	2	1	2	2	1	1	1	1	1	1	1	1	3	1	1	1	**Ex_RRI**
4	1	1	4	2	1	3	2	1	2	1	1	1	1	1	1	4	1	1	1	**Ex_RFI**
4	2	2	3	2	1	2	1	1	2	1	1	1	1	1	1	3	1	1	1	**Ex_Res**
4	2	2	3	1	1	2	2	1	1	1	1	1	1	1	1	3	1	1	1	**Ex_PA**
4	1	1	3	2	1	2	2	1	1	1	1	1	1	1	1	3	1	1	1	**Ex_MF**
4	2	1	3	2	1	2	2	1	2	1	1	1	1	1	1	3	1	1	1	**Ex_PE**
4	1	2	3	1	1	1	1	1	1	1	1	1	1	1	1	3	1	1	1	**Ex_SE**
4	2	1	3	2	1	2	2	1	1	1	1	1	1	1	1	3	1	1	1	**Ex_LU_C**
2	4	2	3	2	1	3	2	3	2	1	1	1	4	2	3	2	2	2	1	**Ranking A**
4	1	1	3	2	1	2	2	1	1	1	1	1	1	1	1	3	1	1	2	**Ranking B**

HPPD_ID	Ranking_all continent	HYBAS_ID	Basin name	Ranking_all basin	Ex_RRI	Ex_RFI	Ex_Res	Ex_PA	Ex_MF	Ex_PE	Ex_SE	Ex_LU_C	Ranking A	Ranking B
10680	1	1030018110			1	1	2	1	1	1	2	2	1	2
10722	4	1030011660	Zambezi	4	4	4	4	4	4	4	4	4	2	4
10741	2	1030011660	Zambezi	3	2	2	2	1	2	2	1	2	3	1
10743	3	1030011660	Zambezi	3	2	3	3	2	3	2	3	3	2	3
10744	1	1030011660	Zambezi	1	1	1	1	1	1	1	1	1	1	1
10745	1	1030011660	Zambezi	1	1	1	1	1	1	1	1	1	1	1
10748	1	1030011660	Zambezi	2	2	2	1	1	1	1	2	2	1	3
10754	1	1030011660	Zambezi	1	1	1	1	1	1	1	1	1	2	1
10760	3	1030020040	Congo	4	3	3	3	2	3	3	3	3	1	4
10761	2	1030020040	Congo	2	2	2	3	1	2	2	2	3	1	3
10762	2	1030020040	Congo	3	2	3	3	2	2	2	3	3	1	3
10770	2	1030011670			2	2	2	3	3	3	2	2	3	2
10783	4	1030011660	Zambezi	4	3	4	4	3	4	4	3	4	4	3
10786	1	1030011670			1	1	1	1	1	1	1	1	1	1
10787	1	1030011670			2	1	2	1	2	2	1	2	3	1
10793	4	1030022430			4	4	4	4	4	4	4	4	1	4
10794	4	1030022430			4	4	4	3	3	4	4	4	3	4
10820	4	1030023310			4	4	4	4	4	4	4	4	2	4
10822	4	1030023300	Volta	4	4	4	4	4	4	4	4	4	4	4
10825	3	1030023300	Volta	1	3	3	2	2	3	2	3	3	1	4

HPPD_ID	Ranking_all continent	HYBAS_ID	Basin name	Ranking_all basin	Ex_RRI	Ex_RFI	Ex_Res	Ex_PA	Ex_MF	Ex_PE	Ex_SE	Ex_LU_C	Ranking A	Ranking B
10832	2	10300 23310			2	2	2	1	1	2	2	2	1	2
10833	4	10300 23310			4	4	4	4	4	4	4	4	2	4
10847	4	10300 23310			4	4	4	4	4	4	4	4	3	4
10852	4	10300 23310			4	4	4	4	3	4	4	4	4	3
10853	4	10300 23310			4	4	4	4	4	4	4	4	2	4
10860	2	10300 23310			2	1	2	2	1	2	2	1	1	2
10871	4	10300 23310			4	4	4	4	3	4	4	4	2	4
10876	3	10300 23300	Volta	2	3	3	3	3	3	3	4	3	1	4
10877	4	10300 23300	Volta	4	4	4	4	4	4	4	4	4	4	4
10878	4	10300 23300	Volta	3	4	4	4	3	3	3	4	4	2	4
10895	3	10300 23310			4	3	3	3	3	4	3	3	3	3
10899	2	10300 23310			2	1	2	2	1	2	1	2	3	1
10901	2	10300 23310			3	2	3	3	2	3	2	2	3	2
10906	3	10300 23310			3	2	2	3	3	2	3	3	1	3
10907	2	10300 23310			2	1	2	2	2	2	2	2	3	2
10908	2	10300 23310			2	1	2	2	2	2	2	2	2	2
10909	2	10300 23310			2	1	2	2	2	1	2	2	2	2
10911	3	10300 23310			3	2	2	3	3	2	3	3	1	3
10912	3	10300 23310			3	2	2	3	3	2	3	3	1	3
10913	2	10300 23310			3	2	2	3	3	2	3	2	2	3

HPPD_ID	Ranking_all continent	HYBAS_ID	Basin name	Ranking_all basin	Ex_RRI	Ex_RFI	Ex_Res	Ex_PA	Ex_MF	Ex_PE	Ex_SE	Ex_LU_C	Ranking A	Ranking B
10914	2	1030023310			3	2	2	3	3	3	2	2	3	2
10915	1	1030023310			1	1	1	1	1	1	1	1	2	1
10916	3	1030023310			3	2	3	2	3	3	3	3	2	3
10917	2	1030023310			2	1	1	2	2	2	1	2	3	1
10919	3	1030023310			4	3	3	3	3	3	3	3	2	3
10920	2	1030023310			2	1	2	2	1	2	2	1	1	2
10921	2	1030023310			3	2	3	3	2	3	2	2	3	2
10922	3	1030023310			3	2	3	3	3	3	3	3	3	3
10924	3	1030023310			4	3	3	3	3	3	4	3	2	4
10926	2	1030023310			2	1	2	2	2	2	1	2	3	1
10944	3	1030023310			3	2	2	3	3	3	3	3	2	3
10956	1	1030023310			1	1	1	1	1	1	1	1	2	1
10962	1	1030023310			2	1	1	1	1	1	1	1	1	1
10965	2	1030027410			1	1	2	2	2	2	2	2	3	1
10968	2	1030023310			3	2	2	3	2	2	3	3	2	3
10970	1	1030023310			2	1	1	2	1	1	2	1	1	2
10980	3	1030027410			3	3	3	4	3	3	3	4	4	3
10984	4	1030027410			4	4	4	4	4	4	4	4	3	4
10986	4	1030027410			4	4	4	4	4	4	4	4	4	4
10989	3	1030022420	Niger	2	3	4	3	2	3	3	3	4	1	4

HPPD_ID	Ranking_all continent	HYBAS_ID	Basin name	Ranking_all basin	Ex_RRI	Ex_RFI	Ex_Res	Ex_PA	Ex_MF	Ex_PE	Ex_SE	Ex_LU_C	Ranking A	Ranking B
11002	4	10300 22420	Niger	4	4	4	4	3	3	3	4	4	1	4
11009	3	10300 23310			4	3	3	4	3	3	3	3	3	3
11012	3	10300 23310			3	2	3	3	3	3	3	3	3	3
11021	2	10300 23310			2	1	2	2	1	2	1	2	3	1
11023	4	10300 23310			4	3	4	4	3	4	4	3	3	4
11024	4	10300 23310			4	3	3	4	3	3	4	3	1	4
11036	3	10300 22420	Niger	2	3	3	2	3	3	2	3	2	1	4
11039	2	10300 27410			2	2	2	2	3	2	1	3	4	1
11041	3	10300 27410			2	3	3	3	3	3	3	3	4	2
11042	3	10300 27410			3	3	3	2	3	2	2	2	1	3
11045	4	10300 27410			4	4	4	4	4	4	4	4	3	4
11048	4	10300 22420	Niger	4	4	4	4	4	4	4	4	4	3	4
11066	3	10300 22420	Niger	3	4	4	3	3	4	3	4	3	1	4
11068	3	10300 22420	Niger	2	3	3	3	3	3	3	3	3	3	3
11069	4	10300 22420	Niger	4	4	4	4	4	4	4	4	4	3	4
11072	1	10300 22420	Niger	1	2	1	2	1	1	2	1	2	2	1
11073	2	10300 22420	Niger	1	2	2	3	2	1	2	1	2	3	2
11076	1	10300 22420	Niger	1	1	1	1	1	1	1	1	1	3	1
11103	4	10300 22430			4	4	4	4	4	4	4	4	3	4
11104	3	10300 22430			3	2	3	3	2	3	3	3	3	3

HPPD_ID	Ranking_all continent	HYBAS_ID	Basin name	Ranking_all basin	Ex_RRI	Ex_RFI	Ex_Res	Ex_PA	Ex_MF	Ex_PE	Ex_SE	Ex_LU_C	Ranking A	Ranking B
11106	2	10300<br>22430			2	1	2	2	1	2	2	3	3	2
11110	2	10300<br>23300	Volta	1	2	3	3	2	2	3	2	3	3	2
11113	3	10300<br>23300	Volta	1	3	3	3	2	3	3	2	3	3	3
11148	2	10300<br>27430			1	2	2	1	2	2	2	2	3	1
11149	2	10300<br>27430			2	3	3	2	3	3	2	3	4	1
11152	1	10300<br>27430			1	1	1	1	1	1	1	1	4	1
11156	2	10300<br>27430			2	2	2	2	3	2	2	2	3	1
11157	3	10300<br>27430			2	3	3	2	3	3	3	3	3	3
11158	4	10300<br>27430			3	4	3	3	4	4	4	3	2	4
11174	3	10300<br>27430			2	3	3	3	3	3	4	3	3	3
11175	3	10300<br>27430			2	3	3	3	3	3	3	3	4	2
11178	3	10300<br>29810			3	3	4	3	4	3	4	3	3	3
11185	4	10300<br>11660	Zamb<br>ezi	4	4	4	4	4	4	4	4	4	2	4
11186	3	10300<br>11660	Zamb<br>ezi	3	2	3	2	3	3	2	3	2	1	4
11187	3	10300<br>11660	Zamb<br>ezi	4	3	4	3	3	3	3	3	3	1	4
11193	2	10300<br>20040	Cong<br>o	2	2	2	2	2	2	3	1	2	3	2
11194	1	10300<br>20040	Cong<br>o	2	2	1	1	2	1	2	2	1	1	2
11196	2	10300<br>23310			3	2	3	2	2	3	2	2	3	2
11197	3	10300<br>23310			4	3	3	4	3	3	3	3	2	4
11202	4	10300<br>34260	Nile	4	4	4	4	4	3	4	4	4	3	4

HPPD_ID	Ranking_all continent	HYBAS_ID	Basin name	Ranking_all basin	Ex_RRI	Ex_RFI	Ex_Res	Ex_PA	Ex_MF	Ex_PE	Ex_SE	Ex_LU_C	Ranking A	Ranking B
11226	3	10300 34260	Nile	3	3	3	3	4	4	3	3	3	4	2

Table S4: Proposed hydropower plants of Type *Reservoir* and *unknown*. Each hydropower plant (HPP) can be identified with the respective HPPD_ID from the Renewable Power Plant Database (RePP) Africa (Peters et al., 2023). Calculation of impact per indicator and resulting quartile (Q) for River Regulation (RRI, HydroBASIN level 04), River Fragmentation (RFI, HydroBASIN level 04), Resettlement (Res), Protected Area (PA), Megafauna (MF), Potential Evaporation (PE), Sediment Entrapment (SE) and resulting Lifespan, and Land Use Change for Cropland for Cropland (LU_C). Quartile values are indicated with 1 (low impact, ≤1st quartile), 2 (moderate impact, >1st to ≤2nd quartile), 3 (heavy impact, >2nd to ≤3rd quartile), and 4 (severe impact, >3rd quartile).

HPPD_ID	Cap	r_vol	r_area	RRI_04	RRI_04_Q	RFI_04	RFI_04_Q	Res	Res_Q	PA_No	PA	PA_Q	MF	MF_Q	PE	PE_Q	SE	Lifespan	SE_Q	LU_C	LU_C_Q	SUM_Q
	[MW]	[million m^3]	[million m^2]	[%]		[%]		count		count	[m^2]		count		[m^3/year]		[t/year]	[year]		[m^2]		
10006	1000	x	x	39	2	78	3	x	x	x	x	x	x	x	x	x	x	x	x	x	x	x
10007	470	72	5	39	2	78	3	165	1		0	1	8	3	542766	2	21871669	9	4	0	1	18
10008	200	71	10	14	1	49	2	2151	3		0	1	10	4	1062120	2	14413530	13	4	0	1	18
10010	6	72	18	10	1	43	1	1403	3		0	1	7	3	1960770	3	x	x	x	2782449	3	x
10015	1000	112	34	39	2	78	3	37258	4	1	17171062	4	9	4	3589321	3	1127706	264	3	630680	2	25
10016	250	1217	64	39	2	78	3	416	2		0	1	9	4	6777019	4	30	106277653	1	4745438	3	20
10017	365	135	18	1	1	45	2	796	2		0	1	8	3	1814803	3	2408528	148	3	2269177	3	19
10018	1800	1327	67	39	2	78	3	1820	3		0	1	9	4	7272455	4	6498931	541	3	3964642	3	23
10019	640	0	0	39	2	78	3	1	1		0	1	9	4	1852	1	50141	5	4	7349	2	18
10020	940	30	9	39	2	78	3	92	1		0	1	9	4	1006429	2	19010349	4	4	107594	2	19
10021	5	57	21	39	2	78	3	114	1		0	1	7	3	2349331	3	5542075	27	4	19504	2	19
10022	33	36	5	2	1	23	1	291	1		0	1	6	2	654628	2	4635378	21	4	0	1	13

HPPD_ID	Cap	r_vol	r_area	RRI_04	RRI_04_Q	RFI_04	RFI_04_Q	Res	Res_Q	PA_No	PA	PA_Q	MF	MF_Q	PE	PE_Q	SE	Lifespan	SE_Q	LU_C	LU_C_Q	SUM_Q
	[MW]	[million m³]	[million m²]	[%]		[%]		count		count	[m²]		count		[m³/year]		[t/year]	[year]		[m²]		
10024	66	25	1	49	3	72	3	122	1		0	1	9	4	164761	1	170848	383	3	0	1	17
10025	67	2513	245	49	3	72	3	1899	3		0	1	10	4	29551980	4	1152088	5781	2	99076	2	22
10027	72	1993	53	49	3	72	3	23662	4		0	1	10	4	5824247	3	3708997	1424	2	4526014	3	23
10028	485	3604	68	49	3	72	3	5368	3	1	6870267	4	10	4	7820842	4	6020412	1586	2	411023	2	25
10031	5	1	0	0	1	x	x	23	1		0	1	8	3	19013	1	218392	11	4	182993	2	x
10033	1200	1577	85	39	2	78	3	10456	4		0	1	8	3	9431898	4	7186173	581	3	0	1	22
10034	400	1579	108	39	2	78	3	7181	4		0	1	8	3	12341525	4	20	211904235	1	2233150	3	22
10035	600	3251	111	10	1	43	1	1517	3	1	45325338	4	6	2	10663587	4	3347491	2574	2	0	1	18
10036	1	99	15	1	1	45	2	18764	4		0	1	5	2	1483545	2	10507	24861	1	12832623	4	17
10037	40	90	25	0	1	52	2	204	1		0	1	5	2	2890490	3	10683518	22	4	0	1	15
10038	180	492	65	10	1	43	1	2375	3		0	1	9	4	6746656	4	7318510	178	3	1084786	3	19
10040	20	x	x	2	1	23	1	x	x	x	x	x	x	x	x	x	x	x	x	x	x	x
10042	2	156	34	1	1	x	x	453	2		0	1	7	3	4134366	3	434320	951	3	0	1	x
10043	300	131	62	0	1	52	2	1081	2		0	1	9	4	6978078	4	69263736	5	4	295532	2	20
10047	1000	12646	833	45	3	51	2	8437	4	2	246482932	4	7	3	82514112	4	844133	39699	1	79625983	4	24
10049	1000	16535	996	45	3	51	2	11907	4	3	234331146	4	7	3	98590496	4	2204964	19873	1	98895767	4	25
10051	650	24	9	9	1	51	2	812	2		0	1	11	4	909205	2	216	297076	1	52745	2	15

HPPD_ID	Cap	r_vol	r_area	RRI_04	RRI_04_Q	RFI_04	RFI_04_Q	Res	Res_Q	PA_No	PA	PA_Q	MF	MF_Q	PE	PE_Q	SE	Lifespan	SE_Q	LU_C	LU_C_Q	SUM_Q
	[MW]	[million m^3]	[million m^2]	[%]		[%]		count		count	[m^2]		count		[m^3/year]		[t/year]	[year]		[m^2]		
10052	1968	11	11	9	1	51	2	384	2		0	1	10	4	1173768	2	345476122	x	x	0	1	x
10053	950	x	x	9	1	51	2	x	x	x	x	x	x	x	x	x	x	x	x	x	x	x
10054	800	x	x	9	1	51	2	x	x	x	x	x	x	x	x	x	x	x	x	x	x	x
10058	28	1659	120	11	1	3	1	590	2	1	43196533	4	8	3	12439603	4	906186	4852	2	42435446	4	22
10059	9	9	2	11	1	3	1	61	1		0	1	11	4	264707	1	198068	117	3	234688	2	14
10060	14	18	12	11	1	3	1	131	1		0	1	10	4	1293239	2	32	1504822	1	1880836	3	14
10063	24	310	45	11	1	3	1	237	1		0	1	7	3	4562171	3	354715	2315	2	6677646	4	16
10064	9	338	74	11	1	3	1	1007	2		0	1	7	3	7683670	4	1385124	646	3	9487781	4	19
10065	5	36	11	10	1	43	1	182	1	1	7900488	4	7	3	1067138	2	49	1954637	1	2383988	3	16
10066	7	158	24	10	1	43	1	407	2	1	25736067	4	7	3	2371894	3	64	6486745	1	2786017	3	18
10069	8	72	21	10	1	43	1	393	2	1	22768626	4	7	3	2091530	3	424851	448	3	8715202	4	21
10070	8	3	1	10	1	43	1	4	1		0	1	7	3	121703	1	333447	26	4	0	1	13
10071	2	0	0	10	1	43	1	3	1	1	94470	3	7	3	7697	1	301317	1	4	77423	2	16
10074	4	254	87	10	1	43	1	63	1	3	16518169	4	10	4	8442774	4	1758711	383	3	9688063	4	22
10076	3	20	4	10	1	43	1	58	1		0	1	7	3	391952	2	104	498758	1	1907153	3	13
10084	3	38	3	10	1	43	1	12	1		0	1	10	4	333151	1	39522	2567	2	1118210	3	14
10090	50	38	10	45	3	51	2	146	1	2	2965376	4	6	2	939378	2	9282265	11	4	3636445	3	21

HPPD_ID	Cap	r_vol	r_area	RRI_04	RRI_04_Q	RFI_04	RFI_04_Q	Res	Res_Q	PA_No	PA	PA_Q	MF	MF_Q	PE	PE_Q	SE	Lifespan	SE_Q	LU_C	LU_C_Q	SUM_Q
	[MW]	[million m³]	[million m²]	[%]		[%]		count		count	[m²]		count		[m³/year]		[t/year]	[year]		[m²]		
10091	60	23	8	9	1	51	2	933	2		0	1	11	4	867355	2	223	268750	1	3715	2	15
10093	150	12089	371	11	1	3	1	3616	3		0	1	7	3	37830034	4	330	96956047	1	52814291	4	18
10094	18	1	0	11	1	3	1	13	1		0	1	7	3	9458	1	x	x	x	1373	2	x
10095	600	834	152	10	1	43	1	1031	2	5	103346520	4	8	3	15076837	4	x	x	x	560918	2	x
10103	7180	704	22	9	1	51	2	2890	3		0	1	11	4	2102755	3	90	20765561	1	15769513	4	19
10104	6970	704	22	9	1	51	2	2890	3		0	1	11	4	2102755	3	x	x	x	15769513	4	x
10105	6684	704	22	9	1	51	2	2890	3		0	1	11	4	2102755	3	x	x	x	15769513	4	x
10106	6706	704	22	9	1	51	2	2890	3		0	1	11	4	2102755	3	x	x	x	15769513	4	x
10107	4800	704	22	9	1	51	2	2890	3		0	1	11	4	2102755	3	x	x	x	15769513	4	x
10108	22	7	3	9	1	51	2	127	1		0	1	10	4	343935	1	x	x	x	1143	2	x
10109	12	1	0	2	1	17	1	70	1		0	1	5	2	10292	1	536086	4	4	0	1	12
10110	22	0	0	2	1	17	1	86	1		0	1	4	1	1925	1	x	x	x	0	1	x
10111	2	112	8	2	1	17	1	524	2		0	1	4	1	881131	2	10303	28866	1	0	1	10
10112	128	35	6	2	1	17	1	188	1		0	1	3	1	630285	2	6219723	15	4	58959	2	13
10114	3	138	52	2	1	17	1	3502	3	1	21421767	4	5	2	5845557	3	24	15413172	1	101795	2	17
10115	30	105	12	2	1	17	1	2274	3		0	1	5	2	1347610	2	810650	343	3	2502527	3	16
10116	16	13	4	2	1	17	1	793	2		0	1	5	2	470947	2	x	x	x	2084023	3	x

HPPD_ID	Cap	r_vol	r_area	RRI_04	RRI_04_Q	RFI_04	RFI_04_Q	Res	Res_Q	PA_No	PA	PA_Q	MF	MF_Q	PE	PE_Q	SE	Lifespan	SE_Q	LU_C	LU_C_Q	SUM_Q
	[MW]	[million m³]	[million m²]	[%]		[%]		count		count	[m²]		count		[m³/year]		[t/year]	[year]		[m²]		
10117	153	3108	232	19	1	75	3	4356	3		0	1	7	3	29202904	4	2811450	2930	2	3964912	3	19
10118	204	400	53	19	1	75	3	3790	3		0	1	11	4	6779901	4	2219537	478	3	0	1	19
10119	120	220	11	19	1	75	3	454	2		0	1	7	3	1302461	2	2125882	274	3	0	1	15
10120	66	1	0	19	1	75	3	21	1	2	277357	3	8	3	14806	1	x	x	x	50575	2	x
10121	8	0	0	19	1	75	3	56	1		0	1	6	2	21002	1	x	x	x	0	1	x
10122	36	109	14	8	1	46	2	759	2		0	1	10	4	1658090	3	89999626	3	4	2309345	3	20
10123	53	28	9	8	1	46	2	425	2		0	1	10	4	1062439	2	2830440	26	4	0	1	17
10124	21	40	11	8	1	46	2	528	2		0	1	10	4	1271314	2	134	785491	1	19666	2	15
10126	13	78	45	8	1	46	2	1264	2		0	1	10	4	5121484	3	321	640204	1	1439141	3	17
10127	6747	704	22	9	1	51	2	2890	3		0	1	11	4	2102755	3	x	x	x	15769513	4	x
10128	14	290	76	8	1	46	2	1341	2		0	1	11	4	8382338	4	1355	567462	1	3645276	3	18
10129	50	2	1	8	1	46	2	30	1		0	1	10	4	92814	1	x	x	x	90819	2	x
10130	38	5	2	8	1	46	2	180	1		0	1	6	2	175322	1	x	x	x	96878	2	x
10131	688	43	58	8	1	46	2	424	2		0	1	12	4	6204484	4	32960266	3	4	2100588	3	21
10132	17	13	8	8	1	46	2	56960	4		0	1	8	3	802942	2	2151459	15	4	6039230	3	21
10133	460	60	17	8	1	46	2	51620	4		0	1	12	4	1838245	3	1531	103727	1	2197300	3	19
10134	50	43	12	8	1	46	2	32309	4		0	1	12	4	1300419	2	3130858	36	4	1843756	3	21

HPPD_ID	Cap	r_vol	r_area	RRI_04	RRI_04_Q	RFI_04	RFI_04_Q	Res	Res_Q	PA_No	PA	PA_Q	MF	MF_Q	PE	PE_Q	SE	Lifespan	SE_Q	LU_C	LU_C_Q	SUM_Q
	[MW]	[million m³]	[million m²]	[%]		[%]		count		count	[m²]		count		[m³/year]		[t/year]	[year]		[m²]		
10135	20	5	3	8	1	46	2	299	2		0	1	8	3	304107	1	8029794	2	4	259235	2	17
10136	50	64	19	8	1	46	2	339	2		0	1	8	3	2069491	3	170	996947	1	0	1	15
10137	3	2	1	8	1	46	2	35	1		0	1	5	2	89107	1	481584	10	4	0	1	13
10138	60	204	31	8	1	46	2	243	1		0	1	6	2	3541083	3	103	5261341	1	0	1	12
10139	42	335	55	8	1	46	2	3916	3		0	1	11	4	5963370	3	1531	579747	1	4807617	3	18
10140	5	516	169	8	1	46	2	5745	4		0	1	9	4	17754936	4	28	49536295	1	449402	2	19
10142	3	40	14	2	1	17	1	244	1		0	1	5	2	1473464	2	x	x	x	448155	2	x
10143	6	1	0	596	4	81	4	4	1	3	451242	3	7	3	14531	1	18571	97	3	0	1	20
10144	6	0	0	596	4	81	4	7	1	3	356789	3	8	3	9687	1	x	x	x	0	1	x
10145	28	8	1	596	4	81	4	20	1	3	4321021	4	8	3	144461	1	27067	815	3	0	1	22
10146	40	5	1	8	1	46	2	1276	2		0	1	5	2	68566	1	1875	7755	2	1047480	3	14
10147	3	2	0	8	1	46	2	128	1		0	1	5	2	35955	1	314310	13	4	0	1	13
10148	5	53	4	8	1	46	2	3551	3		0	1	5	2	426529	2	484848	289	3	4960466	3	17
10149	4	0	0	596	4	81	4	226	1		0	1	7	3	3708	1	704227	2	4	50400	2	20
10150	3	151	2	8	1	46	2	948	2		0	1	5	2	183287	1	31484	12741	2	1980194	3	14
10151	36	35	2	33	2	40	1	26660	4		0	1	6	2	239525	1	4515767	21	4	425724	2	17
10152	240	143	12	33	2	40	1	9568	4		0	1	7	3	1379228	2	1580682	240	3	8137077	4	19
10153	30	350	16	8	1	46	2	9889	4		0	1	5	2	1641090	3	13	71984902	1	8662018	4	18

HPPD_ID	Cap	r_vol	r_area	RRI_04	RRI_04_Q	RFI_04	RFI_04_Q	Res	Res_Q	PA_No	PA	PA_Q	MF	MF_Q	PE	PE_Q	SE	Lifespan	SE_Q	LU_C	LU_C_Q	SUM_Q
	[MW]	[million m³]	[million m²]	[%]		[%]		count		count	[m²]		count		[m³/year]		[t/year]	[year]		[m²]		
10154	10	2	0	8	1	46	2	112	1		0	1	7	3	36164	1	2460773	2	4	226194	2	15
10155	28	22	5	8	1	46	2	264	1		0	1	6	2	611434	2	7493924	8	4	2322675	3	16
10156	15	50	18	33	2	40	1	5911	4	1	2984619	4	7	3	2367283	3	41	3208340	1	554236	2	20
10158	5	8	3	2	1	17	1	49	1		0	1	2	1	366783	2	2067555	10	4	181997	2	13
10167	410	45	10	0	1	47	2	3055	3	1	4934732	4	9	4	960644	2	28	4295234	1	956400	3	20
10174	35	20	2	1	1	45	2	28	1	1	1961687	4	7	3	156324	1	1825	29030	1	0	1	14
10175	73	163	10	1	1	45	2	27	1		0	1	9	4	1019579	2	662424	650	3	0	1	15
10176	180	27	6	0	1	47	2	111	1	1	7248011	4	9	4	586230	2	419	173187	1	181691	2	16
10179	2	95	2	x	x	x	x	446	2		0	1	4	1	147031	1	x	x	x	0	1	x
10180	4	70	2	x	x	x	x	1121	2	1	1735744	4	4	1	134225	1	x	x	x	0	1	x
10181	10	362	9	x	x	x	x	542	2	2	10600677	4	4	1	833926	2	x	x	x	1655710	3	x
10182	2	61	2	x	x	x	x	1343	2		0	1	4	1	192523	1	x	x	x	0	1	x
10183	2	96	3	x	x	x	x	1408	3		0	1	4	1	277818	1	x	x	x	0	1	x
10184	17	5	0	33	2	40	1	1208	2		0	1	11	4	53397	1	9	1341950	1	218822	2	14
10185	8	1041	17	33	2	40	1	24519	4		0	1	6	2	1728134	3	189988	14518	2	17113734	4	19
10201	38	1	0	33	2	40	1	1838	3		0	1	6	2	14817	1	94560	31	4	168867	2	16
10203	68	666	33	596	4	81	4	72091	4		0	1	3	1	3735231	3	66	26659374	1	27946906	4	22

HPPD_ID	Cap	r_vol	r_area	RRI_04	RRI_04_Q	RFI_04	RFI_04_Q	Res	Res_Q	PA_No	PA	PA_Q	MF	MF_Q	PE	PE_Q	SE	Lifespan	SE_Q	LU_C	LU_C_Q	SUM_Q
	[MW]	[million m³]	[million m²]	[%]		[%]		count		count	[m²]		count		[m³/year]		[t/year]	[year]		[m²]		
10204	10	126	6	33	2	40	1	9924	4		0	1	6	2	720399	2	31	10784008	1	5212775	3	16
10206	13	518	8	33	2	40	1	12508	4		0	1	6	2	792751	2	1202	1142650	1	8086749	4	17
10211	1700	571	39	340	4	81	4	1041	2	1	7992235	4	4	1	5289469	3	49915129	30	4	374262	2	24
10212	2000	1536	64	340	4	81	4	10107	4		0	1	3	1	7850060	4	289935	14036	2	15632900	4	24
10213	150	2150	49	292	4	69	3	17217	4		0	1	2	1	6082534	4	42809044	133	3	0	1	21
10214	88	116	18	9	1	6	1	1505	3	1	5124061	4	1	1	2818423	3	74423524	4	4	0	1	18
10215	301	1262	21	340	4	81	4	8693	4		0	1	3	1	2479388	3	1213030	2756	2	14204787	4	23
10232	371	188	4	212	4	75	3	1306	2		0	1	3	1	568337	2	106608	4683	2	2318291	3	18
10233	1600	284	13	340	4	81	4	7762	4		0	1	3	1	1611848	3	240519548	3	4	97033	2	23
10234	278	26	2	340	4	81	4	558	2		0	1	3	1	184313	1	29031	2354	2	0	1	16
10235	265	132	3	340	4	81	4	2063	3		0	1	3	1	291188	1	1895299	184	3	646193	2	19
10236	189	692	7	340	4	81	4	3349	3		0	1	3	1	782156	2	x	x	x	987289	3	x
10238	150	15	2	292	4	69	3	2729	3		0	1	2	1	189238	1	3354485	12	4	478515	2	19
10239	423	16322	466	292	4	69	3	86802	4	1	95257	3	2	1	54336092	4	3539782	12219	2	262473641	4	25
10240	550	483	34	340	4	81	4	9625	4		0	1	3	1	4148831	3	15298335	84	3	1867618	3	23
10241	424	22889	1400	340	4	81	4	139302	4	1	618928156	4	4	1	181219462	4	615	98559989	1	590652171	4	26

HPPD_ID	Cap	r_vol	r_area	RRI_04	RRI_04_Q	RFI_04	RFI_04_Q	Res	Res_Q	PA_No	PA	PA_Q	MF	MF_Q	PE	PE_Q	SE	Lifespan	SE_Q	LU_C	LU_C_Q	SUM_Q
	[MW]	[million m³]	[million m²]	[%]		[%]		count		count	[m²]		count		[m³/year]		[t/year]	[year]		[m²]		
10242	304	76735	2310	340	4	81	4	228107	4	1	844941410	4	4	1	299009007	4	x	x	x	987081649	4	x
10243	467	26	3	8	1	23	1	641	2		0	1	4	1	368146	2	4028602	17	4	0	1	13
10245	507	317	10	8	1	23	1	2881	3		0	1	7	3	1347034	2	12171336	69	4	0	1	15
10246	166	64	1	8	1	23	1	514	2		0	1	6	2	160893	1	2662555	64	4	0	1	13
10247	1700	192	5	8	1	23	1	165	1	2	5531660	4	6	2	585186	2	86554	5865	2	0	1	14
10248	560	353	25	292	4	69	3	1310	2	2	15861142	4	5	2	3658979	3	11603308	81	3	0	1	22
10249	100	173	5	52	3	57	2	286	1		0	1	2	1	807965	2	1698063	270	3	0	1	14
10250	256	878	36	52	3	57	2	1694	3		0	1	2	1	5630728	3	701194	3317	2	403110	2	17
10257	3	8	1	596	4	81	4	1229	2		0	1	5	2	108634	1	478782	46	4	872919	3	21
10259	10	0	0	596	4	81	4	1711	3		0	1	7	3	12075	1	x	x	x	59496	2	x
10280	34	545	30	43	3	63	2	16531	4	1	7325705	4	2	1	4537510	3	216667	6671	2	26007559	4	23
10281	99	300	29	596	4	81	4	97	1	1	30651978	4	5	2	3995771	3	2064545	385	3	19961627	4	25
10282	56	0	0	596	4	81	4	1	1	1	41342	3	5	2	3472	1	118	1710	2	21054	2	19
10283	25	3129	157	596	4	81	4	985	2	2	158439864	4	5	2	21040993	4	1480	5602198	1	148376783	4	25
10290	120	572	19	596	4	81	4	33638	4		0	1	6	2	2547445	3	3144740	482	3	21662990	4	25
10292	2	0	0	596	4	81	4	236	1		0	1	5	2	24309	1	2504060	0	4	193890	2	19
10293	50	291	14	596	4	81	4	2700	3	1	10101411	4	6	2	1961355	3	679036	1137	3	4797928	3	26

HPPD_ID	Cap	r_vol	r_area	RRI_04	RRI_04_Q	RFI_04	RFI_04_Q	Res	Res_Q	PA_No	PA	PA_Q	MF	MF_Q	PE	PE_Q	SE	Lifespan	SE_Q	LU_C	LU_C_Q	SUM_Q
	[MW]	[million m³]	[million m²]	[%]		[%]		count		count	[m²]		count		[m³/year]		[t/year]	[year]		[m²]		
10294	3	0	0	596	4	81	4	986	2	1	137173	3	6	2	15451	1	37715	29	4	137173	2	22
10299	80	450	8	292	4	69	3	5370	3	1	9752066	4	3	1	1045401	2	104972	11352	2	6535877	3	22
10300	45	0	0	292	4	69	3	183	1	1	29928	3	3	1	2086	1	82257	6	4	17096	2	19
10301	20	109	2	292	4	69	3	3587	3		0	1	4	1	304471	1	230746	1251	2	1409279	3	18
10302	40	214	9	43	3	63	2	7607	4		0	1	4	1	1239904	2	399864	1419	2	9602007	4	19
10309	19	1	0	90	3	76	3	1863	3		0	1	4	1	26607	1	116676	24	4	225147	2	18
10311	90	419	28	90	3	76	3	2174	3		0	1	4	1	4365786	3	2618836	424	3	27047178	4	21
10314	693	1169	76	90	3	76	3	2968	3	2	14143859	4	3	1	13157733	4	503707	6153	2	27614105	4	24
10320	6	x	x	0	1	55	2	x	x	x	x	x	x	x	x	x	x	x	x	x	x	x
10322	86	36	3	4	1	51	2	307	2		0	1	1	1	337608	1	2848	33546	1	0	1	10
10323	240	13	1	0	1	23	1	320	2		0	1	1	1	88102	1	6851350	5	4	0	1	12
10340	6	0	0	0	1	55	2	138	1	2	17608	3	1	1	950	1	28841	5	4	0	1	14
10341	19	108	6	0	1	55	2	276	1	1	8505596	4	1	1	702344	2	1107	258408	1	0	1	13
10342	580	5	1	4	1	51	2	111	1	1	865896	3	1	1	113916	1	4966	2701	2	0	1	12
10343	600	505	39	4	1	51	2	2994	3		0	1	1	1	4227982	3	445	3004579	1	2218247	3	15
10344	56	32	4	0	1	55	2	1042	2	1	4746859	4	1	1	376430	2	3805	22058	1	263238	2	15
10348	300	93	1	0	1	55	2	320	2		0	1	1	1	119332	1	2123653	116	3	0	1	12
10350	24	4	0	0	1	23	1	255	1		0	1	1	1	45607	1	187	50420	1	36090	2	9
10354	19	79	3	0	1	55	2	985	2		0	1	1	1	344528	2	84636	2478	2	0	1	12

HPPD_ID	Cap	r_vol	r_area	RRI_04	RRI_04_Q	RFI_04	RFI_04_Q	Res	Res_Q	PA_No	PA	PA_Q	MF	MF_Q	PE	PE_Q	SE	Lifespan	SE_Q	LU_C	LU_C_Q	SUM_Q
	[MW]	[million m³]	[million m²]	[%]		[%]		count		count	[m²]		count		[m³/year]		[t/year]	[year]		[m²]		
10355	20	22	15	0	1	x	x	369	2	1	12119666	4	1	1	1903181	3	2	25721581	1	1643619	3	x
10356	105	102	33	0	1	55	2	4376	3		0	1	1	1	3619914	3	12874384	21	4	22409875	4	19
10357	43	73	24	0	1	55	2	2517	3		0	1	1	1	2630029	3	468	410208	1	14843060	4	16
10358	155	107	34	0	1	55	2	4607	3		0	1	1	1	3703440	3	83	3389135	1	23055227	4	16
10360	41	2033	260	483	4	7	1	81986	4		0	1	3	1	33034776	4	x	x	x	158432029	4	x
10388	3	311	13	596	4	81	4	30260	4		0	1	4	1	1221382	2	437471	1881	2	14811036	4	22
10389	3	301	15	596	4	81	4	36082	4		0	1	4	1	1458361	2	2185	365267	1	16235176	4	21
10390	2	77	2	596	4	81	4	4868	3		0	1	5	2	173678	1	197179	1034	3	1017493	3	21
10392	1	239	5	33	2	40	1	2756	3		0	1	6	2	397760	2	29530	21481	1	5485783	3	15
10393	4	0	0	33	2	40	1	1082	2		0	1	5	2	758	1	24671	5	4	18125	2	15
10395	44	5256	154	596	4	81	4	197701	4		0	1	5	2	15654905	4	930	14978620	1	167698206	4	23
10398	4	6	1	596	4	81	4	2209	3		0	1	4	1	55714	1	51645	285	3	614332	2	19
10402	14	102	45	0	1	21	1	7930	4		0	1	4	1	6460362	4	5	53448255	1	4474941	3	16
10403	120	9	4	596	4	81	4	218	1		0	1	7	3	492205	2	x	x	x	0	1	x
10410	360	89	25	253	4	81	3	1178	2		0	1	6	2	5389381	3	1395372	170	3	4483629	3	21
10411	312	x	x	253	4	81	3	x	x	x	x	x	x	x	x	x	x	x	x	x	x	x
10412	312	47	18	253	4	81	3	2415	3		0	1	7	3	4466772	3	6855	18355	1	1799236	3	21

HPPD_ID	Cap	r_vol	r_area	RRI_04	RRI_04_Q	RFI_04	RFI_04_Q	Res	Res_Q	PA_No	PA	PA_Q	MF	MF_Q	PE	PE_Q	SE	Lifespan	SE_Q	LU_C	LU_C_Q	SUM_Q
	[MW]	[million m³]	[million m²]	[%]		[%]		count		count	[m²]		count		[m³/year]		[t/year]	[year]		[m²]		
10413	420	8	14	253	4	81	3	65	1		0	1	7	3	3170109	3	x	x	x	113239	2	x
10414	205	187	100	254	4	50	2	54823	4		0	1	8	3	18716582	4	163109638	3	4	52326529	4	27
10421	648	243	66	253	4	81	3	3860	3		0	1	6	2	13847909	4	4910	131109	1	9963034	4	22
10429	47	x	x	56	3	47	2	x	x	x	x	x	x	x	x	x	x	x	x	x	x	x
10431	118	3095	52	483	4	7	1	1528	3		0	1	3	1	6828844	4	1165033	7040	2	705704	2	18
10433	4	28	2	33	2	40	1	4650	3		0	1	5	2	271361	1	33130	2241	2	1694898	3	15
10434	8	1	0	33	2	40	1	75	1		0	1	6	2	27418	1	22890534	0	4	124186	2	14
10452	160	363	19	56	3	47	2	453	2	2	29121714	4	4	1	2640784	3	192	5007800	1	58000	2	18
10454	3	0	0	56	3	47	2	12	1		0	1	3	1	2331	1	865367	0	4	0	1	14
10459	60	8	5	56	3	47	2	295	2	2	8679651	4	2	1	729218	2	20	1035049	1	779519	2	17
10462	358	x	x	56	3	47	2	x	x	x	x	x	x	x	x	x	x	x	x	x	x	x
10467	2	112	5	483	4	7	1	455	2		0	1	4	1	547862	2	3256	91086	1	2442993	3	15
10469	300	58	1	483	4	7	1	136	1		0	1	4	1	163458	1	12794	12012	2	0	1	12
10474	520	361	6	483	4	7	1	1031	2		0	1	3	1	782921	2	316379	3022	2	187521	2	15
10475	10	135	6	483	4	7	1	10755	4		0	1	3	1	824157	2	300481	1187	3	6586135	3	19
10478	1	16	10	0	1	x	x	781	2	1	11170634	4	6	2	1314558	2	241	172269	1	10744709	4	x
10479	7	12	1	596	4	81	4	2856	3		0	1	8	3	88405	1	152113	205	3	873491	3	23
10480	350	21	5	596	4	81	4	4999	3	2	434997	3	9	4	622608	2	x	x	x	905966	3	x

HPPD_ID	Cap	r_vol	r_area	RRI_04	RRI_04_Q	RFI_04	RFI_04_Q	Res	Res_Q	PA_No	PA	PA_Q	MF	MF_Q	PE	PE_Q	SE	Lifespan	SE_Q	LU_C	LU_C_Q	SUM_Q
	[MW]	[million m³]	[million m²]	[%]		[%]		count		count	[m²]		count		[m³/year]		[t/year]	[year]		[m²]		
10486	1	982	76	596	4	81	4	56103	4	1	961748	3	7	3	6961673	4	425464	6115	2	15233944	4	28
10515	360	0	0	596	4	81	4	0	1	1	18121	3	9	4	1130	1	x	x	x	0	1	x
10521	11	15	2	596	4	81	4	230	1		0	1	5	2	344244	2	1492384	26	4	0	1	19
10522	5	329	48	596	4	81	4	3987	3		0	1	5	2	6773461	4	1161700	750	3	15826	2	23
10526	45	9	0	596	4	81	4	182	1	1	241058	3	5	2	21905	1	7647	3082	2	0	1	18
10533	8	121	5	596	4	81	4	6640	4		0	1	8	3	485674	2	1061695	302	3	5475686	3	25
10535	38	1	0	596	4	81	4	494	2		0	1	4	1	21926	1	96	14003	2	2377	2	17
10536	109	10	5	596	4	81	4	4334	3		0	1	9	4	643320	2	x	x	x	915839	3	x
10537	2	135	2	596	4	81	4	0	1	2	3969310	4	8	3	222067	1	32047	11147	2	1021895	3	23
10538	7	68	8	596	4	81	4	13456	4		0	1	7	3	1174191	2	518708	347	3	6610101	3	24
10539	7	23	5	596	4	81	4	3890	3		0	1	7	3	780351	2	69	887454	1	3260828	3	21
10542	7	179	4	596	4	81	4	13835	4		0	1	7	3	526368	2	198658	2394	2	3197214	3	23
10543	5	0	0	596	4	81	4	x	x		0	1	6	2	1147	1	108676	1	4	18130	2	x
10544	2	73	1	596	4	81	4	1076	2		0	1	6	2	112370	1	x	x	x	46286	2	x
10545	3	0	0	596	4	81	4	561	2		0	1	6	2	2293	1	1378	195	3	3768	2	19
10547	9	2	0	292	4	69	3	1775	3		0	1	4	1	29406	1	221698	27	4	10792	2	19
10550	26	141	18	2	1	17	1	125	1		0	1	4	1	2166521	3	124	3027324	1	490797	2	11
10567	3	3	1	4	1	54	2	26	1		0	1	6	2	86360	1	1615088	5	4	0	1	13

HPPD_ID	Cap	r_vol	r_area	RRI_04	RRI_04_Q	RFI_04	RFI_04_Q	Res	Res_Q	PA_No	PA	PA_Q	MF	MF_Q	PE	PE_Q	SE	Lifespan	SE_Q	LU_C	LU_C_Q	SUM_Q
	[MW]	[million m³]	[million m²]	[%]		[%]		count		count	[m²]		count		[m³/year]		[t/year]	[year]		[m²]		
10569	381	2274	166	97	3	68	3	5108	3		0	1	3	1	18470886	4	2103602	2865	2	5010206	3	20
10570	260	54	5	97	3	68	3	143	1		0	1	3	1	573004	2	41981	3439	2	341926	2	15
10571	330	3	1	97	3	68	3	55	1		0	1	4	1	109468	1	663993	11	4	0	1	15
10572	453	65	7	97	3	68	3	235	1		0	1	4	1	800569	2	155409	1107	3	0	1	15
10573	460	267	14	97	3	68	3	660	2		0	1	4	1	1607411	3	328424	2157	2	6696	2	17
10574	114	51	3	97	3	68	3	220	1		0	1	4	1	384928	2	24860	5437	2	20005	2	15
10575	120	35557	3202	97	3	68	3	16103	4	2	965594865	4	3	1	366089020	4	222	424049347	1	17345704	4	24
10576	203	47	1	36	2	0	1	116	1		0	1	3	1	145197	1	142937	868	3	0	1	11
10577	121	1151	64	36	2	0	1	2280	3		0	1	4	1	6843337	4	1810115	1685	2	12537037	4	18
10578	217	34	5	49	3	7	1	364	2		0	1	3	1	556315	2	273432	332	3	727849	2	15
10579	283	59	2	49	3	7	1	491	2		0	1	3	1	247541	1	968470	162	3	0	1	13
10581	403	4617	440	49	3	7	1	32827	4		0	1	3	1	46555523	4	9278378	1319	2	127897811	4	20
10583	157	138	10	97	3	68	3	1902	3		0	1	3	1	1111220	2	110153	3331	2	0	1	16
10584	86	41	5	97	3	68	3	231	1		0	1	3	1	576208	2	356	307679	1	0	1	13
10585	57	3	0	97	3	68	3	51	1		0	1	3	1	47089	1	2069821	3	4	0	1	15
10594	41	198	8	2	1	54	2	1932	3		0	1	5	2	880910	2	7360	71323	1	0	1	13
10595	29	5	1	2	1	54	2	752	2		0	1	5	2	102170	1	541839	25	4	0	1	14
10596	190	16	1	2	1	54	2	150	1		0	1	5	2	82785	1	4821819	9	4	8737	2	14

HPPD_ID	Cap	r_vol	r_area	RRI_04	RRI_04_Q	RFI_04	RFI_04_Q	Res	Res_Q	PA_No	PA	PA_Q	MF	MF_Q	PE	PE_Q	SE	Lifespan	SE_Q	LU_C	LU_C_Q	SUM_Q
	[MW]	[million m³]	[million m²]	[%]		[%]		count		count	[m²]		count		[m³/year]		[t/year]	[year]		[m²]		
10597	58	2	1	2	1	54	2	72	1		0	1	5	2	86342	1	249660	24	4	0	1	13
10599	160	17	1	2	1	54	2	235	1		0	1	2	1	140981	1	5803550	8	4	0	1	12
10601	79	2448	195	177	4	51	2	20695	4		0	1	2	1	23016499	4	136	47714073	1	5925109	3	20
10602	230	520	37	177	4	51	2	2604	3		0	1	2	1	4556683	3	14	96293997	1	0	1	16
10603	4	157	18	144	3	33	1	1922	3		0	1	3	1	2154430	3	14	30301703	1	1539872	3	16
10604	74	6785	295	144	3	33	1	5107	3		0	1	3	1	38161111	4	6003014	2995	2	25608759	4	19
10605	8	733	152	16	1	20	1	247	1		0	1	4	1	18475424	4	1284922	1512	2	13594	2	13
10606	48	766	81	97	3	68	3	2285	3		0	1	3	1	9240360	4	327747	6195	2	0	1	18
10608	32	10	1	2	1	17	1	36	1		0	1	3	1	138138	1	524220	50	4	0	1	11
10609	16	11	3	2	1	17	1	583	2		0	1	4	1	394971	2	394538	72	4	0	1	13
10610	33	131	14	2	1	17	1	77	1		0	1	5	2	1645288	3	40	8769163	1	0	1	11
10611	30	11	4	2	1	17	1	1775	3		0	1	3	1	439753	2	4406889	7	4	187600	2	15
10615	120	1653	38	167	4	60	2	4131	3	1	2304675	4	3	1	4115981	3	1061542	4127	2	11937294	4	23
10618	12	6	1	167	4	60	2	469	2		0	1	3	1	70918	1	854111	20	4	192709	2	17
10636	2	0	0	483	4	7	1	249	1		0	1	4	1	18184	1	930266	1	4	182755	2	15
10637	60	7	0	483	4	7	1	39	1	1	135334	3	5	2	51781	1	75968	256	3	335364	2	17
10638	30	29	2	483	4	7	1	312	2		0	1	5	2	244592	1	584863	130	3	768093	2	16

HPPD_ID	Cap	r_vol	r_area	RRI_04	RRI_04_Q	RFI_04	RFI_04_Q	Res	Res_Q	PA_No	PA	PA_Q	MF	MF_Q	PE	PE_Q	SE	Lifespan	SE_Q	LU_C	LU_C_Q	SUM_Q
	[MW]	[million m^3]	[million m^2]	[%]		[%]		count		count	[m^2]		count		[m^3/year]		[t/year]	[year]		[m^2]		
10642	150	789	14	483	4	7	1	2368	3		0	1	4	1	1697339	3	1851278	1129	3	1275210	3	19
10644	28	3242	276	483	4	7	1	38451	4	2	99031829	4	3	1	33314303	4	703864	12207	2	109973078	4	24
10645	3	4445	352	483	4	7	1	44461	4	3	148313437	4	3	1	43552594	4	83	142364523	1	147317369	4	23
10647	50	10	5	483	4	7	1	1317	2		0	1	4	1	710469	2	1145611	23	4	4934560	3	18
10649	50	19	2	483	4	7	1	0	1	1	2046802	4	4	1	224603	1	113	450798	1	0	1	14
10650	55	239	10	483	4	7	1	0	1	1	10534017	4	4	1	1261066	2	307153	2060	2	0	1	16
10651	62	67192	3266	483	4	7	1	676933	4	1	98652337	4	4	1	415055755	4	659724	269899	1	2581392673	4	23
10653	350	1336	64	483	4	7	1	3629	3		0	1	4	1	8832391	4	40467901	87	3	53886044	4	21
10655	37	0	0	483	4	7	1	194	1		0	1	4	1	1956	1	x	x	x	6610	2	x
10657	60	22	8	0	1	27	1	650	2		0	1	3	1	1033304	2	29	2051832	1	0	1	10
10658	55	16	3	0	1	27	1	594	2		0	1	4	1	471392	2	14642447	3	4	453981	2	14
10659	80	22	7	44	3	72	3	1591	3	1	7844308	4	4	1	945389	2	102	566816	1	5106146	3	20
10660	15	23	10	82	3	62	2	182	1		0	1	4	1	1153773	2	x	x	x	5241981	3	x
10666	40	109	14	0	1	30	1	2117	3		0	1	3	1	1867298	3	1490624	195	3	5189	2	15
10668	50	6	2	0	1	30	1	535	2		0	1	3	1	214441	1	4714254	3	4	0	1	12
10669	650	65	25	1070	4	60	2	332	2		0	1	5	2	3616344	3	14	12452332	1	381097	2	17

HPPD_ID	Cap	r_vol	r_area	RRI_04	RRI_04_Q	RFI_04	RFI_04_Q	Res	Res_Q	PA_No	PA	PA_Q	MF	MF_Q	PE	PE_Q	SE	Lifespan	SE_Q	LU_C	LU_C_Q	SUM_Q
	[MW]	[million m³]	[million m²]	[%]		[%]		count		count	[m²]		count		[m³/year]		[t/year]	[year]		[m²]		
10670	200	5	2	1070	4	60	2	72	1		0	1	5	2	336698	1	10560533	1	4	0	1	16
10672	1245	247309	4728	1070	4	60	2	38424	4	2	4608230075	4	5	2	654496164	4	29590259	22148	1	330168092	4	25
10675	37	1	1	44	3	72	3	214	1		0	1	3	1	78162	1	21400	127	3	0	1	14
10676	50	0	0	44	3	72	3	55	1		0	1	3	1	8733	1	x	x	x	0	1	x
10678	20	5	4	141	3	0	1	1119	2	2	1901826	4	6	2	461483	2	5046122	3	4	0	1	19
10680	612	700	17	177	4	51	2	105	1	2	16608402	4	5	2	1871109	3	10	194015391	1	0	1	18
10722	320	83372	2626	509	4	36	1	51394	4	8	4382205770	4	5	2	364860007	4	25195161	8769	2	1211718480	4	25
10741	10	8	2	26	2	52	2	170	1	1	1034517	3	4	1	313033	1	17918026	1	4	541796	2	16
10742	65	326	14	117	3	66	2	450	2		0	1	3	1	1725064	3	559844	1541	2	0	1	15
10743	100	1232	106	117	3	66	2	2085	3	1	851044	3	3	1	13613185	4	521005	6264	2	7055842	4	22
10744	14	9	4	16	1	20	1	126	1		0	1	4	1	487488	2	4784434	5	4	456622	2	13
10745	1	0	0	16	1	20	1	9	1		0	1	3	1	10206	1	155908	4	4	87473	2	12
10748	40	2953	122	16	1	20	1	2264	3	4	67845340	4	4	1	13882976	4	16	494676069	1	0	1	16
10749	4	100	40	117	3	66	2	249	1	2	36935996	4	5	2	5665700	3	13880193	19	4	1595868	3	22
10751	12	95	8	117	3	66	2	149	1		0	1	4	1	955663	2	79	3166745	1	416131	2	13
10754	330	257	14	117	3	66	2	200	1		0	1	3	1	1778971	3	297873	2282	2	0	1	14

HPPD_ID	Cap	r_vol	r_area	RRI_04	RRI_04_Q	RFI_04	RFI_04_Q	Res	Res_Q	PA_No	PA	PA_Q	MF	MF_Q	PE	PE_Q	SE	Lifespan	SE_Q	LU_C	LU_C_Q	SUM_Q
	[MW]	[million m³]	[million m²]	[%]		[%]		count		count	[m²]		count		[m³/year]		[t/year]	[year]		[m²]		
10755	300	15	3	19	1	75	3	102	1		0	1	5	2	344863	2	15557154	3	4	0	1	15
10756	34	323	21	117	3	66	2	499	2	1	40083	3	5	2	2587656	3	19285	44345	1	5864874	3	19
10757	75	1405	68	117	3	66	2	1616	3		0	1	4	1	8552340	4	101	36795691	1	16949	2	17
10758	6	117	10	19	1	75	3	377	2		0	1	4	1	1245484	2	35118	8804	2	385366	2	14
10759	6	10	0	33	2	40	1	14	1		0	1	6	2	33821	1	74	339867	1	0	1	10
10760	132	1417	87	33	2	40	1	939	2	1	89476967	4	6	2	11213106	4	1748562	2147	2	844096	3	20
10761	86	599	21	33	2	40	1	290	1	2	22684516	4	6	2	2712523	3	275298	5767	2	487	2	17
10762	108	1386	46	33	2	40	1	185	1	1	21574061	4	6	2	5916935	3	268765	13665	2	383657	2	17
10765	124	11	3	19	1	75	3	170	1		0	1	6	2	381856	2	12758	2208	2	704597	2	14
10766	68	2701	391	19	1	75	3	10549	4	2	22923904	4	5	2	47435776	4	82	87125305	1	69903429	4	23
10767	202	5	1	19	1	75	3	58	1		0	1	6	2	127412	1	58438	247	3	3212	2	14
10768	372	19	2	19	1	75	3	99	1		0	1	6	2	291952	1	5697450	9	4	0	1	14
10770	200	196	18	44	3	72	3	3457	3		0	1	4	1	2176764	3	2317786	224	3	7947420	4	21
10772	320	83372	2626	509	4	36	1	51394	4	8	4382205770	4	5	2	364860007	4	x	x	x	1211718480	4	x
10783	620	935	70	138	3	50	2	593	2	3	55703698	4	5	2	9813790	4	29923472	83	3	222954	2	22
10785	1	0	0	111	3	27	1	8	1		0	1	3	1	2034	1	57	4380	2	0	1	11
10786	15	7	1	44	3	72	3	338	2		0	1	4	1	130347	1	1121	15458	2	28195	2	15

HPPD_ID	Cap	r_vol	r_area	RRI_04	RRI_04_Q	RFI_04	RFI_04_Q	Res	Res_Q	PA_No	PA	PA_Q	MF	MF_Q	PE	PE_Q	SE	Lifespan	SE_Q	LU_C	LU_C_Q	SUM_Q
	[MW]	[million m³]	[million m²]	[%]		[%]		count		count	[m²]		count		[m³/year]		[t/year]	[year]		[m²]		
10787	5	0	0	44	3	72	3	26	1	1	17042	3	3	1	964	1	313323	1	4	0	1	17
10788	2	1	0	44	3	72	3	147	1		0	1	3	1	39472	1	588863	4	4	169278	2	16
10790	2	1	0	44	3	72	3	342	2		0	1	3	1	29119	1	169442	22	4	0	1	16
10792	13	278	40	28	2	92	4	10943	4		0	1	11	4	5057022	3	481930	1528	2	17343037	4	24
10793	160	1506	109	28	2	92	4	8348	4	2	29048485	4	11	4	13768004	4	185842	21469	1	18536099	4	27
10794	108	177	19	28	2	92	4	2110	3	2	4947310	4	11	4	2396095	3	284800	1643	2	3151148	3	25
10795	29	497	57	28	2	92	4	945	2		0	1	9	4	7239779	4	2196869	599	3	3116142	3	23
10796	15	15	12	28	2	92	4	488	2	1	3648090	4	9	4	1458966	2	91993	443	3	384081	2	23
10797	42	117	25	28	2	92	4	1485	3		0	1	9	4	3211966	3	9834	31564	1	12490658	4	22
10798	26	53	15	28	2	92	4	1021	2		0	1	9	4	1960793	3	213521	663	3	4869373	3	22
10799	24	17	6	28	2	92	4	708	2		0	1	9	4	811236	2	116368	383	3	189188	2	20
10800	79	317	35	28	2	92	4	3229	3		0	1	9	4	4515729	3	482098	1740	2	3382729	3	22
10801	29	34	12	28	2	92	4	459	2	2	6413088	4	9	4	1582931	3	974500	94	3	0	1	23
10803	35	1039	146	28	2	92	4	25083	4	2	83624751	4	10	4	20049459	4	38	71907288	1	7571443	4	27
10804	30	193	49	28	2	92	4	14220	4	1	30486330	4	10	4	6714911	4	678931	752	3	1621087	3	28
10805	20	523	81	28	2	92	4	2722	3	1	67954937	4	10	4	11454389	4	993161	1395	2	28336	2	25
10806	11	72	19	89	3	10	1	572	2		0	1	7	3	3012853	3	861127	221	3	0	1	17

HPPD_ID	Cap	r_vol	r_area	RRI_04	RRI_04_Q	RFI_04	RFI_04_Q	Res	Res_Q	PA_No	PA	PA_Q	MF	MF_Q	PE	PE_Q	SE	Lifespan	SE_Q	LU_C	LU_C_Q	SUM_Q
	[MW]	[million m^3]	[million m^2]	[%]		[%]		count		count	[m^2]		count		[m^3/year]		[t/year]	[year]		[m^2]		
10807	9	40	12	89	3	10	1	411	2	1	4448064	4	6	2	1827766	3	969271	110	3	0	1	20
10808	24	1539	247	89	3	10	1	15432	4	1	78030555	4	6	2	38700561	4	555	7354018	1	173752781	4	23
10809	12	77	21	89	3	10	1	1723	3	1	2910818	4	6	2	3512921	3	345918	590	3	5574455	3	22
10810	8	151	37	46	3	59	2	1025	2	3	73836361	4	7	3	5700694	3	82	4865722	1	1956135	3	21
10811	2	24	6	28	2	92	4	820	2		0	1	9	4	817168	2	290645	218	3	1667668	3	21
10812	4	24	12	103	3	71	3	609	2		0	1	7	3	1870299	3	197544	327	3	3523696	3	21
10813	3	48	15	103	3	71	3	996	2		0	1	6	2	2216609	3	467776	273	3	1100581	3	20
10815	3	15	10	89	3	10	1	181	1	1	698730	3	7	3	1510330	2	1263588	31	4	0	1	18
10817	4	11	3	28	2	92	4	501	2		0	1	10	4	421662	2	534614	53	4	0	1	20
10818	2	5	2	28	2	92	4	303	2		0	1	10	4	308426	1	377481	35	4	0	1	19
10820	11	1959	294	146	3	25	1	7986	4	4	96461901	4	6	2	39935919	4	4265403	1217	3	71417679	4	25
10822	60	759	201	162	4	56	2	12625	4	1	32764363	4	4	1	26967145	4	26971417	75	4	1706711	3	26
10825	5	259	108	20	2	20	1	8109	4	2	38035998	4	4	1	15512754	4	19	36127970	1	4221473	3	20
10827	14	x	x	94	3	70	3	x	x	x	x	x	x	x	x	x	x	x	x	x	x	x
10831	5	10	3	32	2	81	3	14428	4		0	1	7	3	289220	1	1613960	16	4	2244093	3	21
10832	90	68	5	146	3	25	1	609	2	2	2961365	4	7	3	513853	2	42138	4279	2	343970	2	19
10833	90	1486	261	146	3	25	1	48911	4	4	67185888	4	7	3	29323824	4	917614	4291	2	241172188	4	25

HPPD_ID	Cap	r_vol	r_area	RRI_04	RRI_04_Q	RFI_04	RFI_04_Q	Res	Res_Q	PA_No	PA	PA_Q	MF	MF_Q	PE	PE_Q	SE	Lifespan	SE_Q	LU_C	LU_C_Q	SUM_Q
	[MW]	[million m³]	[million m²]	[%]		[%]		count		count	[m²]		count		[m³/year]		[t/year]	[year]		[m²]		
10835	15	110	20	258	4	81	3	8315	4	1	225149	3	10	4	2341349	3	4805028	61	4	17076140	4	29
10836	91	200	49	258	4	81	3	5538	4		0	1	10	4	5573629	3	73644	7187	2	42062687	4	25
10840	28	76	93	258	4	81	3	15466	4	2	18217104	4	9	4	10290973	4	218	917126	1	82288989	4	28
10842	21	34	17	258	4	81	3	1147	2	1	7139248	4	9	4	1953478	3	171	524688	1	2264655	3	24
10845	90	1851	314	146	3	25	1	48855	4	3	54458632	4	8	3	35791474	4	15494944	317	3	304418115	4	26
10846	2	14	5	258	4	81	3	292	1	3	5203355	4	8	3	554745	2	2855419	13	4	169666	2	24
10847	280	2403	208	82	3	76	3	1484	3	1	14701895	4	9	4	22840478	4	541454	11761	2	206011716	4	27
10852	156	108	12	82	3	76	3	7979	4		0	1	9	4	1307808	2	11656430	24	4	7264134	4	25
10853	150	311	61	82	3	76	3	7421	4	3	35147975	4	9	4	6821144	4	530	1555186	1	39132797	4	27
10855	2	12	7	258	4	81	3	804	2		0	1	6	2	783919	2	1102979	28	4	0	1	19
10856	32	89	43	82	3	76	3	584	2	1	14542287	4	7	3	4955468	3	12749626	18	4	13368	2	24
10857	150	2005	204	82	3	76	3	13539	4	2	44594356	4	6	2	23272830	4	4129297	1287	2	23609700	4	26
10858	2	76	2	82	3	76	3	2609	3		0	1	7	3	177203	1	122283	1641	2	1564354	3	19
10859	3	377	13	82	3	76	3	82186	4	1	72456	3	7	3	1427015	2	314776	3170	2	5279203	3	23
10860	225	32	11	32	2	88	4	181	1		0	1	8	3	1143762	2	3569	23893	1	1880257	3	18
10861	20	99	43	32	2	88	4	6971	4	1	94243	3	8	2	4648416	3	1941135	136	3	43212169	4	27

HPPD_ID	Cap	r_vol	r_area	RRI_04	RRI_04_Q	RFI_04	RFI_04_Q	Res	Res_Q	PA_No	PA	PA_Q	MF	MF_Q	PE	PE_Q	SE	Lifespan	SE_Q	LU_C	LU_C_Q	SUM_Q
	[MW]	[million m³]	[million m²]	[%]		[%]		count		count	[m²]		count		[m³/year]		[t/year]	[year]		[m²]		
10863	4	20	5	258	4	81	3	15834	4		0	1	7	3	593013	2	3050	17077	1	4566649	3	21
10865	7	4274	414	258	4	81	3	36639	4	4	40647245	4	7	3	54681106	4	32	354820140	1	105044498	4	27
10866	4	540	79	146	3	25	1	3011	3	3	8012513	4	5	2	10375354	4	531580	2694	2	657411	2	21
10867	56	x	x	32	2	81	3	x	x	x	x	x	x	x	x	x	x	x	x	x	x	x
10868	30	1720	193	32	2	81	3	68347	4	1	2666477	4	7	3	21086523	4	x	x	x	203977227	4	x
10870	50	31	12	32	2	81	3	2889	3		0	1	8	3	1257230	2	306111	269	3	11935091	4	22
10871	50	403	100	32	2	81	3	46629	4		0	1	8	3	10816525	4	5984517	178	3	97999365	4	24
10872	252	13	2	32	2	81	3	942	2		0	1	7	3	184883	1	154074	229	3	1853853	3	18
10876	87	229	95	46	3	59	2	7894	4		0	1	11	4	12525100	4	88	6906617	1	11751312	4	23
10877	43	413	146	94	3	70	3	7710	4		0	1	8	3	19855855	4	9610620	114	3	5785115	3	25
10878	36	1479	188	94	3	70	3	6699	4	1	30149747	4	6	2	25802348	4	23	167759831	1	186745	2	23
10890	45	30	12	32	2	88	4	1322	2		0	1	7	3	1545124	2	885699	88	3	86623	2	19
10891	15	30	12	32	2	88	4	1322	2		0	1	7	3	1545124	3	x	x	x	86623	2	x
10892	13	x	x	32	2	88	4	x	x	x	x	x	x	x	x	x	x	x	x	x	x	x
10893	9	226	51	32	2	88	4	5465	4		0	1	7	3	6578856	4	x	x	x	2768055	3	x
10895	100	189	18	32	2	88	4	2974	3	1	270785	3	6	2	2391767	3	589590	848	3	11238166	4	24

10918	10917	10916	10915	10914	10913	10912	10911	10909	10908	10907	10906	10903	10901	10900	10899	10898		**HPPD_ID**
291	81	84	19	14	24	21	29	40	15	23	27	2	67	80	2	11	**[MW]**	**Cap**
95	95	600	18	231	1462	1069	1818	1031	56	381	751	9	244	268	3	3	**[million m^3]**	**r_vol**
7	7	14	1	16	64	91	128	33	13	28	89	2	16	18	1	1	**[million m^2]**	**r_area**
32	32	32	32	32	32	32	32	32	32	32	32	32	32	32	32	32	**[%]**	**RRI_04**
2	2	2	2	2	2	2	2	2	2	2	2	2	2	2	2	2		**RRI_04_Q**
88	88	88	88	88	88	88	88	88	88	88	88	88	88	88	88	88	**[%]**	**RFI_04**
4	4	4	4	4	4	4	4	4	4	4	4	4	4	4	4	4		**RFI_04_Q**
2838	2838	2452	227	3014	5897	7762	9442	2276	2352	2049	10707	385	2145	2180	289	264	**count**	**Res**
3	3	3	1	3	4	4	4	3	3	3	4	2	3	3	1	1		**Res_Q**
		1										1					**count**	**PA_No**
0	0	1098518	0	0	0	0	0	0	0	0	0	1646476	0	0	0	0	**[m^2]**	**PA**
1	1	3	1	1	1	1	1	1	1	1	1	4	1	1	1	1		**PA_Q**
5	5	5	5	5	5	5	5	5	5	5	5	5	6	6	6	6	**count**	**MF**
2	2	2	2	2	2	2	2	2	2	2	2	2	2	2	2	2		**MF_Q**
897100	897100	1671709	116597	2049947	7995614	11716274	16481535	4104656	1635926	3649057	11126965	202969	2133908	2262646	105507	161903	**[m^3/year]**	**PE**
2	2	3	1	3	4	4	4	3	3	3	4	1	3	3	1	1		**PE_Q**
x	592583	229057	9024	905225	294985	134618	44700	780970	15913	1433315	231	178127	1105722	54	216682	118097	**[t/year]**	**SE**
x	423	6946	5321	675	13130	21040	107781	3497	9358	705	8625250	136	585	13192672	40	61	**[year]**	**Lifespan**
x	3	2	2	3	2	1	1	2	2	3	1	3	3	1	4	4		**SE_Q**
0	0	1303403	448030	5311244	6662101	6125639	8783330	1039164	2949511	1172928	15391821	862771	4583932	3703124	587461	415999	**[m^2]**	**LU_C**
1	1	3	2	3	4	3	4	3	3	3	4	3	3	3	2	2		**LU_C_Q**
x	18	22	15	21	23	21	22	20	20	21	22	21	21	19	17	17		**SUM_Q**

10956	10952	10946	10944	10942	10941	10933	10930	10929	10927	10926	10924	10923	10922	10921	10920	10919		**HPPD_ID**
18	93	1	174	3	3	10	128	5	135	16	82	90	36	90	115	120	**[MW]**	**Cap**
122	297	2	1798	164	1	18	1990	35	2556	25	1273	998	81	87	141	512	**[million m³]**	**r_vol**
1	14	0	57	23	0	5	128	9	152	10	60	35	12	8	11	40	**[million m²]**	**r_area**
32	98	964	32	964	32	32	98	98	32	32	32	32	32	32	32	32	**[%]**	**RRI_04**
2	3	4	2	4	2	2	3	3	2	2	2	2	2	2	2	2		**RRI_04_Q**
88	50	68	88	68	88	88	50	50	88	88	88	88	88	88	88	88	**[%]**	**RFI_04**
4	2	3	4	3	4	4	2	2	4	4	4	4	4	4	4	4		**RFI_04_Q**
155	647	55	9298	3312	148	384	3614	281	5755	1080	2820	4487	2456	1819	2167	5269	**count**	**Res**
1	2	1	4	3	1	2	3	1	4	2	3	3	3	3	3	3		**Res_Q**
						1		1	1		1						**count**	**PA_No**
0	0	0	0	0	0	3412561	0	9731247	16718920	0	9351874	0	0	0	0	0	**[m²]**	**PA**
1	1	1	1	1	1	4	1	4	4	1	4	1	1	1	1	1		**PA_Q**
6	4	4	5	4	5	5	5	5	5	5	5	6	6	6	6	6	**count**	**MF**
2	1	1	2	1	2	2	2	2	2	2	2	2	2	2	2	2		**MF_Q**
160926	1845445	42968	7175296	2969139	22429	653047	17709249	1289970	20274357	1324146	7869937	4547475	1574333	1028968	1395101	5138845	**[m³/year]**	**PE**
1	3	1	4	3	1	2	4	2	4	2	4	3	3	2	2	3		**PE_Q**
25041	2198022	291770	1722702	98854	1670	3363904	6516372	1664554	2042159	2324377	1708387	889907	240131	825529	8175	222146	**[t/year]**	**SE**
12916	358	16	2765	4393	1803	14	809	56	3317	29	1975	2971	895	279	45850	6112	**[year]**	**Lifespan**
2	3	4	2	2	2	4	3	4	2	4	2	2	3	3	1	2		**SE_Q**
0	0	0	4038111	0	0	0	501971	0	387324	0	2458221	22580792	4902767	5171439	6956140	19844808	**[m²]**	**LU_C**
1	1	1	3	1	1	1	2	1	2	1	3	4	3	3	4	4		**LU_C_Q**
14	16	16	22	18	14	21	20	19	24	18	24	21	21	20	19	21		**SUM_Q**

10991	10990	10989	10987	10986	10985	10984	10980	10975	10970	10969	10968	10967	10966	10965	10962		**HPPD_ID**
85	90	72	7	161	149	281	76	12	40	127	98	118	135	58	21	**[MW]**	**Cap**
802	1070	806	35	3670	27	27089	229	473	89	787	349	587	664	503	843	**[million m^3]**	**r_vol**
106	163	153	14	220	1	612	80	45	18	46	24	23	28	15	78	**[million m^2]**	**r_area**
56	56	56	56	964	964	964	964	56	32	32	32	32	32	964	32	**[%]**	**RRI_04**
3	3	3	3	4	4	4	4	3	2	2	2	2	2	4	2		**RRI_04_Q**
30	30	30	30	68	68	68	68	30	88	88	88	88	88	68	88	**[%]**	**RFI_04**
1	1	1	1	3	3	3	3	1	4	4	4	4	4	3	4		**RFI_04_Q**
2001	6861	2261	424	5532	48	31603	9779	8052	2341	2081	5207	786	1121	1586	2182	**count**	**Res**
3	4	3	2	4	1	4	4	4	3	3	3	2	2	3	3		**Res_Q**
2	2	2		2	2	4		2				1	1			**count**	**PA_No**
114380871	164964235	73494252	0	105422144	1896727	366265105	0	47546002	0	0	0	1412598	1216744	0	0	**[m^2]**	**PA**
4	4	4	1	4	4	4	1	4	1	1	1	3	3	1	1		**PA_Q**
8	10	10	8	3	4	4	4	7	5	6	6	9	9	5	5	**count**	**MF**
3	4	4	3	1	1	1	1	3	2	2	2	4	4	2	2		**MF_Q**
14173908	22027089	20985979	1932701	30374598	184088	80572712	10148277	6103194	2395727	6414201	3145750	3249548	3945463	1967740	10071541	**[m^3/year]**	**PE**
4	4	4	3	4	1	4	4	4	3	4	3	3	3	3	4		**PE_Q**
324	x	3810831	555611	2166313	8639878	833	1468963	1418833	475	499058	379591	1941213	98579	94848	13053	**[t/year]**	**SE**
6553085	x	561	167	4490	8	86194849	413	884	498151	4177	2436	801	17857	14051	171132	**[year]**	**Lifespan**
1	x	3	3	2	4	1	3	3	1	2	2	3	1	2	1		**SE_Q**
0	15148843	0	0	8730413	0	2421674	186578	21162041	983612	3219212	1156138	953532	1098980	373198	1677	**[m^2]**	**LU_C**
1	4	1	1	4	1	3	2	4	3	3	3	3	3	2	2		**LU_C_Q**
20	x	23	18	26	19	25	22	26	19	21	20	22	20	20	19		**SUM_Q**

HPPD_ID	Cap	r_vol	r_area	RRI_04	RRI_04_Q	RFI_04	RFI_04_Q	Res	Res_Q	PA_No	PA	PA_Q	MF	MF_Q	PE	PE_Q	SE	Lifespan	SE_Q	LU_C	LU_C_Q	SUM_Q
	[MW]	[million m³]	[million m²]	[%]		[%]		count		count	[m²]		count		[m³/year]		[t/year]	[year]		[m²]		
10992	4	25	4	56	3	30	1	112	1	1	1239979	3	7	3	495623	2	5956	11212	2	0	1	16
10993	5	91	23	56	3	30	1	1851	3	1	21774839	4	7	3	2975186	3	209110	1156	3	625658	2	22
10996	2	55	10	56	3	30	1	284	1	1	10786247	4	8	3	1243792	2	221293	664	3	802137	2	20
10997	27	350	69	56	3	30	1	6161	4	2	2821622	4	7	3	8702373	4	1207307	769	3	633144	2	24
10998	1	24	8	56	3	30	1	483	2		0	1	7	3	1000463	2	74600	850	3	334993	2	17
11000	17	32	11	56	3	30	1	325	2	2	12451667	4	8	3	1423066	2	x	x	x	187218	2	x
11001	36	18	4	56	3	30	1	103	1	1	3484864	4	8	3	466727	2	3609100	13	4	0	1	20
11002	100	1029	225	56	3	30	1	7304	4	2	322811489	4	8	3	29503594	4	7299209	373	3	841813	3	25
11004	6	83	7	56	3	30	1	1343	2		0	1	7	3	871405	2	131899	1666	2	0	1	15
11005	17	1528	146	56	3	30	1	5892	4		0	1	7	3	17296524	4	1389210	2915	2	3153734	3	21
11007	5	66	12	56	3	30	1	995	2		0	1	8	3	1457922	2	646078	272	3	0	1	17
11009	48	107	25	32	2	88	4	3324	3		0	1	6	2	2735909	3	2694221	105	3	11472678	4	22
11010	6	13	14	32	2	88	4	725	2	1	89636	3	5	2	1606522	3	848136	42	4	98162	2	22
11011	3	60	4	32	2	88	4	323	2	1	4511673	4	8	3	461150	2	28902	5515	2	0	1	21
11012	48	36	10	32	2	88	4	3925	3		0	1	6	2	1063459	2	321397	299	3	7477506	4	21
11015	18	247	31	32	2	88	4	8474	4	1	3298966	4	5	2	3513187	3	721401	908	3	28946484	4	26
11016	7	1	0	32	2	88	4	69	1		0	1	5	2	18786	1	319059	7	4	4764	2	17

HPPD_ID	Cap	r_vol	r_area	RRI_04	RRI_04_Q	RFI_04	RFI_04_Q	Res	Res_Q	PA_No	PA	PA_Q	MF	MF_Q	PE	PE_Q	SE	Lifespan	SE_Q	LU_C	LU_C_Q	SUM_Q
	[MW]	[million m³]	[million m²]	[%]		[%]		count		count	[m²]		count		[m³/year]		[t/year]	[year]		[m²]		
11017	8	36	14	32	2	88	4	16247	4		0	1	6	2	1711605	3	1904225	50	4	10051912	4	24
11019	2	103	21	32	2	88	4	4072	3		0	1	8	3	2145897	3	82	3304699	1	19376147	4	22
11021	9	0	0	32	2	88	4	93	1		0	1	8	3	10123	1	661	1580	2	71661	2	17
11023	150	144	40	32	2	88	4	2729	3		0	1	8	3	4421484	3	2829503	135	3	31973698	4	24
11024	518	7652	480	32	2	88	4	32879	4		0	1	8	3	54301816	4	859116	23603	1	223325879	4	24
11029	25	882	528	103	3	71	3	23347	4		0	1	3	1	106927676	4	x	x	x	20145429	4	x
11032	14	0	0	56	3	30	1	317	2		0	1	8	3	9373	1	x	x	x	0	1	x
11036	4	377	115	56	3	30	1	14701	4		0	1	7	3	17551501	4	1470	679541	1	63012907	4	21
11038	24	662	263	11	1	40	1	12161	4	1	133369496	4	5	2	37068545	4	11725626	150	3	88163079	4	23
11039	50	30	11	964	4	68	3	433	2		0	1	3	1	1542745	2	4975103	16	4	0	1	18
11041	30	1095	107	964	4	68	3	1901	3		0	1	2	1	15797011	4	3327066	872	3	12146474	4	23
11042	70	860	113	102	3	20	1	1216	2	1	357805	3	2	1	17672070	4	22984150	99	3	35844817	4	21
11045	18	307	81	1265	4	9	1	21572	4	1	556508	3	4	1	13140229	4	20784016	39	4	48423240	4	25
11047	123	98	52	103	3	71	3	2974	3	5	55096399	4	7	3	9049881	4	x	x	x	22172888	4	x
11048	26	8713	744	103	3	71	3	5492	4	7	2720576957	4	8	3	124541269	4	1241835	18593	1	137668072	4	26

HPPD_ID	Cap	r_vol	r_area	RRI_04	RRI_04_Q	RFI_04	RFI_04_Q	Res	Res_Q	PA_No	PA	PA_Q	MF	MF_Q	PE	PE_Q	SE	Lifespan	SE_Q	LU_C	LU_C_Q	SUM_Q
	[MW]	[million m³]	[million m²]	[%]		[%]		count		count	[m²]		count		[m³/year]		[t/year]	[year]		[m²]		
11051	1870	5005	1120	142	3	1	1	157118	4	4	201191338	4	9	4	150084219	4	94	140485611	1	131328366	4	25
11066	4	616	82	44	3	19	1	32604	4	3	13327781	4	3	1	15283112	4	214771	7600	2	37149586	4	23
11067	25	199	64	212	4	49	2	14392	4		0	1	5	2	11446051	4	x	x	x	3825946	3	x
11068	1	167	15	118	3	64	2	45573	4		0	1	5	2	2603476	3	41953	10575	2	14894933	4	21
11069	360	1035	63	118	3	64	2	10699	4	2	24307154	4	8	3	9620054	4	1476592	1858	2	616519	2	25
11071	40	22	4	49	3	72	3	21958	4		0	1	7	3	551339	2	420601	141	3	875613	3	22
11072	20	33	0	49	3	72	3	91	1	1	257984	3	7	3	33918	1	233399	371	3	0	1	18
11073	1	1	6	49	3	72	3	70	1		0	1	8	3	809292	2	114802	12	4	20966	2	20
11074	1062	2614	499	49	3	72	3	238484	4	2	12962097	4	9	4	69068581	4	x	x	x	91128126	4	x
11075	700	x	x	49	3	72	3	x	x	x	x	x	x	x	x	x	x	x	x	x	x	x
11076	2	0	0	118	3	64	2	12	1		0	1	7	3	1055	1	43	3130	2	0	1	14
11077	1	129	10	28	2	92	4	141567	4		0	1	6	2	1141246	2	20663	16537	1	992706	3	19
11078	6	142	52	28	2	92	4	6253	4	2	25307596	4	7	3	6563427	4	1510260	249	3	1875192	3	27
11079	9	694	76	28	2	92	4	13183	4		0	1	7	3	9145866	4	x	x	x	17121900	4	x
11080	441	x	x	142	3	1	1	x	x	x	x	x	x	x	x	x	x	x	x	x	x	x
11081	2192	4942	439	14	1	49	2	70870	4	3	112057721	4	9	4	47677854	4	912281	14357	2	17430549	4	25
11082	182	68	17	49	3	72	3	3168	3		0	1	7	3	2115989	3	25	7273952	1	75138	2	20

HPPD_ID	Cap	r_vol	r_area	RRI_04	RRI_04_Q	RFI_04	RFI_04_Q	Res	Res_Q	PA_No	PA	PA_Q	MF	MF_Q	PE	PE_Q	SE	Lifespan	SE_Q	LU_C	LU_C_Q	SUM_Q
	[MW]	[million m^3]	[million m^2]	[%]		[%]		count		count	[m^2]		count		[m^3/year]		[t/year]	[year]		[m^2]		
11083	109	135	41	49	3	72	3	26820	4	1	60290	3	7	3	5376437	3	30016950	12	4	14039984	4	27
11084	136	1204	104	49	3	72	3	8859	4	1	67436	3	8	3	13468804	4	17539459	182	3	2039941	3	27
11086	92	566	101	49	3	72	3	9334	4		0	1	9	4	14212349	4	41256634	36	4	28386399	4	27
11088	138	x	x	49	3	72	3	x	x	x	x	x	x	x	x	x	x	x	x	x	x	x
11090	11	103	55	32	2	88	4	16080	4		0	1	7	3	7202220	4	47516	5749	2	51708914	4	24
11093	86	4	4	32	2	88	4	842	2		0	1	8	3	496899	2	1334804	8	4	1088441	3	22
11097	120	39	10	32	2	88	4	6415	4		0	1	8	3	1409637	2	2420	42845	1	7164196	4	22
11098	100	4	1	32	2	88	4	368	2		0	1	8	3	180915	1	428	24544	1	647320	2	17
11100	10	958	58	504	4	5	1	56576	4		0	1	8	3	6717375	4	174537	14545	2	56733622	4	24
11103	24	230	49	28	2	92	4	4859	3	1	13789792	4	8	3	6065310	3	4824097	126	3	38368269	4	27
11104	9	44	14	28	2	92	4	1738	3		0	1	8	3	1767357	3	109469	1053	3	4604150	3	23
11105	7	176	37	28	2	92	4	3894	3		0	1	7	3	4714368	3	625707	747	3	3432073	3	22
11106	8	52	14	28	2	92	4	1550	3		0	1	8	3	1723633	3	192156	718	3	0	1	21
11108	17	1232	125	28	2	92	4	8388	4	3	38880389	4	8	3	16180282	4	805598	4052	2	5906653	3	27
11110	6	19	5	46	3	59	2	436	2	1	273410	3	7	3	605187	2	1283658	39	4	0	1	20
11111	5	33	6	46	3	59	2	1923	3		0	1	7	3	838331	2	180233	485	3	0	1	18
11112	24	128	18	46	3	59	2	5706	4	1	2121116	4	6	2	2427709	3	704230	483	3	2121041	3	24

HPPD_ID	Cap	r_vol	r_area	RRI_04	RRI_04_Q	RFI_04	RFI_04_Q	Res	Res_Q	PA_No	PA	PA_Q	MF	MF_Q	PE	PE_Q	SE	Lifespan	SE_Q	LU_C	LU_C_Q	SUM_Q
	[MW]	[million m³]	[million m²]	[%]		[%]		count		count	[m²]		count		[m³/year]		[t/year]	[year]		[m²]		
11113	13	13	3	46	3	59	2	649	2	1	187340	3	6	2	468375	2	1049533	33	4	2142853	3	21
11114	24	15	11	46	3	59	2	1134	2	2	22489587	4	6	2	1534773	2	996878	39	4	7897897	4	23
11115	15	x	x	46	3	59	2	x	x	x	x	x	x	x	x	x	x	x	x	x	x	x
11118	2100	x	x	x	x	x	x	x	x	x	x	x	x	x	x	x	x	x	x	x	x	x
11148	26	91	3	533	4	56	2	719	2	3	1692724	4	1	1	383897	2	21643	11103	2	550104	2	19
11149	12	11	1	533	4	56	2	406	2	1	320575	3	1	1	83723	1	6177298	5	4	312007	2	19
11152	18	1	0	533	4	56	2	35	1		0	1	1	1	14470	1	283349	12	4	0	1	15
11156	38	136	7	533	4	56	2	2756	3		0	1	1	1	777867	2	1404301	256	3	505590	2	18
11157	30	983	39	533	4	56	2	1539	3	1	3340999	4	1	1	4449079	3	1124501	2316	2	5457411	3	22
11158	63	1003	38	533	4	56	2	120890	4	1	27761625	4	1	1	4290385	3	79	33860652	1	22073197	4	23
11174	30	1467	36	398	4	65	2	21628	4		0	1	1	1	3865234	3	80	48500743	1	26437227	4	20
11175	10	67	12	398	4	65	2	3565	3		0	1	1	1	1259695	2	357669	495	3	3977637	3	19
11178	12	1383	35	109	3	81	4	801	2	2	30640554	4	1	1	4048068	3	1386709	2644	2	7545957	4	23
11185	238	24194	959	117	3	66	2	13120	4	5	629373844	4	5	2	132110856	4	4237145	15131	2	43166557	4	25
11186	160	6782	203	483	4	7	1	45223	4		0	1	5	2	25897728	4	713965	25172	1	108468647	4	20
11187	150	2406	103	483	4	7	1	29626	4		0	1	4	1	13106717	4	1966688	3242	2	37997659	4	21
11191	480	2	0	97	3	68	3	27	1		0	1	2	1	38753	1	x	x	x	0	1	x

HPPD_ID	Cap	r_vol	r_area	RRI_04	RRI_04_Q	RFI_04	RFI_04_Q	Res	Res_Q	PA_No	PA	PA_Q	MF	MF_Q	PE	PE_Q	SE	Lifespan	SE_Q	LU_C	LU_C_Q	SUM_Q
	[MW]	[million m³]	[million m²]	[%]		[%]		count		count	[m²]		count		[m³/year]		[t/year]	[year]		[m²]		
11193	147	205	3	33	2	40	1	4425	3		0	1	6	2	329407	1	352808	1541	2	917657	3	15
11194	287	313	4	33	2	40	1	5367	3		0	1	6	2	443152	2	2673	310179	1	1570194	3	15
11196	17	13	3	32	2	81	3	322	2	1	8307	3	7	3	274459	1	2314401	15	4	2934287	3	21
11197	20	269	51	32	2	81	3	15172	4		0	1	7	3	5551890	3	468713	1518	2	55173850	4	22
11198	51	x	x	32	2	81	3	x	x	x	x	x	x	x	x	x	x	x	x	x	x	x
11202	20	407	31	596	4	81	4	18164	4		0	1	9	4	3919485	3	194326	5551	2	17810819	4	26
11222	1	14	0	x	x	x	x	1451	3		0	1	4	1	39235	1	x	x	x	0	1	x
11223	4	3	1	x	x	x	x	114	1	1	702538	3	4	1	50496	1	x	x	x	0	1	x
11225	3	210	2	x	x	x	x	574	2	1	2693002	4	4	1	194799	1	x	x	x	0	1	x
11226	380	291	19	212	4	75	3	4949	3		0	1	3	1	2890819	3	83622263	9	4	4922066	3	22
11227	140	113	10	90	3	76	3	805	2		0	1	3	1	1672128	3	2113873	141	3	8908075	4	20
11228	80	1	0	260	4	56	2	179	1	1	204498	3	2	1	15081	1	72487	31	4	0	1	17
11229	70	5777	222	2	1	17	1	7968	4		0	1	4	1	25866381	4	1025440	14930	2	146567	2	16
11230	66	63	6	32	2	88	4	581	2		0	1	9	4	788022	2	2167653	77	4	8627	2	21
11231	42	0	0	596	4	81	4	68	1	1	175072	3	9	4	19519	1	114	11355	2	0	1	20
11232	41	3	1	32	2	88	4	45	1		0	1	9	4	81851	1	3105465	2	4	15121	2	19
11233	24	1230	153	102	3	20	1	1423	3	5	132932304	4	4	1	24790782	4	1760109	1852	2	92587908	4	22
11237	12	3	1	2	1	17	1	77	1		0	1	4	1	81086	1	x	x	x	0	1	x

HPPD_ID	Cap	r_vol	r_area	RRI_04	RRI_04_Q	RFI_04	RFI_04_Q	Res	Res_Q	PA_No	PA	PA_Q	MF	MF_Q	PE	PE_Q	SE	Lifespan	SE_Q	LU_C	LU_C_Q	SUM_Q
	[MW]	[million m³]	[million m²]	[%]		[%]		count		count	[m²]		count		[m³/year]		[t/year]	[year]		[m²]		
11238	1	65	22	0	1	52	2	882	2		0	1	6	2	2460543	3	6970856	25	4	661451	2	17
11239	1	139	6	0	1	52	2	304	2		0	1	11	4	659846	2	12466	29489	1	371018	2	15
11241	66	92	12	32	2	88	4	1324	2		0	1	8	3	1736924	3	1342242	181	3	4753216	3	22

Table S5: Proposed hydropower plants of Type *Reservoir* and *unknown*. Each hydropower plant (HPP) can be identified with the respective HPPD_ID from the Renewable Power Plant Database (RePP) Africa (Peters et al., 2023). Rankings including all indicators for the entire continent (Ranking_all continent), and the major basins Congo, Niger, Nile, Volta, Zambezi (Ranking_all basin); rankings leaving out one indicator (ex_...) at a time (River Regulation (RRI), River Fragmentation (RFI), Resettlement (Res), Protected Areas (PA), Megafauna (MF), Potential Evaporation (PE), Sediment Entrapment (SE), and Land Use Change Cropland (LU_C); and rankings for different indicator compositions with A including PA, Res, and PU; B including MF, and SE; and C including PE, RFI, and RRI. Quartile values are indicated with 1 (low impact, ≤1st quartile), 2 (moderate impact, >1st to ≤2nd quartile), 3 (heavy impact, >2nd to ≤3rd quartile), and 4 (severe impact, >3rd quartile).

HPPD_ID	Ranking_all continent	HYBAS_ID	Basin name	Ranking _all basin	Ex_RRI	Ex_RFI	Ex_Res	Ex_PA	Ex_MF	Ex_PE	Ex_SE	Ex_LU_C	Ranking A	Ranking B
10007	2	1030020050			2	2	3	2	1	2	1	2	3	1
10008	2	1030020050			2	2	2	2	1	2	1	2	2	2
10015	4	1030020050			4	4	4	4	4	4	4	4	3	4
10016	3	1030020050			3	2	3	3	2	2	3	2	1	3
10017	2	1030020050			3	2	3	2	2	2	2	2	1	2
10018	4	1030020050			4	4	4	4	3	3	3	4	3	4
10019	2	1030020050			2	2	3	2	1	2	1	2	3	1
10020	2	1030020050			2	2	3	2	2	2	2	2	3	2
10021	2	1030020050			2	2	3	2	2	2	2	2	3	2
10022	1	1030040190			1	1	1	1	1	1	1	1	1	1

HPPD_ID	Ranking_all continent	HYBAS_ID	Basin name	Ranking _all basin	Ex_RRI	Ex_RFI	Ex_Res	Ex_PA	Ex_MF	Ex_PE	Ex_SE	Ex_LU_C	Ranking A	Ranking B
10024	2	10300 22420	Niger	1	1	1	2	2	1	2	1	2	3	1
10025	3	10300 22420	Niger	3	3	3	3	4	3	3	3	4	3	3
10027	4	10300 22420	Niger	3	3	4	3	4	3	4	4	4	3	4
10028	4	10300 22420	Niger	4	4	4	4	4	4	4	4	4	3	4
10033	3	10300 20050			3	3	3	4	3	3	3	4	3	3
10034	3	10300 20050			3	3	3	4	3	3	4	3	1	4
10035	2	10300 20040	Congo	3	2	2	2	1	2	1	2	2	1	3
10036	2	10300 20050			2	2	1	2	2	2	2	1	1	3
10037	1	10300 20040	Congo	2	1	1	2	1	1	1	1	1	2	1
10038	2	10300 20040	Congo	4	3	3	2	2	2	2	2	2	1	3
10043	3	10300 20040	Congo	4	3	3	3	3	2	2	2	3	2	3
10047	4	10300 20050			4	4	4	3	4	4	4	4	1	4
10049	4	10300 20050			4	4	4	4	4	4	4	4	1	4
10051	1	10300 20040	Congo	2	1	1	1	1	1	1	1	1	1	2
10058	3	10300 20040	Congo	4	4	4	4	2	3	3	3	3	1	4
10059	1	10300 20040	Congo	1	1	1	1	1	1	1	1	1	1	1
10060	1	10300 20040	Congo	1	1	1	1	1	1	1	1	1	1	2
10063	1	10300 20040	Congo	2	2	2	2	2	1	1	1	1	1	2

HPPD_ID	Ranking_all continent	HYBAS_ID	Basin name	Ranking _all basin	Ex_RRI	Ex_RFI	Ex_Res	Ex_PA	Ex_MF	Ex_PE	Ex_SE	Ex_LU_C	Ranking A	Ranking B
10064	2	10300 20040	Congo	4	3	3	3	2	2	2	2	2	1	3
10065	1	10300 20040	Congo	2	2	2	2	1	1	1	2	1	1	3
10066	2	10300 20040	Congo	3	2	2	2	1	2	2	2	2	1	4
10069	3	10300 20040	Congo	4	3	4	3	2	3	3	3	2	1	4
10070	1	10300 20040	Congo	1	1	1	1	1	1	1	1	1	1	1
10071	1	10300 20040	Congo	2	2	2	2	1	1	2	1	1	1	2
10074	3	10300 20040	Congo	4	4	4	4	2	3	3	3	3	1	4
10076	1	10300 20040	Congo	1	1	1	1	1	1	1	1	1	1	2
10084	1	10300 20040	Congo	1	1	1	1	1	1	1	1	1	1	2
10090	3	10300 20050			3	3	4	2	3	3	2	3	3	2
10091	1	10300 20040	Congo	2	1	1	1	1	1	1	1	1	1	2
10093	2	10300 20040	Congo	3	2	2	2	2	2	1	2	1	1	4
10103	2	10300 20040	Congo	4	3	2	2	2	2	2	3	2	1	4
10109	1	10300 20040	Congo	1	1	1	1	1	1	1	1	1	1	1
10111	1	10300 20040	Congo	1	1	1	1	1	1	1	1	1	1	1
10112	1	10300 20040	Congo	1	1	1	1	1	1	1	1	1	1	1
10114	2	10300 20040	Congo	3	2	2	2	1	2	1	2	2	1	3
10115	1	10300 20040	Congo	2	2	2	1	2	1	1	1	1	1	2

HPPD_ID	Ranking_all continent	HYBAS_ID	Basin name	Ranking _all basin	Ex_RRI	Ex_RFI	Ex_Res	Ex_PA	Ex_MF	Ex_PE	Ex_SE	Ex_LU_C	Ranking A	Ranking B
10117	3	10300 20040	Congo	4	3	2	2	2	2	2	2	2	1	3
10118	3	10300 20040	Congo	4	3	2	2	2	2	2	2	3	2	3
10119	1	10300 20040	Congo	2	1	1	1	1	1	1	1	1	2	1
10122	3	10300 20040	Congo	4	3	3	3	3	2	2	2	2	2	3
10123	2	10300 20040	Congo	3	2	2	2	2	1	2	1	2	2	2
10124	1	10300 20040	Congo	2	1	1	1	1	1	1	1	1	1	2
10126	2	10300 20040	Congo	3	2	2	2	2	1	1	2	1	1	3
10128	2	10300 20040	Congo	3	2	2	2	2	1	1	2	2	1	3
10131	3	10300 20040	Congo	4	3	3	3	3	2	2	2	3	2	3
10132	3	10300 20040	Congo	4	3	3	3	3	2	3	2	3	2	3
10133	2	10300 20040	Congo	4	3	2	2	2	2	2	3	2	1	4
10134	3	10300 20040	Congo	4	3	3	3	3	2	3	2	3	2	3
10135	1	10300 20040	Congo	2	2	2	2	2	1	2	1	2	2	1
10136	1	10300 20040	Congo	1	1	1	1	1	1	1	1	1	1	2
10137	1	10300 20040	Congo	1	1	1	1	1	1	1	1	1	2	1
10138	1	10300 20040	Congo	1	1	1	1	1	1	1	1	1	1	1
10139	2	10300 20040	Congo	3	2	2	2	2	1	2	2	2	1	3
10140	2	10300 20040	Congo	4	3	2	2	2	2	2	3	2	1	4

HPPD_ID	Ranking_all continent	HYBAS_ID	Basin name	Ranking _all basin	Ex_RRI	Ex_RFI	Ex_Res	Ex_PA	Ex_MF	Ex_PE	Ex_SE	Ex_LU_C	Ranking A	Ranking B
10143	3	10300 34260	Nile	2	2	2	3	2	2	3	2	3	4	1
10145	3	10300 34260	Nile	2	3	3	4	2	3	4	3	4	4	2
10146	1	10300 20040	Congo	1	1	1	1	1	1	1	1	1	1	1
10147	1	10300 20040	Congo	1	1	1	1	1	1	1	1	1	2	1
10148	2	10300 20040	Congo	3	2	2	2	2	2	2	1	1	1	2
10149	3	10300 34260	Nile	2	2	2	3	3	2	3	2	3	4	1
10150	1	10300 20040	Congo	1	1	1	1	1	1	1	1	1	1	1
10151	2	10300 20040	Congo	3	2	2	1	2	2	2	1	2	2	2
10152	3	10300 20040	Congo	4	2	3	2	2	2	2	2	2	1	3
10153	2	10300 20040	Congo	3	2	2	2	2	2	2	2	1	1	3
10154	1	10300 20040	Congo	2	1	1	2	1	1	1	1	1	2	1
10155	1	10300 20040	Congo	2	2	1	2	2	1	1	1	1	2	1
10156	3	10300 20040	Congo	4	3	3	2	2	2	2	3	3	1	4
10158	1	10300 20040	Congo	1	1	1	1	1	1	1	1	1	1	1
10167	3	10300 20050			3	3	3	2	2	3	3	2	1	4
10174	1	10300 20050			1	1	1	1	1	1	1	1	1	2
10175	1	10300 20050			1	1	2	1	1	1	1	1	1	1
10176	2	10300 20050			2	1	2	1	1	1	2	1	1	3

HPPD_ID	Ranking_all continent	HYBAS_ID	Basin name	Ranking _all basin	Ex_RRI	Ex_RFI	Ex_Res	Ex_PA	Ex_MF	Ex_PE	Ex_SE	Ex_LU_C	Ranking A	Ranking B
10184	1	10300 20040	Congo	1	1	1	1	1	1	1	1	1	1	2
10185	2	10300 20040	Congo	4	2	3	2	2	2	2	2	2	1	3
10201	1	10300 20040	Congo	2	1	1	1	2	1	2	1	1	2	1
10203	3	10300 34260	Nile	3	3	3	3	4	4	3	4	3	3	3
10204	1	10300 20040	Congo	2	1	1	1	2	1	1	2	1	1	2
10206	2	10300 20040	Congo	3	2	2	1	2	2	2	2	1	1	3
10211	4	10300 34260	Nile	4	3	4	4	3	4	4	3	4	4	2
10212	4	10300 34260	Nile	4	3	4	4	4	4	4	4	4	4	3
10213	3	10300 40260			2	3	3	3	3	2	3	4	4	2
10214	2	10300 08100			2	2	2	1	2	2	1	2	1	2
10215	4	10300 34260	Nile	3	3	3	3	4	4	4	4	3	4	3
10232	2	10300 34260	Nile	1	1	1	2	2	2	2	2	2	3	1
10233	4	10300 34260	Nile	3	3	3	3	4	4	4	3	4	4	2
10234	1	10300 34260	Nile	1	1	1	2	2	2	2	1	2	4	1
10235	2	10300 34260	Nile	1	2	2	2	2	3	3	2	2	4	1
10238	2	10300 40260			2	2	2	2	3	3	2	2	4	1
10239	4	10300 40260			4	4	4	4	4	4	4	4	3	4
10240	4	10300 34260	Nile	3	3	3	3	4	4	4	3	4	4	2

HPPD_ID	Ranking_all continent	HYBAS_ID	Basin name	Ranking _all basin	Ex_RRI	Ex_RFI	Ex_Res	Ex_PA	Ex_MF	Ex_PE	Ex_SE	Ex_LU_C	Ranking A	Ranking B
10241	4	10300 34260	Nile	4	4	4	4	4	4	4	4	4	3	4
10243	1	10300 34260	Nile	1	1	1	1	1	1	1	1	1	1	1
10245	1	10300 34260	Nile	1	1	1	1	1	1	1	1	1	1	2
10246	1	10300 34260	Nile	1	1	1	1	1	1	1	1	1	1	1
10247	1	10300 34260	Nile	1	1	1	1	1	1	1	1	1	1	2
10248	3	10300 40260			3	3	4	2	3	3	3	4	4	2
10249	1	10300 08100			1	1	1	1	1	1	1	1	3	1
10250	2	10300 08100			1	1	2	2	2	1	2	2	2	2
10257	3	10300 34260	Nile	2	2	2	3	3	3	4	2	3	4	1
10280	4	10300 08110			3	4	3	3	4	4	4	3	2	4
10281	4	10300 34260	Nile	4	4	4	4	4	4	4	4	4	4	3
10282	2	10300 34260	Nile	1	2	2	3	2	2	3	2	2	4	1
10283	4	10300 34260	Nile	4	4	4	4	4	4	4	4	4	3	4
10290	4	10300 34260	Nile	4	4	4	4	4	4	4	4	4	4	3
10292	2	10300 34260	Nile	1	2	2	3	2	2	3	2	2	4	1
10293	4	10300 34260	Nile	4	4	4	4	4	4	4	4	4	4	4
10294	3	10300 34260	Nile	3	3	3	4	3	3	4	3	4	4	2
10299	3	10300 40260			3	3	3	2	4	4	3	3	3	3

HPPD_ID	Ranking_all continent	HYBAS_ID	Basin name	Ranking _all basin	Ex_RRI	Ex_RFI	Ex_Res	Ex_PA	Ex_MF	Ex_PE	Ex_SE	Ex_LU_C	Ranking A	Ranking B
10300	2	10300 40260			2	2	3	2	3	3	2	2	4	1
10301	2	10300 40260			1	1	2	2	2	2	2	2	3	1
10302	2	10300 08110			2	2	2	2	3	2	2	2	2	2
10309	2	10300 08110			2	2	2	2	2	2	1	2	4	1
10311	3	10300 08110			3	3	3	3	3	3	3	2	3	2
10314	4	10300 08110			4	4	4	3	4	4	4	4	3	4
10322	1	10300 35180			1	1	1	1	1	1	1	1	1	1
10323	1	10300 35180			1	1	1	1	1	1	1	1	1	1
10340	1	10300 35180			1	1	1	1	1	1	1	1	2	1
10341	1	10300 35180			1	1	1	1	1	1	1	1	1	1
10342	1	10300 35180			1	1	1	1	1	1	1	1	1	1
10343	1	10300 35180			1	1	1	1	1	1	1	1	1	2
10344	1	10300 35180			1	1	1	1	1	1	1	1	1	2
10348	1	10300 35180			1	1	1	1	1	1	1	1	1	1
10350	1	10300 35180			1	1	1	1	1	1	1	1	1	1
10354	1	10300 35180			1	1	1	1	1	1	1	1	1	1
10356	2	10300 35180			3	2	2	2	3	2	2	2	2	2
10357	1	10300 35180			2	1	1	2	2	1	2	1	1	2

HPPD_ID	Ranking_all continent	HYBAS_ID	Basin name	Ranking _all basin	Ex_RRI	Ex_RFI	Ex_Res	Ex_PA	Ex_MF	Ex_PE	Ex_SE	Ex_LU_C	Ranking A	Ranking B
10358	1	10300 35180			2	1	1	2	2	1	2	1	1	2
10388	3	10300 34260	Nile	3	3	3	3	4	4	4	3	3	4	2
10389	3	10300 34260	Nile	2	2	2	3	3	3	3	3	2	3	2
10390	3	10300 34260	Nile	2	2	2	3	3	3	4	3	3	4	2
10392	1	10300 20040	Congo	2	1	1	1	1	1	1	1	1	1	2
10393	1	10300 20040	Congo	2	1	1	1	1	1	1	1	1	2	1
10395	4	10300 34260	Nile	4	3	3	3	4	4	3	4	3	3	4
10398	2	10300 34260	Nile	1	2	2	2	2	3	3	2	2	4	1
10402	1	10300 34260	Nile	1	2	2	1	2	2	1	2	1	1	3
10410	3	10300 34260	Nile	2	2	3	3	3	3	3	3	3	4	2
10412	3	10300 34260	Nile	2	2	3	3	3	3	3	3	3	3	3
10414	4	10300 34260	Nile	4	4	4	4	4	4	4	4	4	4	4
10421	3	10300 34260	Nile	3	3	3	3	4	3	3	4	3	3	3
10431	2	10300 11660	Zambe zi	3	1	2	2	2	2	1	2	2	2	2
10433	1	10300 20040	Congo	2	1	1	1	1	1	1	1	1	1	2
10434	1	10300 20040	Congo	1	1	1	1	1	1	1	1	1	2	1
10452	2	10300 08110			2	2	2	1	2	2	2	2	1	2
10454	1	10300 08110			1	1	1	1	1	1	1	1	3	1

HPPD_ID	Ranking_all continent	HYBAS_ID	Basin name	Ranking _all basin	Ex_RRI	Ex_RFI	Ex_Res	Ex_PA	Ex_MF	Ex_PE	Ex_SE	Ex_LU_C	Ranking A	Ranking B
10459	2	10300 08110			1	1	2	1	2	2	2	2	1	2
10467	1	10300 11660	Zambe zi	1	1	1	1	1	1	1	1	1	1	1
10469	1	10300 11660	Zambe zi	1	1	1	1	1	1	1	1	1	2	1
10474	1	10300 11660	Zambe zi	1	1	1	1	1	1	1	1	1	2	1
10475	2	10300 11660	Zambe zi	3	2	3	2	2	3	2	2	2	3	2
10479	3	10300 34260	Nile	3	3	3	4	4	3	4	3	4	4	2
10486	4	10300 34260	Nile	4	4	4	4	4	4	4	4	4	4	4
10521	2	10300 34260	Nile	1	2	2	3	2	2	2	2	3	4	1
10522	4	10300 34260	Nile	3	3	3	4	4	4	3	3	4	4	2
10526	2	10300 34260	Nile	1	1	1	3	2	2	2	2	2	4	1
10533	4	10300 34260	Nile	4	4	4	4	4	4	4	4	4	4	3
10535	2	10300 34260	Nile	1	1	1	2	2	2	2	2	2	4	1
10537	3	10300 34260	Nile	3	3	3	4	3	3	4	4	4	4	2
10538	4	10300 34260	Nile	4	3	4	4	4	4	4	4	4	4	3
10539	3	10300 34260	Nile	2	2	2	3	3	3	3	3	3	3	2
10542	4	10300 34260	Nile	3	3	3	3	4	3	4	4	4	4	3
10545	2	10300 34260	Nile	1	2	2	3	2	2	3	2	2	4	1
10547	2	10300 40260			2	2	2	2	3	3	2	2	4	1

HPPD_ID	Ranking_all continent	HYBAS_ID	Basin name	Ranking _all basin	Ex_RRI	Ex_RFI	Ex_Res	Ex_PA	Ex_MF	Ex_PE	Ex_SE	Ex_LU_C	Ranking A	Ranking B
10550	1	10300 20040	Congo	1	1	1	1	1	1	1	1	1	1	1
10567	1	10300 18110			1	1	1	1	1	1	1	1	2	1
10569	3	10300 18110			2	2	3	3	3	2	3	2	3	2
10570	1	10300 18110			1	1	2	1	1	1	1	1	3	1
10571	1	10300 18110			1	1	2	1	1	1	1	1	4	1
10572	1	10300 18110			1	1	2	1	1	1	1	1	3	1
10573	2	10300 18110			1	1	2	2	2	1	2	2	3	1
10574	1	10300 18110			1	1	2	1	1	1	1	1	3	1
10575	4	10300 18110			4	4	4	3	4	4	4	4	2	4
10576	1	10300 18110			1	1	1	1	1	1	1	1	1	1
10577	2	10300 18110			2	2	2	2	2	1	2	1	1	3
10578	1	10300 18110			1	1	1	1	1	1	1	1	2	1
10579	1	10300 18110			1	1	1	1	1	1	1	1	2	1
10581	3	10300 18110			2	3	2	3	3	2	3	2	1	3
10583	1	10300 18110			1	1	1	2	2	1	1	2	3	1
10584	1	10300 18110			1	1	1	1	1	1	1	1	2	1
10585	1	10300 18110			1	1	2	1	1	1	1	1	4	1
10594	1	10300 18110			1	1	1	1	1	1	1	1	1	1

HPPD_ID	Ranking_all continent	HYBAS_ID	Basin name	Ranking _all basin	Ex_RRI	Ex_RFI	Ex_Res	Ex_PA	Ex_MF	Ex_PE	Ex_SE	Ex_LU_C	Ranking A	Ranking B
10595	1	10300 18110			1	1	1	1	1	1	1	1	2	1
10596	1	10300 18110			1	1	1	1	1	1	1	1	2	1
10597	1	10300 18110			1	1	1	1	1	1	1	1	2	1
10599	1	10300 18110			1	1	1	1	1	1	1	1	2	1
10601	3	10300 18110			2	3	2	3	3	2	3	2	2	3
10602	1	10300 18110			1	1	1	2	2	1	2	2	2	1
10603	1	10300 40200			1	1	1	2	2	1	2	1	1	2
10604	2	10300 40200			2	3	2	2	3	2	2	2	1	3
10605	1	10300 11660	Zambe zi	1	1	1	1	1	1	1	1	1	1	1
10606	2	10300 18110			2	2	2	2	2	1	2	2	3	2
10608	1	10300 20040	Congo	1	1	1	1	1	1	1	1	1	1	1
10609	1	10300 20040	Congo	1	1	1	1	1	1	1	1	1	1	1
10610	1	10300 20040	Congo	1	1	1	1	1	1	1	1	1	1	1
10611	1	10300 20040	Congo	2	1	1	1	1	1	1	1	1	1	1
10615	4	10300 12600			3	4	4	3	4	4	4	3	3	4
10618	2	10300 12600			1	1	2	2	2	2	1	2	4	1
10636	1	10300 11660	Zambe zi	1	1	1	2	1	1	1	1	1	3	1
10637	2	10300 11660	Zambe zi	2	1	2	2	1	2	2	1	2	3	1

HPPD_ID	Ranking_all continent	HYBAS_ID	Basin name	Ranking _all basin	Ex_RRI	Ex_RFI	Ex_Res	Ex_PA	Ex_MF	Ex_PE	Ex_SE	Ex_LU_C	Ranking A	Ranking B
10638	1	10300 11660	Zambe zi	2	1	1	2	2	1	2	1	1	3	1
10642	2	10300 11660	Zambe zi	3	2	3	2	2	3	2	2	2	3	2
10644	4	10300 11660	Zambe zi	4	3	4	4	3	4	4	4	4	2	4
10645	4	10300 11660	Zambe zi	4	3	4	3	3	4	3	4	3	1	4
10647	2	10300 11660	Zambe zi	3	1	2	2	2	2	2	1	2	3	1
10649	1	10300 11660	Zambe zi	1	1	1	1	1	1	1	1	1	1	1
10650	1	10300 11660	Zambe zi	2	1	1	2	1	2	1	1	2	2	1
10651	4	10300 11660	Zambe zi	4	3	4	3	3	4	3	4	3	1	4
10653	3	10300 11660	Zambe zi	3	2	4	3	3	3	2	3	2	3	3
10657	1	10300 08110			1	1	1	1	1	1	1	1	1	1
10658	1	10300 08110			1	1	1	1	1	1	1	1	1	1
10659	3	10300 11670			2	2	3	2	3	3	3	2	2	3
10666	1	10300 08110			1	1	1	1	1	1	1	1	1	2
10668	1	10300 08110			1	1	1	1	1	1	1	1	1	1
10669	2	10300 11660	Zambe zi	2	1	1	2	2	2	1	2	2	2	2
10670	1	10300 11660	Zambe zi	2	1	1	2	2	1	2	1	2	4	1
10672	4	10300 11660	Zambe zi	4	4	4	4	4	4	4	4	4	2	4
10675	1	10300 11670			1	1	1	1	1	1	1	1	3	1

HPPD_ID	Ranking_all continent	HYBAS_ID	Basin name	Ranking _all basin	Ex_RRI	Ex_RFI	Ex_Res	Ex_PA	Ex_MF	Ex_PE	Ex_SE	Ex_LU_C	Ranking A	Ranking B
10678	2	10300 40200			2	3	3	2	2	2	2	3	3	2
10680	2	10300 18110			1	2	3	1	2	2	2	2	2	2
10722	4	10300 11660	Zambe zi	4	4	4	4	4	4	4	4	4	2	4
10741	1	10300 11660	Zambe zi	2	1	1	2	1	2	2	1	1	3	1
10742	1	10300 11660	Zambe zi	1	1	1	1	1	1	1	1	1	2	1
10743	3	10300 11660	Zambe zi	4	3	4	3	3	4	3	3	3	2	4
10744	1	10300 11660	Zambe zi	1	1	1	1	1	1	1	1	1	1	1
10745	1	10300 11660	Zambe zi	1	1	1	1	1	1	1	1	1	1	1
10748	1	10300 11660	Zambe zi	2	2	2	1	1	2	1	2	2	1	3
10749	3	10300 11660	Zambe zi	4	3	4	4	2	3	3	3	3	3	3
10751	1	10300 11660	Zambe zi	1	1	1	1	1	1	1	1	1	1	1
10754	1	10300 11660	Zambe zi	1	1	1	1	1	1	1	1	1	2	1
10755	1	10300 20040	Congo	2	1	1	2	1	1	1	1	1	3	1
10756	2	10300 11660	Zambe zi	3	2	2	3	2	2	2	3	2	1	3
10757	2	10300 11660	Zambe zi	2	1	1	2	2	2	1	2	2	1	2
10758	1	10300 20040	Congo	1	1	1	1	1	1	1	1	1	1	1
10759	1	10300 20040	Congo	1	1	1	1	1	1	1	1	1	1	1
10760	3	10300 20040	Congo	4	3	3	3	2	3	2	3	2	1	4

10796	10795	10794	10793	10792	10790	10788	10787	10786	10785	10783	10770	10768	10767	10766	10765	10762	10761	**HPPD_ID**
4	4	4	4	4	1	1	2	1	1	3	3	1	1	4	1	2	2	**Ranking_all continent**
10300 22430	10300 22430	10300 22430	10300 22430	10300 22430	10300 11670	10300 11670	10300 11670	10300 11670	10300 12590	10300 11660	10300 11670	10300 20040	10300 20040	10300 20040	10300 20040	10300 20040	10300 20040	**HYBAS_ID**
										Zambe zi		Congo	Congo	Congo	Congo	Congo	Congo	**Basin name**
										4		1	1	4	1	3	3	**Ranking _all basin**
4	4	4	4	4	1	1	1	1	1	3	3	1	1	4	1	2	2	**Ex_RRI**
3	3	4	4	4	1	1	1	1	1	4	3	1	1	4	1	2	2	**Ex_RFI**
4	4	4	4	4	2	2	2	1	1	4	3	1	1	3	1	2	2	**Ex_Res**
3	4	4	4	4	2	2	1	1	1	2	3	1	1	3	1	1	1	**Ex_PA**
3	3	4	4	3	2	2	2	1	1	3	3	1	1	4	1	2	2	**Ex_MF**
4	3	4	4	4	2	2	2	1	1	3	3	1	1	3	1	1	1	**Ex_PE**
3	3	4	4	4	1	1	1	1	1	3	3	1	1	4	1	2	2	**Ex_SE**
4	4	4	4	4	2	1	2	1	1	4	2	1	1	3	1	2	2	**Ex_LU_C**
3	3	3	2	3	4	4	4	3	1	3	3	3	2	1	1	1	1	**Ranking A**
3	3	4	4	4	1	1	1	1	1	3	2	1	1	4	1	2	2	**Ranking B**

HPPD_ID	Ranking_all continent	HYBAS_ID	Basin name	Ranking _all basin	Ex_RRI	Ex_RFI	Ex_Res	Ex_PA	Ex_MF	Ex_PE	Ex_SE	Ex_LU_C	Ranking A	Ranking B
10797	3	10300 22430			3	3	3	4	3	3	4	3	2	4
10798	3	10300 22430			3	3	4	4	3	3	3	3	3	3
10799	3	10300 22430			3	2	3	3	2	3	2	3	3	2
10800	3	10300 22430			3	3	3	4	3	3	3	3	3	3
10801	4	10300 22430			4	3	4	3	3	4	3	4	3	3
10803	4	10300 22430			4	4	4	4	4	4	4	4	2	4
10804	4	10300 22430			4	4	4	4	4	4	4	4	3	4
10805	4	10300 22430			4	4	4	4	4	4	4	4	3	4
10806	2	10300 22420	Niger	1	1	2	2	2	1	1	1	2	2	2
10807	2	10300 22420	Niger	2	2	3	3	2	2	2	2	3	2	2
10808	4	10300 22420	Niger	3	3	4	3	3	4	3	4	3	1	4
10809	3	10300 22420	Niger	3	3	4	3	2	3	3	3	3	2	4
10810	3	10300 23300	Volta	2	3	3	3	2	3	3	3	3	1	4
10811	3	10300 22430			3	2	3	3	2	3	3	3	3	2
10812	3	10300 22420	Niger	2	3	3	3	3	3	3	3	3	3	2
10813	3	10300 22420	Niger	2	2	2	3	3	3	2	2	2	3	2
10815	2	10300 22420	Niger	1	2	2	3	2	2	2	1	2	3	2
10817	3	10300 22430			3	2	3	3	2	3	2	3	4	2

HPPD_ID	Ranking_all continent	HYBAS_ID	Basin name	Ranking _all basin	Ex_RRI	Ex_RFI	Ex_Res	Ex_PA	Ex_MF	Ex_PE	Ex_SE	Ex_LU_C	Ranking A	Ranking B
10818	2	10300 22430			2	2	3	2	2	3	2	3	4	1
10820	4	10300 23310			4	4	4	4	4	4	4	4	2	4
10822	4	10300 23300	Volta	4	4	4	4	4	4	4	4	4	4	4
10825	3	10300 23300	Volta	1	3	3	2	2	3	2	3	2	1	4
10831	3	10300 23310			3	3	3	3	3	4	2	3	3	2
10832	2	10300 23310			2	3	3	2	2	2	2	2	1	3
10833	4	10300 23310			4	4	4	4	4	4	4	4	1	4
10835	4	10300 23310			4	4	4	4	4	4	4	4	4	4
10836	4	10300 23310			4	4	4	4	4	4	4	4	3	4
10840	4	10300 23310			4	4	4	4	4	4	4	4	3	4
10842	4	10300 23310			3	4	4	3	3	4	4	4	3	4
10845	4	10300 23310			4	4	4	4	4	4	4	4	2	4
10846	4	10300 23310			3	4	4	3	3	4	3	4	4	2
10847	4	10300 23310			4	4	4	4	4	4	4	4	3	4
10852	4	10300 23310			4	4	4	4	4	4	4	4	4	4
10853	4	10300 23310			4	4	4	4	4	4	4	4	2	4
10855	2	10300 23310			2	2	3	2	2	2	2	3	4	1
10856	4	10300 23310			4	4	4	3	4	4	3	4	4	3

HPPD_ID	Ranking_all continent	HYBAS_ID	Basin name	Ranking _all basin	Ex_RRI	Ex_RFI	Ex_Res	Ex_PA	Ex_MF	Ex_PE	Ex_SE	Ex_LU_C	Ranking A	Ranking B
10857	4	10300 23310			4	4	4	4	4	4	4	4	3	4
10858	2	10300 23310			2	2	2	2	2	3	2	2	3	2
10859	4	10300 23310			3	4	3	3	3	4	4	4	3	4
10860	2	10300 23310			2	1	3	2	1	2	2	2	2	2
10861	4	10300 23310			4	4	4	4	4	4	4	4	3	4
10863	3	10300 23310			2	3	3	3	3	3	3	3	3	3
10865	4	10300 23310			4	4	4	4	4	4	4	4	3	4
10866	3	10300 23310			3	4	3	2	3	2	3	3	1	4
10870	3	10300 23310			3	3	3	4	3	4	3	3	3	3
10871	4	10300 23310			4	4	4	4	4	4	4	4	3	4
10872	2	10300 23310			2	2	2	2	2	2	2	2	3	2
10876	4	10300 23300	Volta	2	3	4	3	4	3	3	4	3	1	4
10877	4	10300 23300	Volta	4	4	4	4	4	4	4	4	4	3	4
10878	4	10300 23300	Volta	2	3	4	3	3	4	3	4	4	2	4
10890	2	10300 23310			2	2	3	2	2	2	2	2	3	2
10895	4	10300 23310			4	4	4	4	4	4	4	4	3	4
10898	2	10300 23310			2	1	2	2	2	2	1	2	4	1
10899	2	10300 23310			2	1	2	2	2	2	1	2	4	1

HPPD_ID	Ranking_all continent	HYBAS_ID	Basin name	Ranking _all basin	Ex_RRI	Ex_RFI	Ex_Res	Ex_PA	Ex_MF	Ex_PE	Ex_SE	Ex_LU_C	Ranking A	Ranking B
10900	2	10300 23310			2	2	2	2	2	2	3	2	2	2
10901	3	10300 23310			3	2	3	3	3	3	3	3	3	2
10903	3	10300 23310			3	2	3	2	3	4	3	3	3	2
10906	3	10300 23310			3	3	3	4	3	3	4	3	2	4
10907	3	10300 23310			3	2	3	3	3	3	3	3	3	2
10908	3	10300 23310			3	2	3	3	3	2	3	2	3	2
10909	3	10300 23310			3	2	3	3	3	2	3	2	3	2
10911	3	10300 23310			3	3	3	4	3	3	4	3	2	4
10912	3	10300 23310			3	2	3	3	3	2	3	3	2	3
10913	4	10300 23310			4	3	3	4	4	3	4	3	3	4
10914	3	10300 23310			3	2	3	3	3	3	3	3	3	2
10915	1	10300 23310			1	1	2	1	1	1	1	1	3	1
10916	3	10300 23310			3	3	3	3	3	3	3	3	3	3
10917	2	10300 23310			2	1	2	2	2	2	2	2	3	1
10919	3	10300 23310			3	2	3	3	3	3	3	2	3	3
10920	2	10300 23310			2	2	2	2	2	2	3	2	2	2
10921	3	10300 23310			3	2	3	3	3	3	2	2	3	2
10922	3	10300 23310			3	2	3	3	3	3	3	3	3	2

HPPD_ID	Ranking_all continent	HYBAS_ID	Basin name	Ranking _all basin	Ex_RRI	Ex_RFI	Ex_Res	Ex_PA	Ex_MF	Ex_PE	Ex_SE	Ex_LU_C	Ranking A	Ranking B
10923	3	10300 23310			3	2	3	3	3	3	3	2	3	3
10924	4	10300 23310			4	4	4	3	4	4	4	4	3	4
10926	2	10300 23310			2	1	2	2	2	2	1	2	4	1
10927	4	10300 23310			4	4	4	3	4	4	4	4	3	4
10929	2	10300 23310			2	2	3	2	2	2	2	3	3	2
10930	3	10300 23310			2	3	3	3	3	2	2	3	3	2
10933	3	10300 23310			3	2	3	2	3	3	2	4	4	2
10941	1	10300 23310			1	1	1	1	1	1	1	1	3	1
10942	2	10300 27410			1	1	2	2	2	2	2	2	3	1
10944	3	10300 23310			3	3	3	4	3	3	3	3	3	3
10946	1	10300 27410			1	1	2	2	2	2	1	2	4	1
10952	1	10300 23310			1	1	2	2	2	1	1	2	3	1
10956	1	10300 23310			1	1	1	1	1	1	1	1	3	1
10962	2	10300 23310			2	2	2	2	2	2	3	2	2	2
10965	3	10300 27410			2	2	3	3	3	2	3	3	3	2
10966	3	10300 23310			3	2	3	2	3	2	3	2	2	4
10967	4	10300 23310			3	3	4	3	3	3	3	3	3	4
10968	3	10300 23310			3	2	3	3	3	2	3	2	3	2

HPPD_ID	Ranking_all continent	HYBAS_ID	Basin name	Ranking _all basin	Ex_RRI	Ex_RFI	Ex_Res	Ex_PA	Ex_MF	Ex_PE	Ex_SE	Ex_LU_C	Ranking A	Ranking B
10969	3	10300 23310			3	2	3	3	3	2	3	3	3	3
10970	2	10300 23310			2	2	2	2	2	2	3	2	2	2
10975	4	10300 22420	Niger	4	4	4	4	4	4	4	4	4	2	4
10980	3	10300 27410			3	3	3	4	4	3	3	4	4	2
10984	4	10300 27410			4	4	4	4	4	4	4	4	3	4
10985	2	10300 27410			2	2	3	2	3	3	2	3	4	1
10986	4	10300 27410			4	4	4	4	4	4	4	4	3	4
10987	2	10300 22420	Niger	1	2	2	2	2	1	2	2	2	2	2
10989	4	10300 22420	Niger	3	3	4	4	3	3	3	3	4	2	4
10991	3	10300 22420	Niger	2	2	3	3	2	2	2	3	3	1	4
10992	1	10300 22420	Niger	1	1	1	2	1	1	1	1	2	1	2
10993	3	10300 22420	Niger	3	3	4	3	2	3	3	3	4	2	4
10996	2	10300 22420	Niger	2	2	3	3	2	2	3	2	3	2	2
10997	4	10300 22420	Niger	4	4	4	4	3	4	4	4	4	2	4
10998	2	10300 22420	Niger	1	1	2	2	2	1	2	1	2	2	2
11001	2	10300 22420	Niger	2	2	3	3	2	2	3	2	3	3	2
11002	4	10300 22420	Niger	4	4	4	4	4	4	4	4	4	2	4
11004	1	10300 22420	Niger	1	1	1	1	1	1	1	1	1	1	1

HPPD_ID	Ranking_all continent	HYBAS_ID	Basin name	Ranking _all basin	Ex_RRI	Ex_RFI	Ex_Res	Ex_PA	Ex_MF	Ex_PE	Ex_SE	Ex_LU_C	Ranking A	Ranking B
11005	3	1030022420	Niger	2	3	4	3	3	3	2	3	3	1	4
11007	1	1030022420	Niger	1	1	2	2	2	1	2	1	2	2	1
11009	3	1030023310			3	3	3	4	3	3	3	3	3	3
11010	3	1030023310			3	3	4	3	3	3	3	4	4	2
11011	3	1030023310			3	2	3	2	2	3	3	4	3	2
11012	3	1030023310			3	2	3	3	3	3	3	2	3	2
11015	4	1030023310			4	4	4	4	4	4	4	4	3	4
11016	2	1030023310			2	1	2	2	2	2	1	2	4	1
11017	4	1030023310			4	4	4	4	4	4	3	4	4	3
11019	3	1030023310			3	3	3	4	3	3	4	3	2	3
11021	1	1030023310			2	1	2	2	1	2	2	2	3	1
11023	4	1030023310			4	4	4	4	3	4	4	4	3	3
11024	4	1030023310			4	4	4	4	3	4	4	4	2	4
11036	3	1030022420	Niger	2	3	4	3	3	3	2	3	2	1	4
11038	4	1030022420	Niger	3	4	4	3	3	4	3	3	3	1	4
11039	2	1030027410			1	1	2	2	2	2	1	2	4	1
11041	4	1030027410			3	4	4	4	4	3	3	3	4	3
11042	3	1030027410			3	4	3	2	3	2	3	2	2	3

HPPD_ID	Ranking_all continent	HYBAS_ID	Basin name	Ranking _all basin	Ex_RRI	Ex_RFI	Ex_Res	Ex_PA	Ex_MF	Ex_PE	Ex_SE	Ex_LU_C	Ranking A	Ranking B
11045	4	10300 27410			4	4	4	4	4	4	4	4	3	4
11048	4	10300 22420	Niger	4	4	4	4	4	4	4	4	4	2	4
11051	4	10300 22420	Niger	4	4	4	4	4	4	4	4	4	1	4
11066	4	10300 22420	Niger	3	3	4	3	3	4	3	4	3	1	4
11068	3	10300 22420	Niger	2	3	3	3	3	3	3	3	2	2	3
11069	4	10300 22420	Niger	4	4	4	4	4	4	4	4	4	2	4
11071	3	10300 22420	Niger	3	3	3	3	4	3	4	3	3	3	3
11072	2	10300 22420	Niger	1	2	2	3	2	2	2	2	2	3	1
11073	2	10300 22420	Niger	2	2	2	3	3	2	3	2	3	4	1
11076	1	10300 22420	Niger	1	1	1	1	1	1	1	1	1	2	1
11077	2	10300 22430			2	2	2	2	2	2	3	2	2	2
11078	4	10300 22430			4	4	4	4	4	4	4	4	3	4
11081	4	10300 20050			4	4	4	4	4	4	4	4	1	4
11082	2	10300 22420	Niger	2	2	2	3	3	2	2	3	3	2	2
11083	4	10300 22420	Niger	4	4	4	4	4	4	4	4	4	4	4
11084	4	10300 22420	Niger	4	4	4	4	4	4	4	4	4	3	4
11086	4	10300 22420	Niger	4	4	4	4	4	4	4	4	4	4	4
11090	4	10300 23310			4	4	4	4	4	4	4	4	3	4

HPPD_ID	Ranking_all continent	HYBAS_ID	Basin name	Ranking _all basin	Ex_RRI	Ex_RFI	Ex_Res	Ex_PA	Ex_MF	Ex_PE	Ex_SE	Ex_LU_C	Ranking A	Ranking B
11093	3	10300<br>23310			3	3	4	4	3	4	3	3	4	2
11097	3	10300<br>23310			3	3	3	4	3	4	4	3	2	3
11098	1	10300<br>23310			2	1	2	2	1	2	2	2	2	1
11100	4	10300<br>23300	Volta	2	3	4	4	4	3	4	4	4	2	4
11103	4	10300<br>22430			4	4	4	4	4	4	4	4	3	4
11104	3	10300<br>22430			4	3	4	4	3	4	3	4	3	3
11105	3	10300<br>22430			3	3	3	4	3	3	3	3	3	3
11106	3	10300<br>22430			3	2	3	3	2	3	3	4	3	2
11108	4	10300<br>22430			4	4	4	4	4	4	4	4	3	4
11110	3	10300<br>23300	Volta	1	2	3	3	2	2	3	2	3	3	2
11111	2	10300<br>23300	Volta	1	2	2	2	2	2	2	2	2	3	2
11112	4	10300<br>23300	Volta	4	4	4	4	3	4	4	4	4	3	4
11113	3	10300<br>23300	Volta	2	3	3	3	2	3	3	2	3	3	2
11114	4	10300<br>23300	Volta	2	3	4	4	3	4	4	3	3	3	3
11148	2	10300<br>27430			2	2	3	2	3	2	2	2	3	2
11149	2	10300<br>27430			2	2	3	2	3	3	2	2	4	1
11152	1	10300<br>27430			1	1	2	1	1	1	1	1	4	1
11156	2	10300<br>27430			1	2	2	2	2	2	2	2	3	1

HPPD_ID	Ranking_all continent	HYBAS_ID	Basin name	Ranking _all basin	Ex_RRI	Ex_RFI	Ex_Res	Ex_PA	Ex_MF	Ex_PE	Ex_SE	Ex_LU_C	Ranking A	Ranking B
11157	3	10300 27430			3	4	3	2	4	3	3	3	3	3
11158	4	10300 27430			3	4	3	3	4	4	4	3	2	4
11174	3	10300 27430			2	3	2	3	3	2	3	2	2	3
11175	2	10300 27430			2	2	2	2	3	2	2	2	3	2
11178	4	10300 29810			3	3	4	3	4	4	4	3	3	3
11185	4	10300 11660	Zambe zi	4	4	4	4	4	4	4	4	4	2	4
11186	3	10300 11660	Zambe zi	3	2	3	2	3	3	2	3	2	1	4
11187	3	10300 11660	Zambe zi	3	2	4	3	3	3	2	3	2	2	3
11193	1	10300 20040	Congo	2	1	1	1	1	1	1	1	1	1	2
11194	1	10300 20040	Congo	2	1	1	1	1	1	1	1	1	1	2
11196	3	10300 23310			3	3	3	2	3	4	2	3	3	2
11197	3	10300 23310			3	3	3	4	3	3	3	3	2	4
11202	4	10300 34260	Nile	4	4	4	4	4	4	4	4	4	4	4
11226	3	10300 34260	Nile	3	3	3	3	4	4	3	3	3	4	2
11227	3	10300 08110			2	2	3	3	3	2	2	2	3	2
11228	2	10300 15850			1	1	2	1	2	2	1	2	4	1
11229	1	10300 20040	Congo	2	2	2	1	2	2	1	1	1	1	2
11230	3	10300 23310			3	2	3	3	2	3	2	3	4	2

HPPD_ID	Ranking_all continent	HYBAS_ID	Basin name	Ranking _all basin	Ex_RRI	Ex_RFI	Ex_Res	Ex_PA	Ex_MF	Ex_PE	Ex_SE	Ex_LU_C	Ranking A	Ranking B
11231	3	10300 34260	Nile	2	2	2	3	2	2	3	3	3	4	2
11232	2	10300 23310			2	2	3	2	2	3	2	2	4	1
11233	3	10300 27410			3	4	3	2	4	3	3	3	1	4
11238	2	10300 20040	Congo	3	2	2	2	2	2	1	1	2	2	2
11239	1	10300 20040	Congo	2	1	1	1	1	1	1	1	1	1	2
11241	3	10300 23310			3	3	4	4	3	3	3	3	3	2

Table S6: Spearman's correlation coefficients for correlations between the individual indicators including reservoir size. Dataset 1 includes hydropower plants of type reservoir and unknown type. Dataset 2 only includes hydropower plants of type reservoir.

	Reservoir Size		River Regulation		River Fragmentation		Resettlement		Protected Area		Megafauna		Potential Evaporation		Sediment Entrapment (Lifespan)		Land Use Change (Cropland)	
**Dataset**	**1**	**2**	**1**	**2**	**1**	**2**	**1**	**2**	**1**	**2**	**1**	**2**	**1**	**2**	**1**	**2**	**1**	**2**
**Reservoir size**	1	1	0.177	0.211	-0.071	0.003	0.500	0.286	0.756	0.841	-0.033	0.004	0.996	0.995	0.283	0.293	0.647	0.602
**River Regulation**	0.177	0.211	1	1	0.044	0.122	0.148	0.198	0.173	0.219	-0.245	-0.237	0.189	0.228	0.002	-0.029	0.132	0.154
**River Fragmentation**	-0.071	0.003	0.044	0.123	1	1	-0.076	0.015	-0.021	-0.003	0.193	0.143	-0.069	0.004	-0.041	-0.019	-0.109	-0.041
**Resettlement**	0.500	0.286	0.148	0.198	-0.076	0.015	1	1	0.093	0.194	-0.018	-0.025	0.488	0.281	0.078	0.077	0.842	-0.487
**Protected Area**	0.756	0.841	0.173	0.219	-0.021	-0.003	0.093	0.194	1	1	-0.008	0.027	0.795	0.881	0.079	0.071	0.365	0.709
**Megafauna**	-0.033	0.004	-0.245	-0.237	0.193	0.143	-0.018	-0.025	-0.008	0.027	1	1	-0.034	0.004	-0.041	0.024	-0.034	0.024
**Potential Evaporation**	0.996	0.995	0.189	0.228	-0.096	0.004	0.488	0.281	0.795	0.881	-0.034	0.004	1	1	0.254	0.253	0.645	0.619
**Sediment Entrapment (Lifetime)**	0.283	0.293	0.002	-0.029	-0.041	-0.019	0.078	0.077	0.079	0.071	-0.041	0.024	0.255	0.253	1	1	0.034	0.019
**Land Use Change (Cropland)**	0.647	0.602	0.132	0.154	-0.109	-0.041	0.842	0.487	0.365	0.709	-0.034	0.024	0.645	0.619	0.034	0.019	1	1

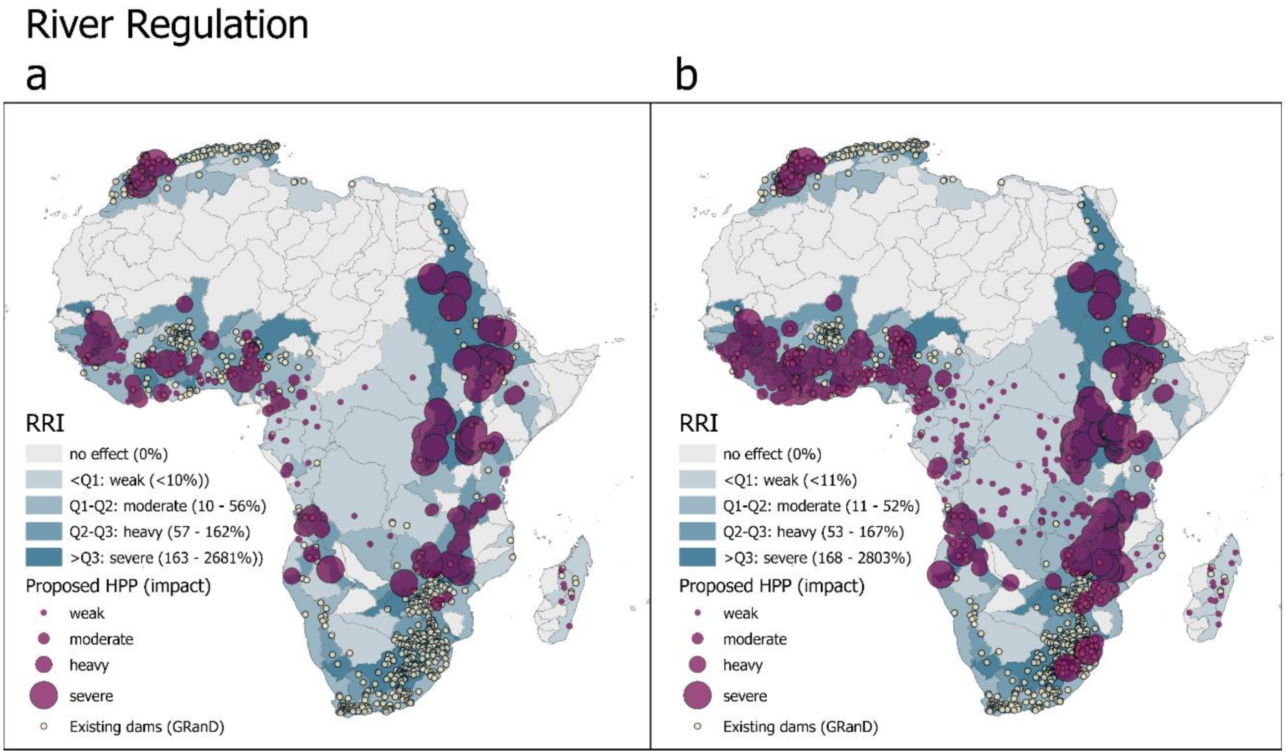

Figure S1: Results of impact assessment for the indicator River Regulation (RRI: River Regulation Index) for hydropower plants (HPPs) of type reservoir (a) and of type reservoir and unknown (b). Purple circles indicate proposed HPPs. Small yellow circles indicate existing dam infrastructure (GRanD). Impact of proposed HPPs increases with circle size. The base map shows RRI values divided according to the quartile values (Q) for sub-basins (HydroBASIN Level 4).

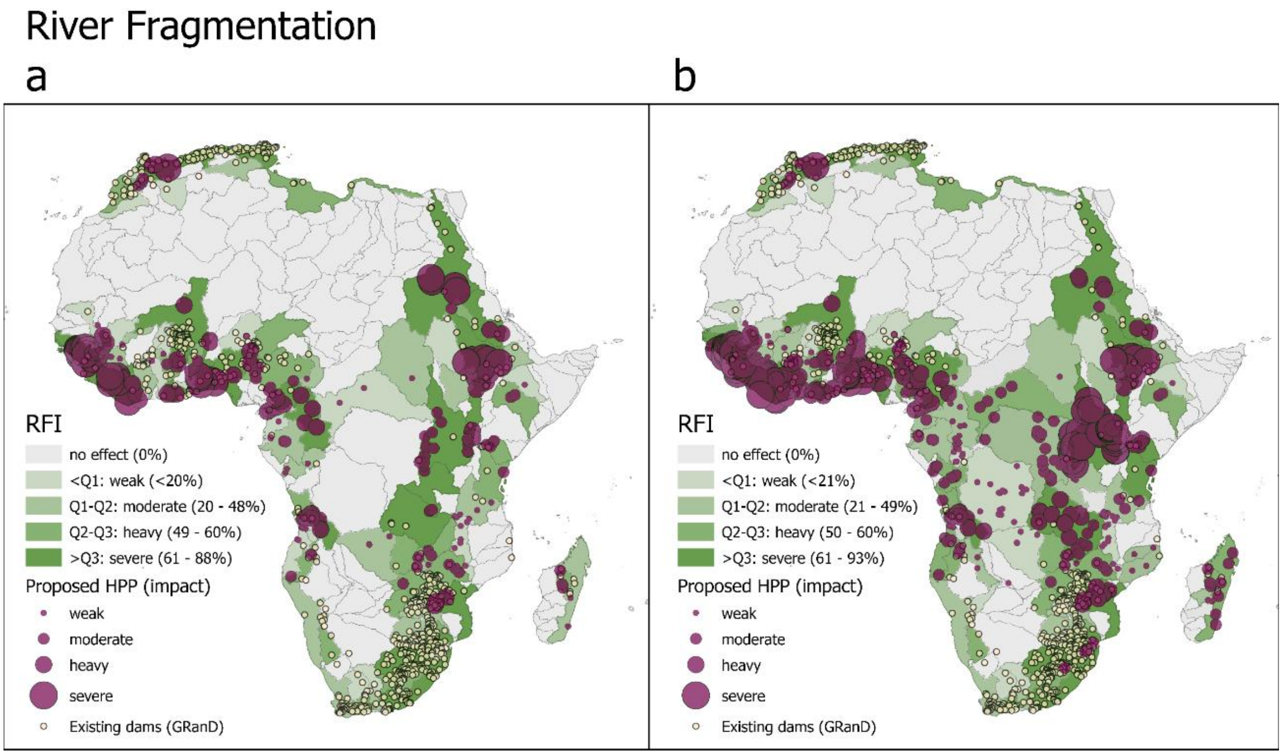

Figure S2: Results of impact assessment for the indicator River Fragmentation (RFI: River Regulation Index) for hydropower plants (HPPs) of type reservoir (a) and of type reservoir and unknown (b). Purple circles indicate proposed HPPs. Small yellow circles indicate existing dam infrastructure (GRanD). Impact of proposed HPPs increases with circle size. The base map shows RFI values divided according to the quartile values (Q) for sub-basins (HydroBASIN Level 4).

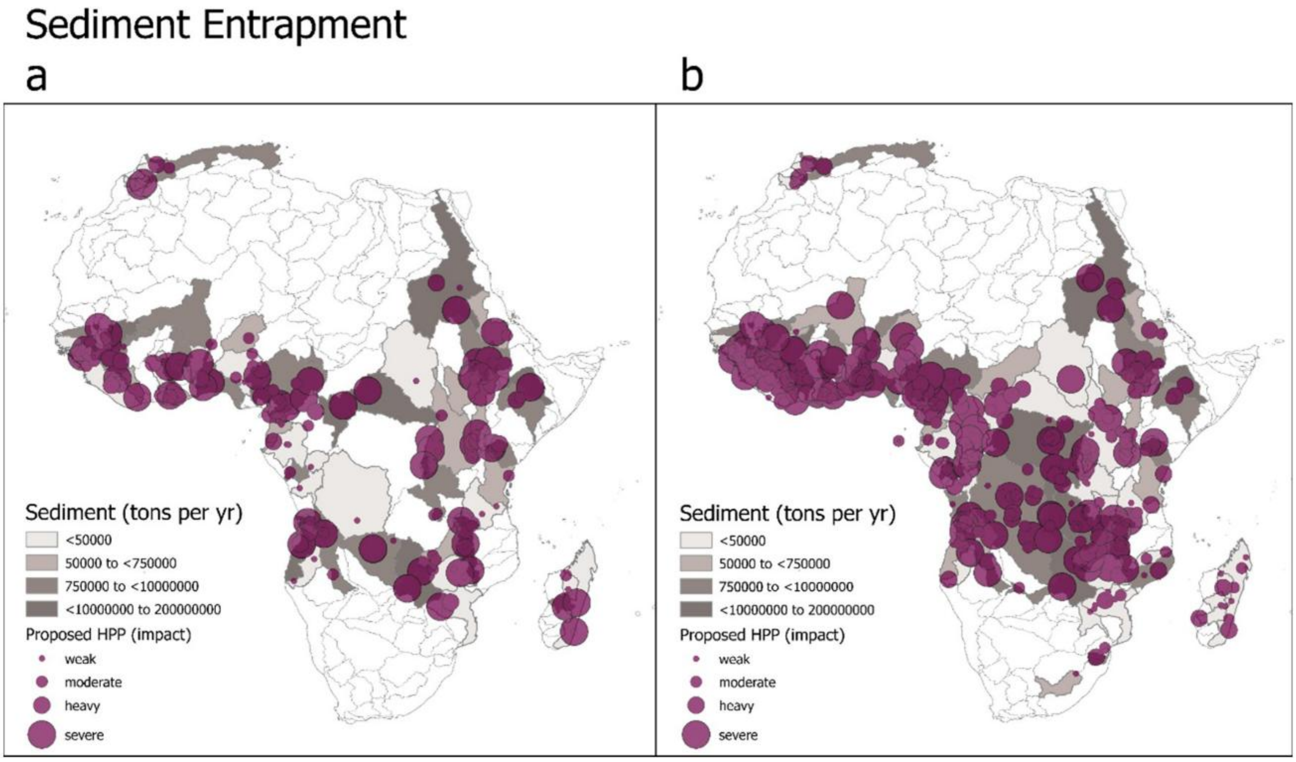

Figure S3: Results of impact assessment for the indicator Sediment Entrapment for hydropower plants (HPPs) of type reservoir (a) and of type reservoir and unknown (b). Purple circles indicate proposed HPPs. Impact of proposed HPPs increases with circle size. The base map shows the entrapped sediment per sub-basin (HydroBASIN, Level 10) [tons/year].

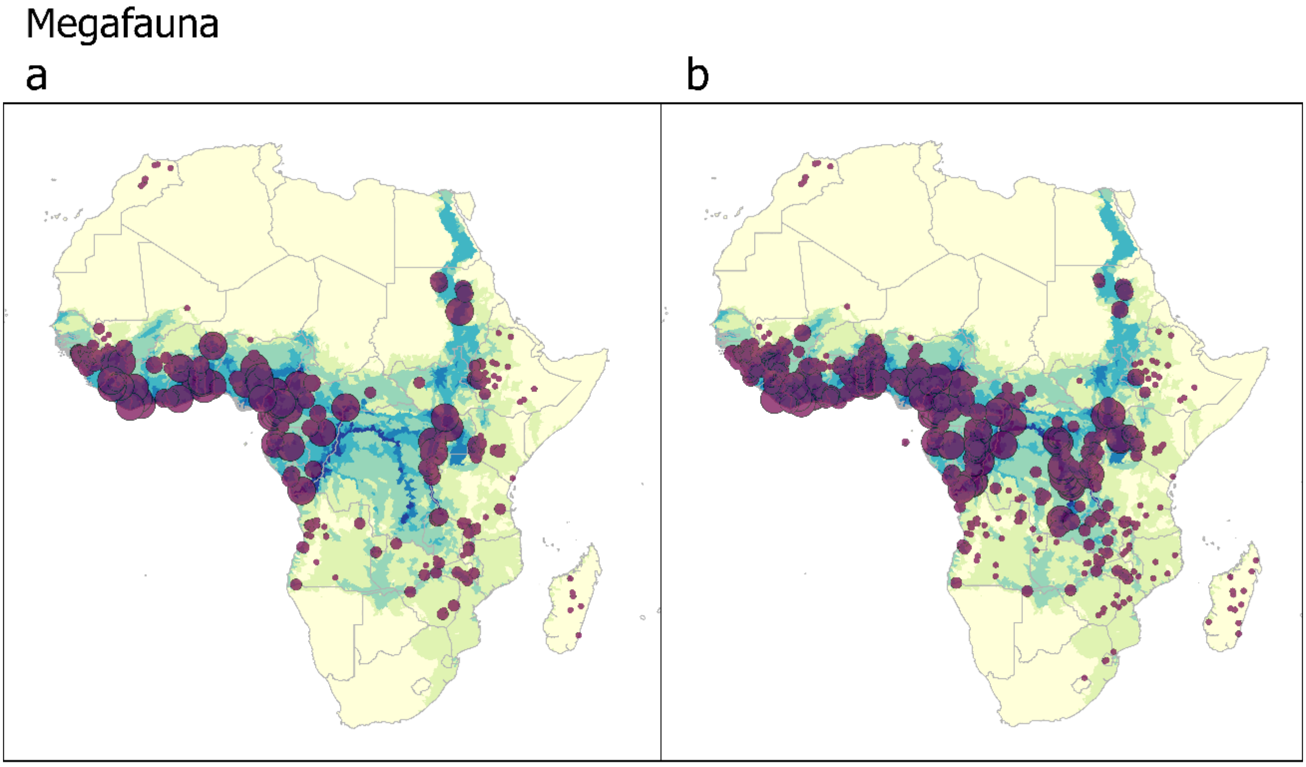

Figure S4: Results of impact assessment for the indicator Megafauna for hydropower plants (HPPs) of type reservoir (a) and of type reservoir and unknown (b). Purple circles indicate proposed HPPs. Impact of proposed HPPs increases with circle size. The base map shows the number of freshwater megafauna species. Colour intensity increases from yellow to dark blue with number of available megafauna species.

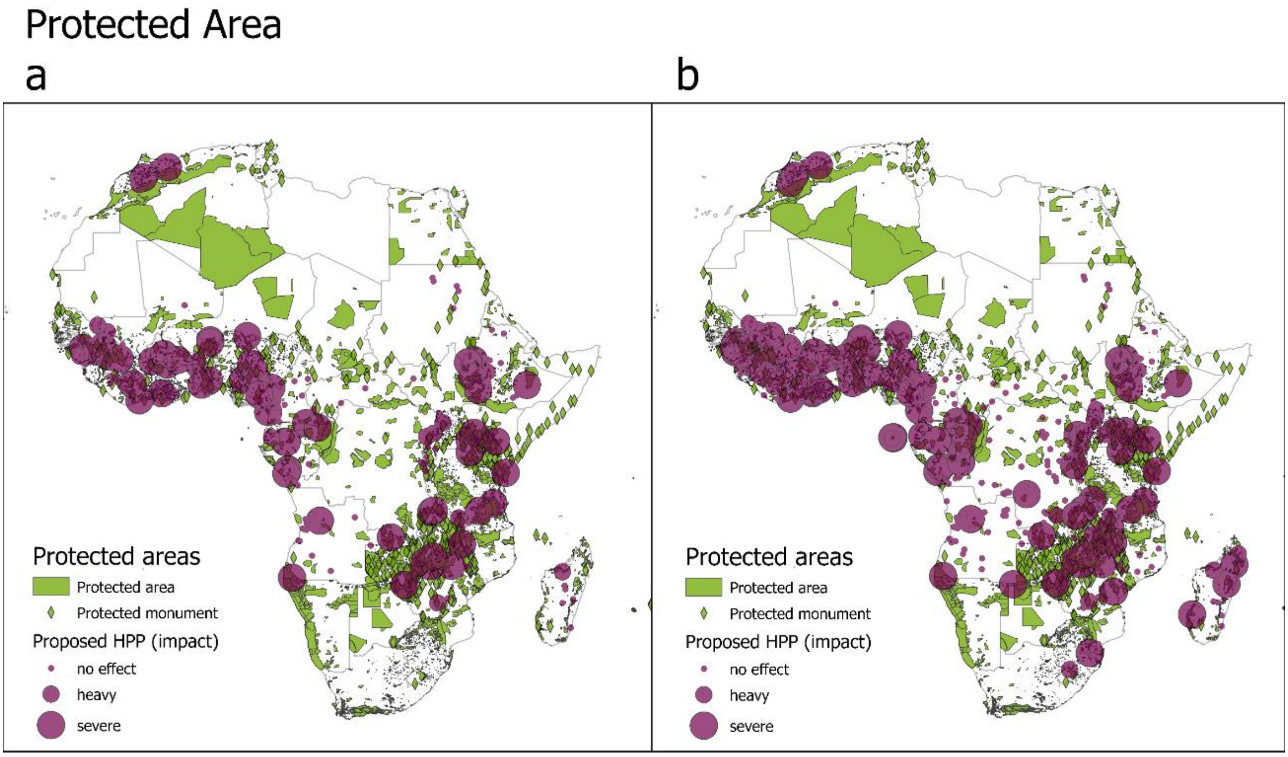

Figure S5: Results of impact assessment for the indicator Protected Area for hydropower plants (HPPs) of type reservoir (a) and of type reservoir and unknown (b). Purple circles indicate proposed HPPs. Impact of proposed HPPs increases with circle size. The base map shows protected areas and protected natural monuments (diamonds).

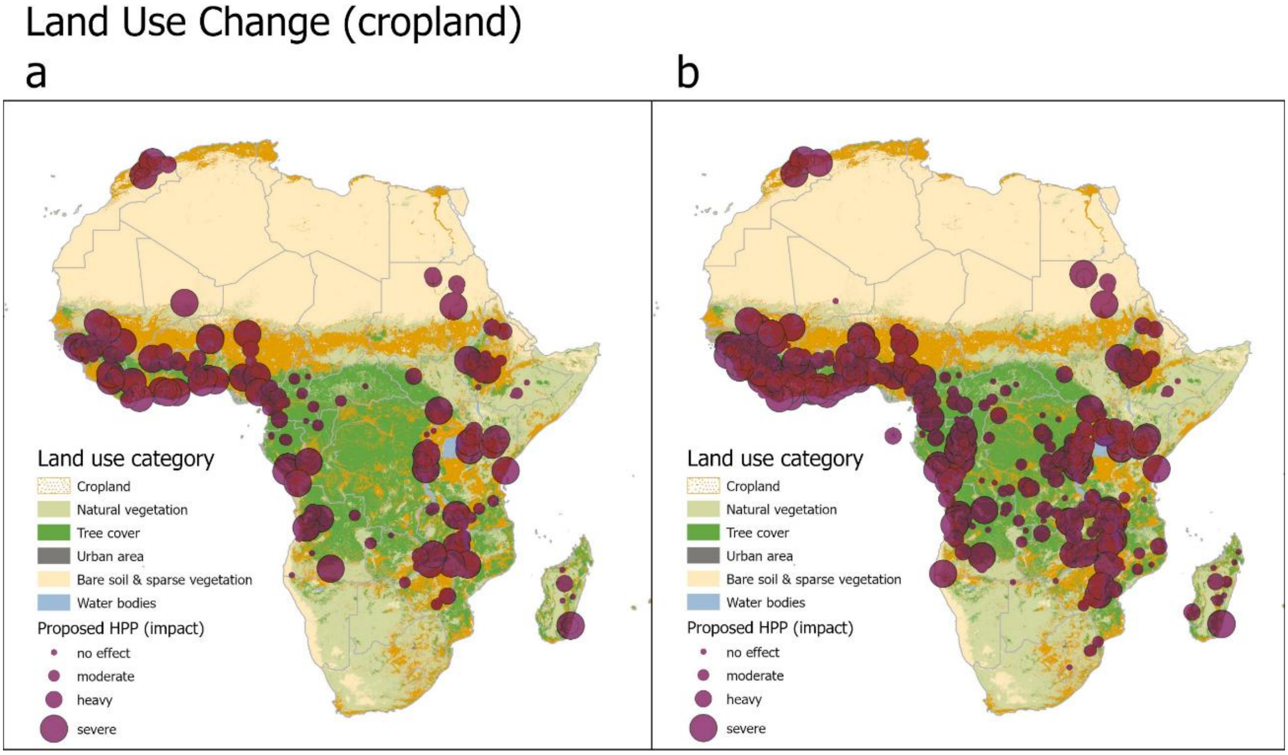

Figure S6: Results of impact assessment for the indicator Land Use Change (cropland) for hydropower plants (HPPs) of type reservoir (a) and of type reservoir and unknown (b). Purple circles indicate proposed HPPs. Impact of proposed HPPs increases with circle size. The base map shows the different land use categories. The impact assessment is based on the category cropland.

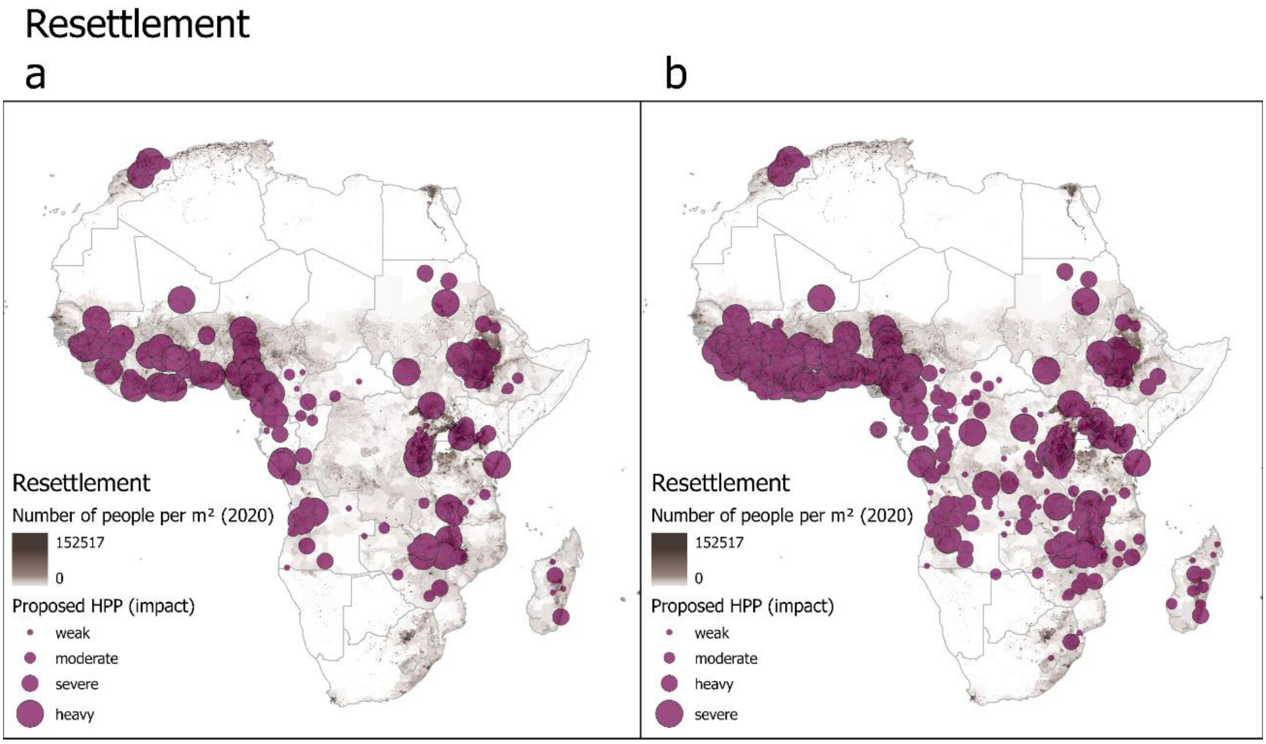

Figure S7: Results of impact assessment for the indicator Resettlement for hydropower plants (HPPs) of type reservoir (a) and of type reservoir and unknown (b). Purple circles indicate proposed HPPs. Impact of proposed HPPs increases with circle size. The base map shows the population density in 2020.

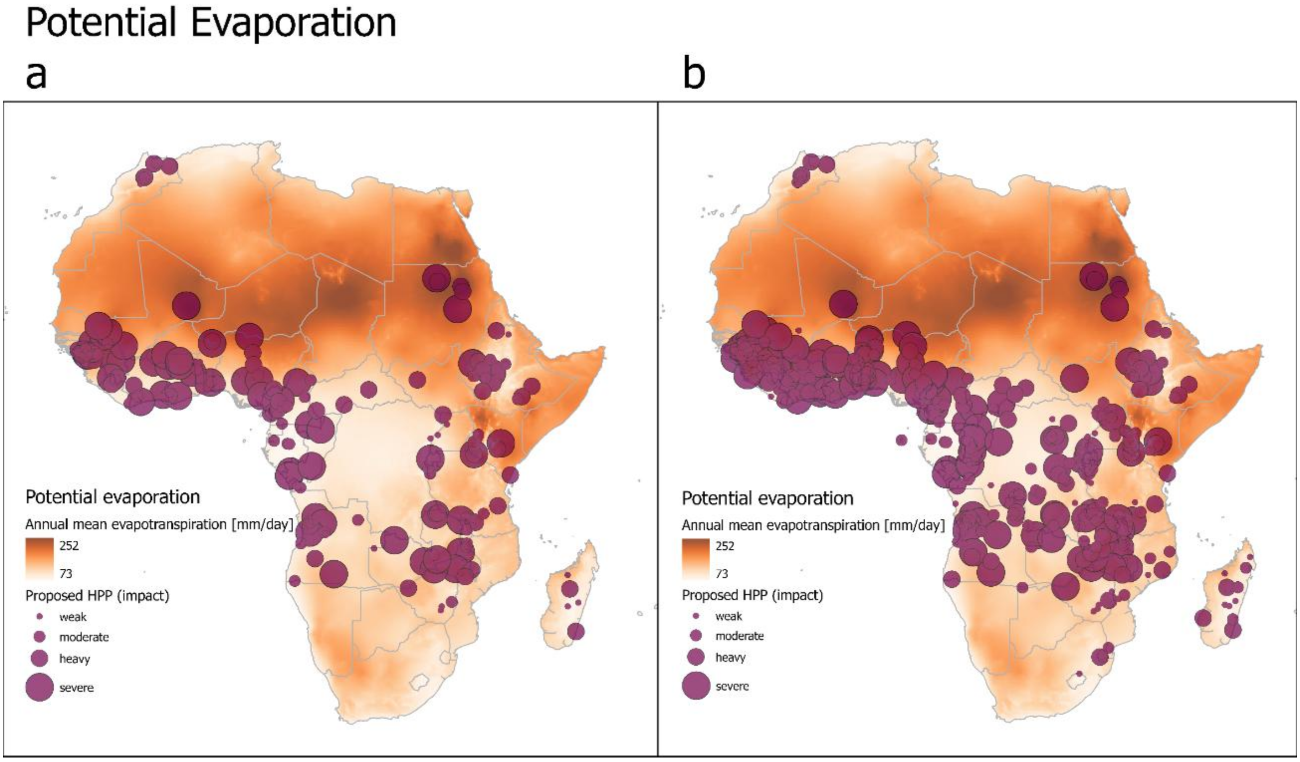

Figure S8: Results of impact assessment for the indicator Potential Evaporation for hydropower plants (HPPs) of type reservoir (a) and of type reservoir and unknown (b). Purple circles indicate proposed HPPs. Impact of proposed HPPs increases with circle size. The base map shows potential evaporation values [mm/year].

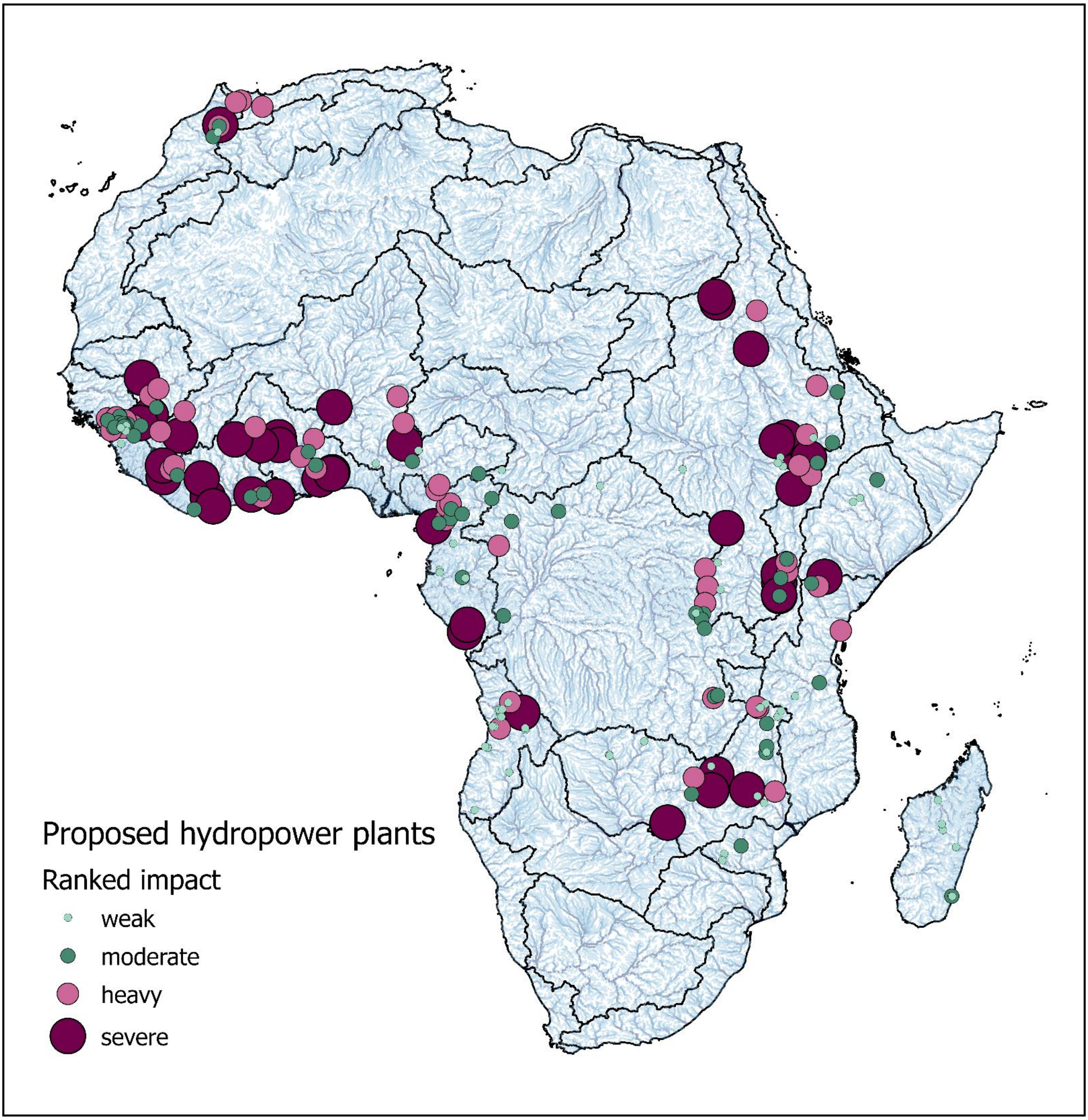

Figure S9: Results of the continental impact assessment including all indicators for hydropower plants of type reservoir (dataset 2): Circles indicate proposed hydropower plants and size increases with indicated quarter value from weak (<Quartile(Q) 1), moderate (Q1-Q2), heavy (Q2-Q3) to severe (>Q4). Boundaries represent major river basins (HydroBASIN Level 3).

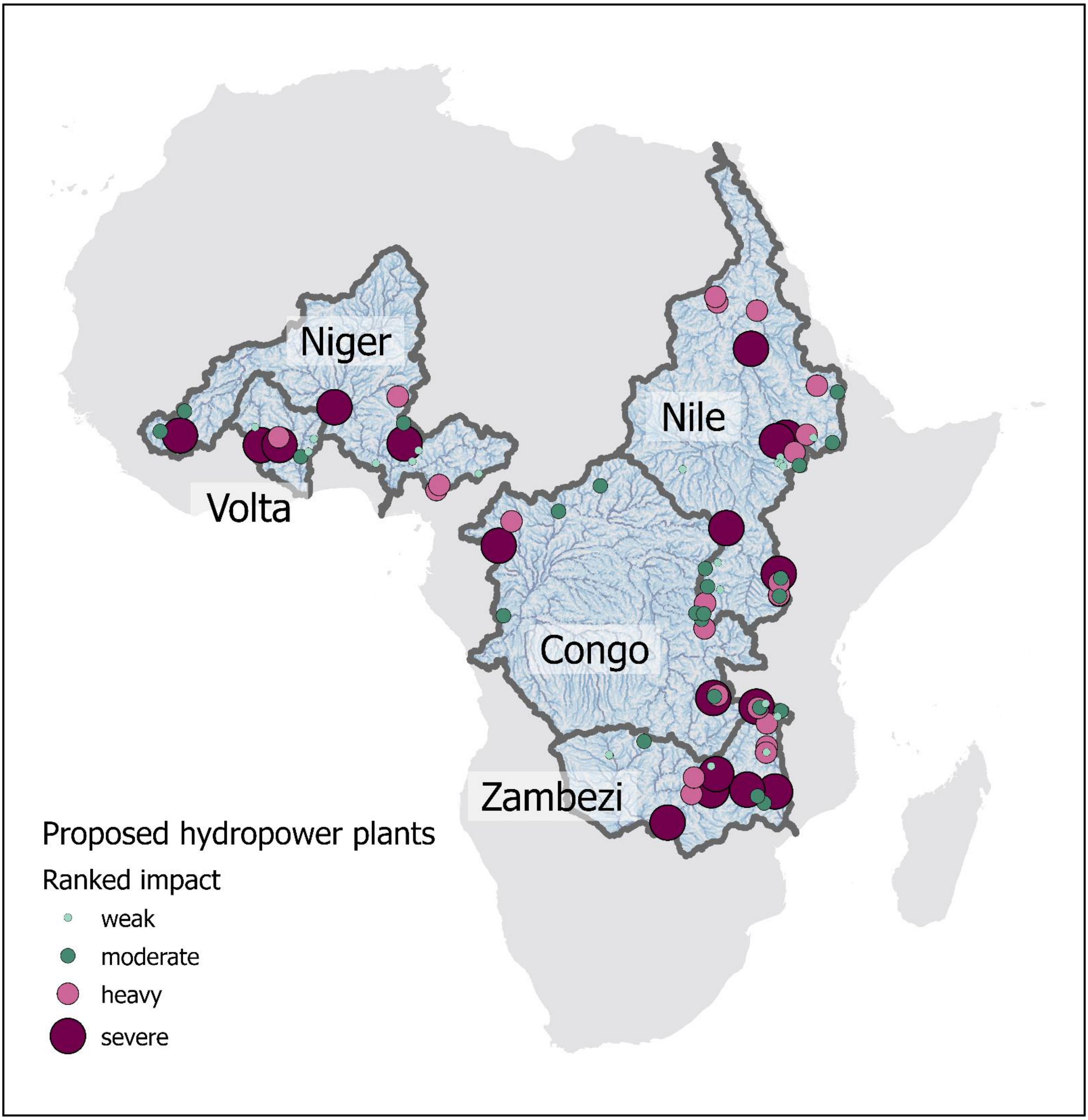

Figure S10: Results of the impact assessment at basin-scale including all indicators for hydropower plants of type reservoir (dataset 2). Basin-scale assessments were conducted for the Congo, Niger, Nile, Volta, and Zambezi river basins (HydroBASIN Level 3). Circles indicate proposed hydropower plants and circle size increases from weak (<Quartile(Q) 1), moderate (Q1-Q2), heavy (Q2-Q3) to severe (>Q4) cumulative impact.

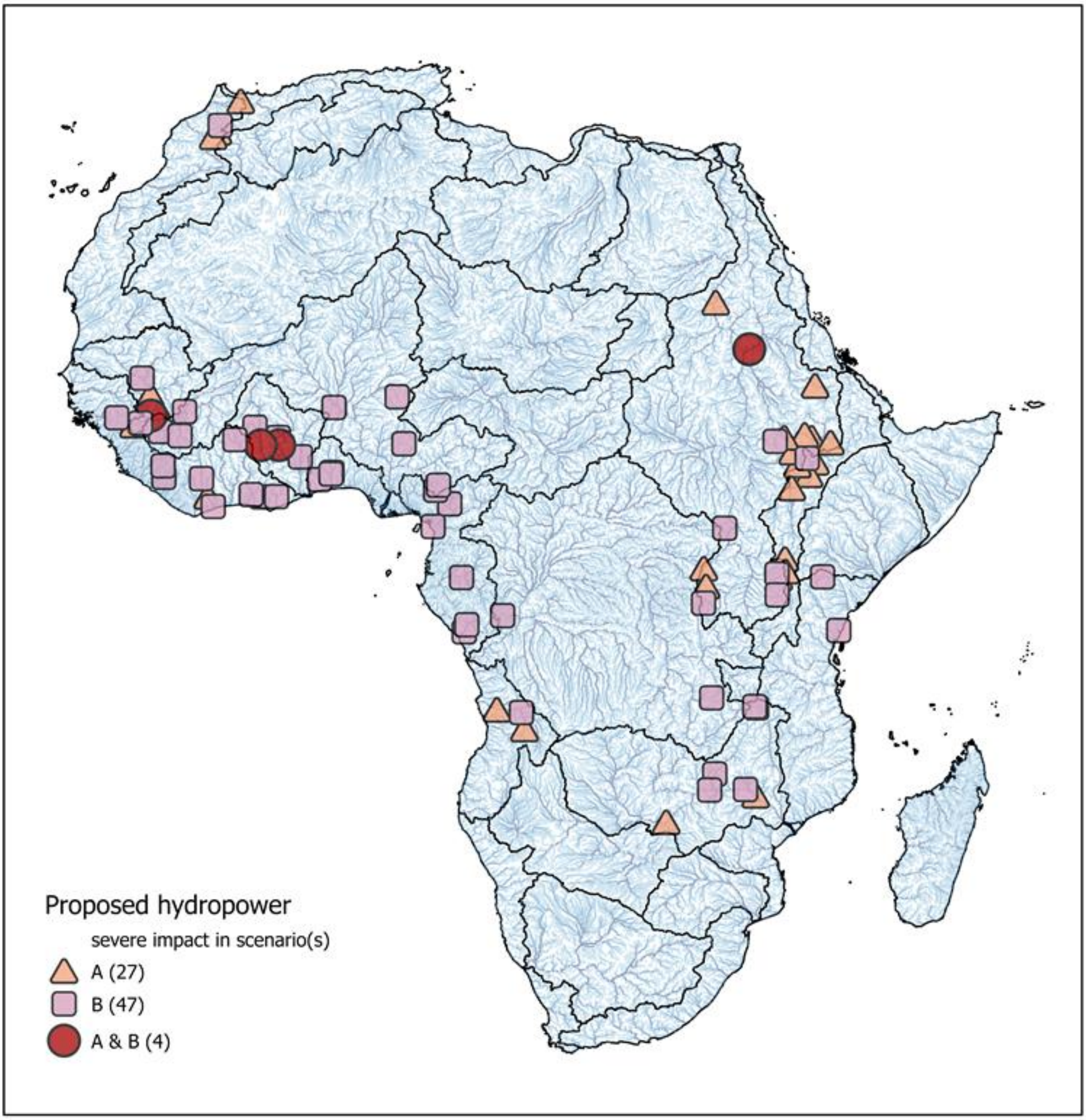

Figure S11: Results of the impact assessment for dataset 2 by applying three different scenarios of indicator consideration (Scenario A: Protected Area, Resettlement, Land Use Change, Megafauna, Sediment Entrapment; Scenario B: River Regulation, River Fragmentation, Potential Evaporation). Symbols indicate proposed hydropower plants in the top quarter (>Quartile 4; severe impact) of one (A: orange triangle, B: pink square), or both scenario(s) (red circle).

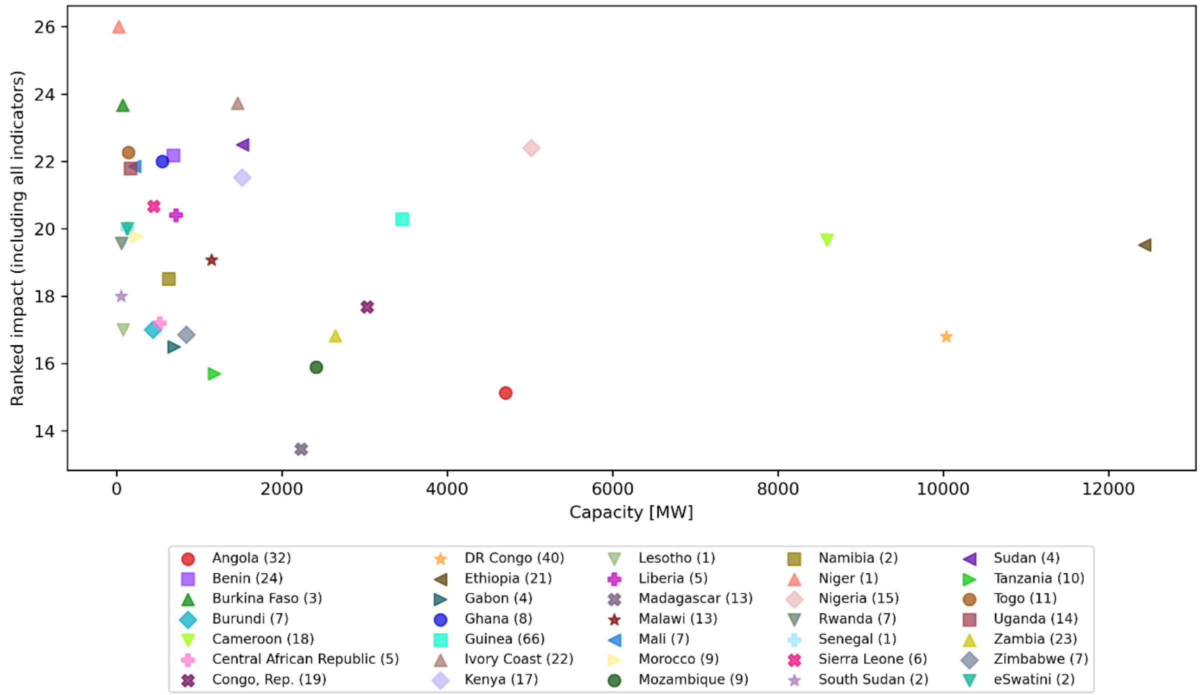

Figure S12: Summary of ranked impact (mean in numbers) per cumulative national capacity (sum in megawatts [MW]) at continental scale (all indicators) for hydropower plants of type reservoir (dataset 2): Countries vary by colour and symbol. The number of included hydropower plants is given in brackets.
